# The *Tug1* lncRNA locus is essential for male fertility

**DOI:** 10.1186/s13059-020-02081-5

**Published:** 2020-09-07

**Authors:** Jordan P. Lewandowski, Gabrijela Dumbović, Audrey R. Watson, Taeyoung Hwang, Emily Jacobs-Palmer, Nydia Chang, Christian Much, Kyle M. Turner, Christopher Kirby, Nimrod D. Rubinstein, Abigail F. Groff, Steve C. Liapis, Chiara Gerhardinger, Assaf Bester, Pier Paolo Pandolfi, John G. Clohessy, Hopi E. Hoekstra, Martin Sauvageau, John L. Rinn

**Affiliations:** 1 Department of Stem Cell and Regenerative Biology, Harvard University, Cambridge, MA 02138 USA; 2 BioFrontiers Institute, University of Colorado at Boulder, Boulder, CO 80303 USA; 3 Department of Biochemistry, University of Colorado at Boulder, Boulder, CO 80303 USA; 4 Department of Organismic and Evolutionary Biology, Harvard University, 16 Divinity Avenue, 4109 BioLabs, Cambridge, MA 02138 USA; 5 Department of Molecular and Cellular Biology, Harvard University, Cambridge, MA 02138 USA; 6 Department of Systems Biology, Harvard University, Cambridge, MA 02138 USA; 7 Cancer Research Institute, Beth Israel Deaconess Cancer Center, Department of Medicine and Pathology, Beth Israel Deaconess Medical Center, Harvard Medical School, Boston, MA 02215 USA; 8 Harvard Initiative for RNA Medicine, Boston, MA 02215 USA; 9 Howard Hughes Medical Institute, Chevy Chase, MD USA; 10 Montreal Clinical Research Institute, Montreal, QC H2W 1R7 Canada; 11 Department of Biochemistry and Molecular Medicine, Université de Montréal, Montreal, QC H3C 3J7 Canada

**Keywords:** *Tug1*, lncRNA, Fertility, DNA repressor, *Cis*-regulatory elements, RNA-seq, Allele-specific, Genetics, Genomics, Mouse

## Abstract

**Background:**

Several long noncoding RNAs (lncRNAs) have been shown to function as components of molecular machines that play fundamental roles in biology. While the number of annotated lncRNAs in mammalian genomes has greatly expanded, studying lncRNA function has been a challenge due to their diverse biological roles and because lncRNA loci can contain multiple molecular modes that may exert function.

**Results:**

We previously generated and characterized a cohort of 20 lncRNA loci knockout mice. Here, we extend this initial study and provide a more detailed analysis of the highly conserved lncRNA locus, taurine-upregulated gene 1 (Tug1). We report that Tug1-knockout male mice are sterile with underlying defects including a low number of sperm and abnormal sperm morphology. Because lncRNA loci can contain multiple modes of action, we wanted to determine which, if any, potential elements contained in the Tug1 genomic region have any activity. Using engineered mouse models and cell-based assays, we provide evidence that the Tug1 locus harbors two distinct noncoding regulatory activities, as a *cis*-DNA repressor that regulates neighboring genes and as a lncRNA that can regulate genes by a *trans*-based function. We also show that Tug1 contains an evolutionary conserved open reading frame that when overexpressed produces a stable protein which impacts mitochondrial membrane potential, suggesting a potential third coding function.

**Conclusions:**

Our results reveal an essential role for the Tug1 locus in male fertility and uncover evidence for distinct molecular modes in the Tug1 locus, thus highlighting the complexity present at lncRNA loci.

## Background

Noncoding RNAs have been shown to play central roles in biology. Key cellular machines such as telomerase and the ribosome are comprised of proteins and noncoding RNAs and serve as classic examples of RNA-based functionalities [1, 2]. While thousands of lncRNA loci have been identified in mammalian genomes, characterizing their functions has been a challenge because of their diverse biological roles and due to complications in identifying their modes of action [3, 4]. Indeed, in addition to RNA function, lncRNA loci can harbor several potential regulatory modalities including DNA regulatory elements and the act of transcription [5–12]. Moreover, lncRNAs have been found to possess open reading frames (ORFs) [13, 14], and an increasing number have been found to encode proteins with biological roles [15–19]. With this in mind, it is likely that more regulatory DNA, RNA, and hidden protein activities will be uncovered at lncRNA loci. Thus, identifying the molecular activities present at lncRNA loci is important for further functional dissection of phenotypes attributed to lncRNA loci.

We previously reported the generation of 20 lncRNA loci knockout mouse strains, five of which displayed either viability, growth, or brain phenotypes [20, 21]. From the strains that did not initially display such phenotypes, we selected *Tug1* for further analysis because of its high conservation [22] as well as implications in diverse cellular functions and human malignancies [23].

*Tug1* was first identified in a microarray screen for genes upregulated in response to taurine in a heterogenous culture of retinal cells [22]. In addition, there is some evidence that *Tug1* is transcriptionally regulated by p53 [24]. A number of studies have found evidence suggesting diverse RNA-based roles for *Tug1*, including but not limited to the development of photoreceptors [22] and in regulating gene expression in the nucleus by associating with the polycomb repressive complex 2 (PRC2) [25–27]. There is also some evidence for an RNA-based role for *Tug1* in cancer by acting as a tumor suppressor in human gliomas [28, 29] and by acting as a cytoplasmic miRNA sponge in prostate cancer cell lines [30]. Thus, the number of studies identifying broad roles for *Tug1* underscores the importance of this locus.

Here, we characterize the *Tug1* locus using multiple genetic approaches and describe a physiological function in spermatogenesis and male fertility. We show that deletion of the *Tug1* locus in mice leads to male sterility and also report an underappreciated molecular complexity at the *Tug1* locus. Using several complementary genetic approaches (gene body deletion with a *lacZ* reporter knock-in, an inducible *Tug1* transgene, and combinations thereof), we provide evidence of a DNA-based repressive element within the *Tug1* locus that regulates several genes in *cis*. We show that a gene expression program dysregulated in *Tug1*-knockout testes can be partially rescued by ectopic expression of *Tug1* RNA in vivo. Finally, we show that the *Tug1* locus contains an evolutionarily conserved ORF, which can be translated into a stable protein that impacts mitochondrial membrane potential upon overexpression. Collectively, this study implicates the *Tug1* locus as essential in male fertility and provides evidence that the *Tug1* locus contains at least two noncoding regulatory activities and a putative coding function.

## Results

### The *Tug1* lncRNA locus is widely expressed and highly conserved

**Fig. 1:**
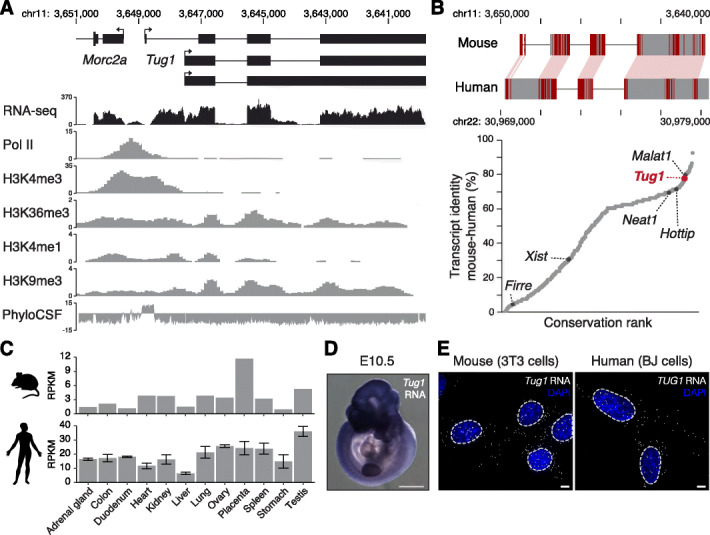
The *Tug1* lncRNA locus is highly conserved and ubiquitously expressed. **a** *Tug1* mouse genomic locus (shown inverted). UCSC Genome Browser tracks for RNA sequencing (RNA-seq), RNA polymerase II (Pol II), histone 3 lysine 4-trimethylation (H3K4me3), H3K36me3, and H3K4me1 occupancy in the testis and H3K9me3 occupancy in the brain are depicted. PhyloCSF scores across the locus are shown. Chromosomal coordinates of the mouse *Tug1* gene are indicated (mm9). **b** Upper panel: schematic of the nucleotide conservation alignment for mouse and human *Tug1/TUG1*. Red lines indicate conserved nucleotides. Chromosomal coordinates of the *Tug1/TUG1* gene for both species are indicated. Lower panel: distribution of sequence identity for orthologous divergent and intergenic lncRNAs between mice and humans. The *x*-axis shows increasing conservation rank. *Tug1* and other well-characterized lncRNAs are highlighted. **c** RNA-seq expression levels of *Tug1* in a panel of mouse and human tissues. **d** RNA in situ hybridization of *Tug1* RNA in a mouse embryo at embryonic day 10.5 (E10.5). **e** Maximum intensity projections of *Tug1* single-molecule RNA FISH (gray) on murine 3T3 and human BJ fibroblasts. The nucleus is stained with DAPI (blue). Scale bar is 5 μm

The murine *Tug1* lncRNA locus is located on chromosome 11 and has three annotated transcripts (Fig. 1a). *Tug1* shares a bidirectional promoter with its neighboring protein-coding gene *Morc2a*, whose transcription start site (TSS) is located approximately 680 bp upstream of the first *Tug1* TSS. The *Tug1* locus is enriched with hallmarks of active transcription, such as RNA polymerase II (Pol II) and histone H3 lysine 4-trimethylation (H3K4me3) at its promoter, H3K36me3 across its gene body, and abundant transcription as shown by RNA-seq (Fig. 1a). However, the *Tug1* locus is simultaneously enriched with the repressive histone mark H3K9me3 in several mouse cell types (Fig. 1a and Additional file 1: Fig. S1). This atypical combination of H3K9me3 and H3K36me3 histone marks at the *Tug1* locus is also conserved in human cells (Additional file 1: Fig. S1). Moreover, the binding of repressor proteins SIN3A and COREST has been detected at both the human and mouse promoters (Additional file 1: Fig. S1).

*Tug1* is among the most conserved lncRNAs between human and mouse, with exonic nucleotide conservation levels reaching 77% (Fig. 1b). This level of sequence conservation is similar to the highly abundant lncRNA *Malat1* (79%) and higher than other well-characterized lncRNAs including *Hottip* (71%), *Neat1* (69%), *Xist* (30%), and *Firre* (4%) (Fig. 1b) [31]. Further conservation analyses lead us to identify a highly conserved open reading frame (ORF) in the *Tug1* locus, as indicated by phylogenetic codon substitution frequencies (PhyloCSF) (Fig. 1a), a computational tool that can distinguish protein-coding and noncoding regions [32].

In addition to its high level of sequence conservation, *Tug1* RNA is expressed at moderate to high levels in several adult tissues in both mouse and human (Fig. 1c) [33, 34], and *Tug1* RNA is detected in a number of embryonic tissues at multiple embryonic stages (E8.0–E12.5) (Fig. 1d and Additional file 2: Fig. S2). Further, using single-molecule RNA fluorescence in situ hybridization (smFISH), we observed that *Tug1* RNA is detected in both the cytoplasm and the nucleus in human and mouse fibroblasts (Fig. 1e), which is consistent with previous reports [24, 35–37].

### *Tug1*^−/−^ males are sterile due to impaired spermatogenesis

**Fig. 2:**
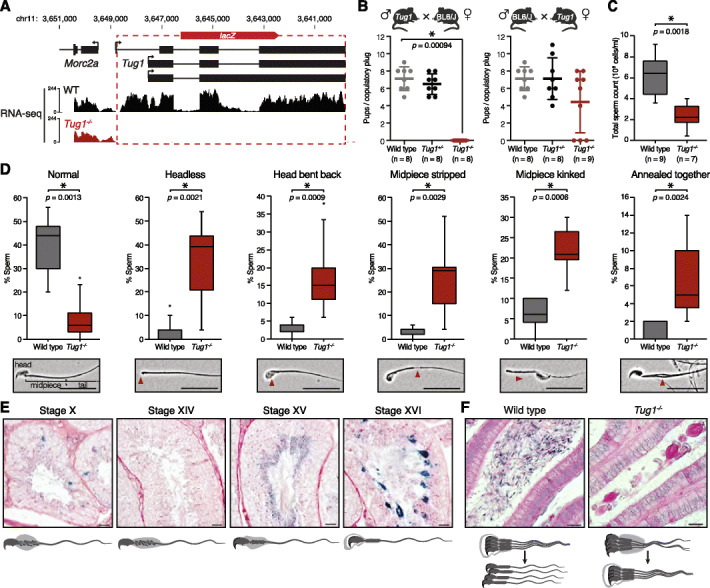
Deletion of the *Tug1* locus leads to sperm defects and male infertility. **a** Deletion strategy of the *Tug1* locus (shown inverted). The *Tug1* gene body was replaced by a *lacZ* reporter cassette, leaving the promoter and first exon intact. The dashed lines indicate the deleted region in the *Tug1* knockout. RNA sequencing (RNA-seq) tracks for wild-type (WT) and *Tug1*^*−/−*^ testis are depicted. **b** Scatter dot plot (showing the mean and standard deviation) of the number of pups at birth per copulatory plug for matings between wild-type, *Tug1*^*+/−*^, or *Tug1*^*−/−*^ males and wild-type C57BL/6J females (left panel) and wild-type C57BL/6J males and wild-type, *Tug1*^*+/−*^, or *Tug1*^*−/−*^ females (right panel). Each dot represents a litter from a different mouse. Significant (*) *p* value (Wilcoxon rank-sum test with Bonferroni correction) and the number of mice for each genotype tested are indicated. **c** Box plot of total sperm count for wild type and *Tug1*^*−/−*^ males. Significant (*) *p* value (Wilcoxon rank-sum test) is indicated. **d** Box plots of the percentage of normal sperm and sperm with the five most common morphological abnormalities for wild-type (*n* = 9) and *Tug1*^*−/−*^ (*n* = 8) males. Representative images of normal and morphologically aberrant sperm are located below each corresponding plot. Red arrows indicate the location of the defect. Scale bars are 20 μm. Significant (*) *p* values (Wilcoxon rank-sum test) are indicated. **e** Representative spermatocyte diagrams and micrographs of *Tug1*^*+/−*^ seminiferous tubule sections stained with periodic acid-Schiff’s reagent and X-gal showing expression of the *lacZ* reporter under the control of the endogenous *Tug1* promoter at the indicated stages of spermatogenesis. Scale bars are 20 μm. **f** Representative spermatid diagrams and micrographs of wild-type and *Tug1*^*−/−*^ epididymis tubule sections stained with hematoxylin and eosin. Scale bars are 20 μm

To investigate the in vivo role of *Tug1*, we utilized a *Tug1*-knockout (*Tug1*^*−/−*^) mouse model where the gene body of the *Tug1* locus was removed and replaced with a *lacZ* reporter cassette downstream of the endogenous promoter, thereby preserving the act of transcription (Fig. 2a) [20, 21]. Notably, this deletion strategy also removed 86 out of 143 amino acids in the predicted ORF (Additional file 3: Fig. S3). Thus, using this approach, any potential phenotype due to the lncRNA, potential DNA elements, or even a putative protein would be included. We confirmed the *Tug1* genetic model by genotyping and by RNA-seq analysis in wild-type and *Tug1*^*−/−*^ testes (Fig. 2a).

*Tug1*^−/−^ mice are viable and do not display any obvious physiological abnormalities up to 1 year of age, with the exception of a slight reduction in weight in male mice relative to wild-type littermates (Additional file 4: Fig. S4A). As previously reported, the progeny of *Tug1*^*+/−*^ intercrosses follow normal Mendelian ratios [20]. However, we noticed a complete absence of offspring from intercrosses between *Tug1*^*−/−*^ mice (*n* = 4 breeding pairs). Therefore, we sought to investigate the fertility of *Tug1*^−/−^ mutants in more detail. We separately mated *Tug1*^*−/−*^, *Tug1*^*+/−*^, and wild-type males or females to *C57BL/6J* mice. We did not observe a difference in the mounting behavior between wild-type and *Tug1*^−/−^ mice, as assessed by the presence of a vaginal plug. Strikingly, matings between *Tug1*^*−/−*^ males (*n* = 8) and *C57BL/6J* females did not produce any offspring, whereas matings involving either *Tug1*^*+/−*^ males (*n* = 8) or wild-type males (*n* = 8) with *C57BL/6J* females resulted in similar numbers of offspring (Fig. 2b). Moreover, 6 out of 9 *Tug1*^*−/−*^ females that mated with *C57BL/6J* males gave birth to pups (Fig. 2b), indicating that only *Tug1*^*−/−*^ males are sterile.

To further understand the underlying fertility defect in *Tug1*^−/−^ males, we examined the reproductive morphology of wild-type and *Tug1*^−/−^ male mice. Testicular descent appeared normal, and we did not observe any other gross morphological abnormalities in their reproductive system upon dissection (Additional file 4: Fig. S4B). We measured testis mass relative to total body weight and did not observe a significant decrease (*p* = 0.0751) in *Tug1*^−/−^ (mean = 0.25 ± 0.020%, *n* = 8) compared to wild type (mean = 0.30 ± 0.016%, *n* = 9) (Additional file 4: Fig. S4C). Next, we quantified sperm production and found a significant reduction in sperm number from *Tug1*^−/−^ males (mean = 2.35 × 10^6^ ± 0.473 × 10^6^ cells/mL, *n* = 7), which produced on average only 40% as many sperm as wild-type mice (6.13 × 10^6^ ± 0.636 × 10^6^ cells/mL, *n* = 9, *p* = 0.0018) (Fig. 2c). Although *Tug1*^−/−^ males produce fewer sperm, none was found to completely lack sperm (a condition called azoospermia).

Based on these results, we investigated whether perturbations in sperm morphology could also contribute to the complete sterility observed in *Tug1*^−/−^ males. We examined the morphological features of sperm and quantified the frequency of 15 different abnormalities (Additional file 5: Table S1). Overall, the proportion of morphologically normal sperm was significantly lower in *Tug1*^*−/−*^ mice (mean = 8.3 ± 3.0%, *n* = 8, *p* = 0.0013) compared to wild-type males (mean = 38.9 ± 4.3%, *n* = 9) (Fig. 2d). We observed significant morphological defects in *Tug1*^−/−^ sperm including sperm with no head, misshapen head, head bent back, stripped midpiece, kinked midpiece, curled midpiece, midpiece debris, broken tail, and the presence of multiple sperm attached along the midpiece (Fig. 2d, Additional file 4: Fig. S4D, and Additional file 5: Table S1). Together, these results indicate that the sterility of *Tug1*^−/−^ males arises from a combination of low sperm count (oligozoospermia) and abnormal sperm morphology (teratozoospermia).

To further investigate how the deletion of the *Tug1* locus leads to abnormal sperm morphology, we examined the timing of *Tug1* expression at different stages of spermatogenesis. To this end, we took advantage of the knock-in *lacZ* reporter driven by the endogenous *Tug1* promoter and assessed the expression by *lacZ* staining of histological sections of *Tug1*^*+/−*^ testis and epididymis. From stages IX to XI of spermatogenesis in the testis, *lacZ* staining was restricted to the excess cytoplasm, known as residual bodies, which are phagocytosed toward the basement membrane by Sertoli cells (Fig. 2e) [38]. No expression was detected in the later stages XII to XIV (Fig. 2e). However, we observed *lacZ* staining in stage XV elongated spermatids, and the *lacZ* staining appeared stronger at stage XVI, just before spermiation (Fig. 2e). The observed *lacZ* pattern indicates that *Tug1* expression is temporally controlled during spermatogenesis.

In *Tug1*^−/−^ testes, mature spermatids appeared to remain attached by their collective cytoplasm. This was even more striking in the epididymis, where multiple sperm aggregates were observed in *Tug1*^−/−^ mice, while individual sperm appeared to migrate freely throughout the lumen in wild-type mice (Fig. 2f). These aggregates were present in all regions of the epididymis (caput, corpus, and cauda). Consistent with the reduced sperm count, fewer individual sperm were observed in *Tug1*^−/−^ epididymis tissue compared to wild type. Together, our analyses of the *Tug1*^−/−^ mouse model provide evidence that the locus is required for male fertility.

### *Tug1* DNA encodes a *cis* repressor regulatory element

**Fig. 3:**
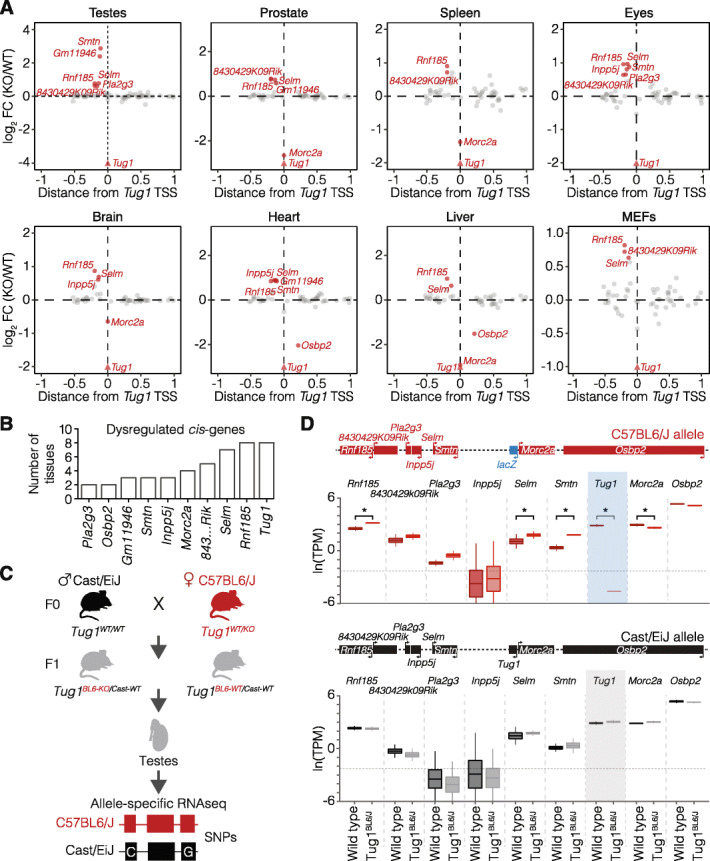
The *Tug1* locus harbors a *cis*-repressive DNA regulatory element. **a** Differential expression of genes in the local region (± 1 Mb) of *Tug1* for each indicated mouse tissue, depicted as fold change (FC) between *Tug1*^*−/−*^ (KO) and wild-type (WT). Significantly differentially expressed genes are marked and labeled in red. **b** Plot of the number of tissues where genes downstream of *Tug1* TSS are found significantly dysregulated. **c** Strategy for allele-specific RNA sequencing (RNA-seq). *Tug1*^*+/−*^ C57BL/6J females were crossed with wild-type Cast/EiJ males, and the testes from the F1 hybrid progeny were harvested for RNA-seq. Single nucleotide polymorphisms (SNPs) allowed for the differentiation between the C57BL/6J and the Cast/EiJ allele. **d** Allele-specific expression of local genes surrounding *Tug1* in the testes from F1 hybrid C57BL/6J::Cast/EiJ wild-type (*Tug1*^*BL6-WT/Cast-WT*^) and heterozygous *Tug1*-knockout (*Tug1*^*BL6-KO/Cast-WT*^) mice containing a deletion on the C57BL/6J allele. Upper panel: expression levels of neighboring genes from the C57BL/6J allele. Lower panel: expression levels of neighboring genes from the Cast/EiJ allele. Boxes are centered at the mean, extend one standard deviation, and the bottom and top notches are the minimum and maximum samples, respectively. The genomic locus encompassing the local genes around *Tug1* is depicted. Asterisks indicate significant Bayesian posterior probability (PP > 0.95) differential expression between hybrid wild-type and *Tug1*^*BL6-KO*^ testes. Horizontal dotted line indicates expression levels below 0.1 TPM

We next sought to investigate what, if any, molecular activities (DNA, lncRNA, or protein) are present at the *Tug1* locus. Many lncRNA loci have been reported to contain DNA regulatory elements that can regulate the expression of neighboring genes (*cis*-acting) [10–12]. The *Tug1*^−/−^ model enables us to test for potential *cis*-regulatory activity within the *Tug1* locus because the gene-ablation design removes potential *cis*-acting elements yet keeps the act of transcription intact (Fig. 2a) [20, 21]. To determine if there is a local regulatory effect on gene expression, we performed RNA-seq on the testes from wild-type and *Tug1*^−/−^ mice and plotted significant changes in gene expression within a 2-Mb region centered on the *Tug1* locus (FDR < 0.05, FC > 1.5) (Additional file 6: Table S2). Of the 71 genes within this window, we observed that 6 genes (*Rnf185*, *Pla2g3*, *Selm*, *Smtn*, *Gm11946*, and *8430429K09Rik*) were significantly upregulated in *Tug1*^−/−^ testes compared to wild type (Fig. 3a). Notably, these genes are downstream of the *Tug1* TSS and located within the same topological associated domain (TAD) in embryonic stem cells [39, 40].

To further investigate whether the *cis*-effect upon deletion of the *Tug1* locus is more widespread, we performed RNA-seq on 6 additional tissues (prostate, spleen, eyes, heart, liver, and mouse embryonic fibroblasts (MEFs)) as well as re-analyzed an existing brain dataset from wild-type and *Tug1*^−/−^ mice (Additional file 7: Table S3) [41]. Of the 71 genes within the 2-Mb region centered on the *Tug1* locus (FDR < 0.05, FC > 1.5), 9 genes were dysregulated in one or more tissues (7 upregulated and 2 downregulated) (Fig. 3a). Of the 7 upregulated genes, the E3 ubiquitin ligase, *Rnf185*, was consistently upregulated in 8 of 8 *Tug1*^−/−^ tissues, followed by the selenoprotein M gene, *Selm* (7 of 8 samples), and *8430429K09Rik* (6 of 8 samples) (Fig. 3b). We also note that *Morc2a*, the protein-coding gene that shares a promoter with *Tug1*, was significantly downregulated in 4 of the 8 samples, but this effect was not observed in the testes (Fig. 3a). Collectively, these data provide evidence of a *cis* repressor function at the *Tug1* locus in a broad range of tissues.

Since the neighboring genes are upregulated upon deletion of the *Tug1* locus, we reasoned that the repressive activity could be mediated either directly by the *Tug1* transcript or by regulatory DNA elements within the locus. To determine if the repressive effect of *Tug1* on neighboring genes occurs on the same allele (*cis-*acting), we performed allele-specific RNA-seq using a hybrid mouse strain. To generate this strain, we crossed *Tug1*^*+/−*^ C57BL/6J females with *Mus castaneus* (Cast/EiJ) males (Fig. 3c). The resulting polymorphisms in the F1 hybrid progeny (~ 1/150 bp between C57BL/6J and Cast/EiJ) allow quantification of gene expression from each strain-specific allele [42]. We thus harvested the testes from F1 hybrid males harboring a maternal C57BL/6J allele deletion and performed allele-specific expression analysis (Fig. 3c; Additional file 8: Table S4; Additional file 9: Table S5). As a control for haplotype-specific effects, we also analyzed allele-specific expression differences in wild-type F1 hybrid C57BL/6J::Cast/EiJ male littermates. We then quantified the expression from each allele and found that *Rnf185*, *Selm*, and *Smtn* were significantly upregulated and *Morc2a* slightly downregulated only on the C57BL/6J allele containing the *Tug1* deletion (Fig. 3d). Importantly, no change in the expression was detected from any gene within 1 Mb of *Tug1* on the Cast/EiJ allele, which contains an intact *Tug1* locus (Fig. 3d). Moreover, it is notable that *Tug1* RNA from the intact allele does not impact the dysregulated genes found on the *Tug1*-knockout allele, thereby suggesting a DNA-based repressor role at the *Tug1* locus. From these different mouse models, we conclude that the *Tug1* DNA, rather than the lncRNA or the act of transcription, exerts a repressive effect in *cis* on several genes up to 200 kb downstream of the *Tug1* transcription site.

### *Tug1* lncRNA regulates gene expression in *trans*

**Fig. 4:**
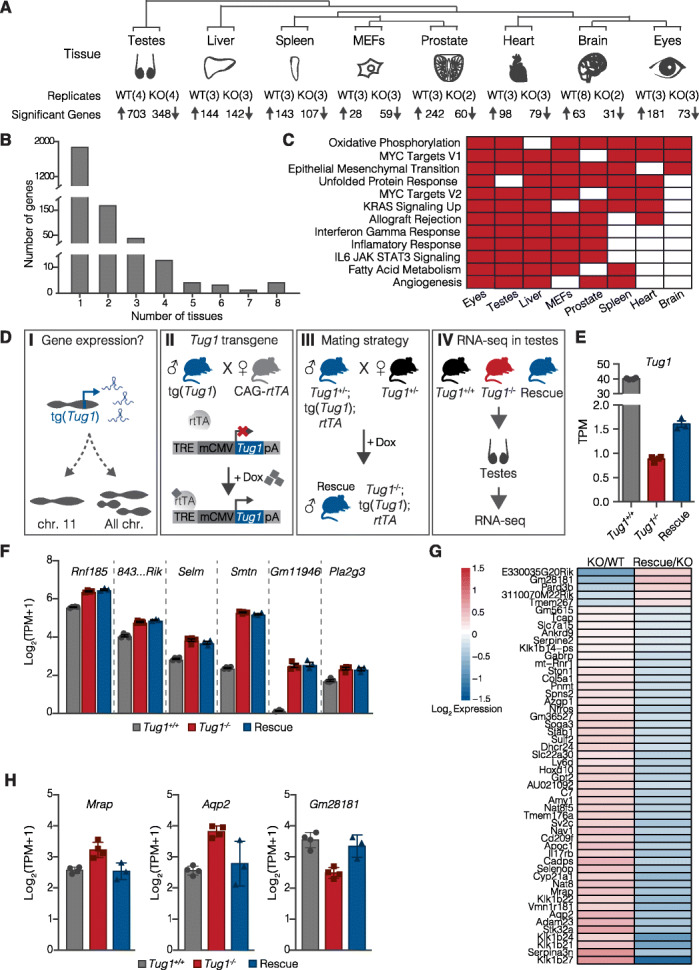
*Tug1* lncRNA regulates gene expression in *trans*. **a** Adult tissue types and mouse embryonic fibroblasts (MEFs) used for RNA sequencing of wild-type (WT) and *Tug1*^*−/−*^ (KO) mice. For each tissue, the number of biological replicates per genotype and the number of upregulated and downregulated genes (FDR < 0.05) are shown from KO to WT comparisons. **b** The number of perturbed genes (*y*-axis) in KO animals according to the number of tissues in which the gene was found to be dysregulated (*x*-axis). **c** Gene set enrichment analysis (GSEA) of differentially expressed genes found in wild-type versus *Tug1*^−/−^ murine tissues and MEFs. Red shading indicates tissue in which gene set is perturbed; gray shading indicates tissue in which gene set is not different between WT and KO. **d** Schematic showing the experimental design to identify genes reciprocally regulated by *Tug1* RNA. (I) Testing the impact of the *Tug1* transgene expression on gene expression in vivo. (II) Schematic of the *Tug1* transgene (tg(*Tug1*)) and systemic induction by mating to CAG-rtTA3 mice in the presence of doxycycline (dox). (III) Schematic of matings to generate *Tug1*^rescue^ mice (*Tug1*^−/−^; tg(*Tug1*); *rtTA*), enabling dox-inducible *Tug1* expression in a *Tug1*-knockout background. (IV) Collection of testes from WT (*Tug1*^+/+^) (*n* = 4), KO (*Tug1*^*−/−*^) (*n* = 4), and *Tug1*^rescue^ (*n* = 3) mice for RNA sequencing. **e** *Tug1* gene expression level (log_2_TPM+1) in the testes of wild-type (gray), KO (red), and doxycycline-induced *Tug1*^rescue^ (blue) mice. Error bars represent the standard error of the mean. **f** Expression levels (log_2_TPM+1) for *Tug1* neighboring genes in WT (gray), KO (red), and *Tug1*^rescue^ (blue) mice. **g** Heatmap of fold changes of gene expression (TPM) for the reciprocally regulated genes in the comparisons of KO/WT and rescue/KO. **h** Examples of differentially expressed genes in the testes showing significant reciprocal regulation in WT (gray), KO (red), and *Tug1*^rescue^ (blue) mice

To gain further insight into the molecular roles of *Tug1*, we took a gene expression approach. We analyzed RNA-seq data from WT and *Tug1*^−/−^ tissues (testes, prostate, spleen, eyes, liver, heart, brain, and MEFs) and identified significant changes in the gene expression relative to wild type. Deletion of the *Tug1* locus was accompanied by 2139 significantly dysregulated genes across all samples examined. We observed global changes in the gene expression clustered by tissue type, indicating tissue-specific gene dysregulation (Fig. 4a; Additional file 6: Table S2; Additional file 7: Table S3; Additional file 10: Fig. S5). We found that while most dysregulated genes (~ 89%) were perturbed in only a single tissue (Fig. 4b), several genes were commonly dysregulated across multiple tissues (Fig. 4b; Additional file 6: Table S2; Additional file 7: Table S3). We then performed a gene set enrichment analysis (GSEA) using the differentially expressed genes for each tissue and observed enrichment of several pathways that were shared across the individual tissues. For example, oxidative phosphorylation, Myc targets, and epithelial to mesenchymal transition were found enriched in 7 of the 8 *Tug1*^−/−^ tissues (Fig. 4c).

Previous studies have suggested a role for *Tug1* RNA acting in *trans* on chromatin regulation and gene expression [22, 26, 28, 36, 43, 44]. Thus, we wanted to further investigate a *trans* role of *Tug1* RNA on the gene expression in the testes. We reasoned that overexpressing *Tug1* RNA in the *Tug1*-knockout background would enable to test if *Tug1* RNA alone could rescue genes found dysregulated in *Tug1*^−/−^ testes (Fig. 4d). Given that *Tug1* harbors a conserved ORF in the 5′ region (discussed in the next section), we identified an isoform of *Tug1* that lacks the 5′ region, thus ensuring we would address the role of *Tug1* RNA alone. To this end, we generated a doxycycline (dox)-inducible *Tug1* transgenic mouse by cloning a *Tug1* isoform downstream of a tet-responsive element (henceforth called tg(*Tug1*)) (Fig. 4d). Next, we generated compound transgenic mice that contained tg(*Tug1*) in the *Tug1*^−/−^ background that also contained an allele that constitutively expresses the reverse tetracycline transcriptional activator gene (CAG-rtTA3) (combined alleles henceforth called *Tug1*^rescue^) (Fig. 4d). This approach enabled systemic induction of *Tug1* RNA in the presence of dox, enabling to test if *Tug1* RNA expression alone would be sufficient to rescue gene expression in the testes and the male sterility phenotypes in *Tug1*^−/−^ mice.

To this end, we isolated the testes from wild-type, *Tug1*^−/−^, and dox-fed *Tug1*^rescue^ mice and performed RNA-seq. Because *Tug1*^rescue^ mice do not have endogenous *Tug1*, we were able to assess the levels of *Tug1* transgene RNA. RNA-seq and qRT-PCR indicated that the expression of transgenic *Tug1* RNA was significantly lower than wild type in the testes (Fig. 4e; Additional file 6: Table S2; Additional file 11: Fig. S6A). Furthermore, we used fluorescence-activated cell sorting (FACS) and isolated peripheral blood cell types (CD4, CD8, and NK) from wild-type, *Tug1*^+/−^, *Tug1*^−/−^, and dox-fed *Tug1*^rescue^ mice. qRT-PCR also showed lower levels of *Tug1* RNA induction relative to wild type, but *Tug1* transgene expression in peripheral blood cell types was generally higher than in the testes (Additional file 11: Fig. S6A-B). Even though the transgene expression was low in the testes, we reasoned that this would still be a valuable in vivo model to test *Tug1* RNA-mediated effects on gene regulation. Thus, we tested whether genes found dysregulated in the testes from *Tug1*^−/−^ mice could be rescued by ectopic expression of the *Tug1* transgene RNA in the *Tug1*^rescue^ model (Additional file 12: Fig. S7). We found that there was significant overlap of dysregulated genes between *Tug1*^−/−^ and *Tug1*^rescue^ testes (*p* value < 2.23–16, Fisher exact test). Notably, 53 (including *Tug1*) of the 1051 genes that were significantly dysregulated in *Tug1*^−/−^ testes were found significantly and reciprocally regulated in the testes from dox-fed *Tug1*^rescue^ mice (Fig. 4g, Table 1, and Additional file 6: Table S2). For example, a mitochondrial-related gene, *Mrarp*, and an aquaporin gene, *Aqp2*, are significantly upregulated in *Tug1*^−/−^ testes, but their expression was reduced to wild-type levels in the testes from dox-fed *Tug1*^rescue^ mice (Fig. 4h). Conversely, the predicted lncRNA gene *Gm28181* that is significantly reduced in *Tug1*^−/−^ testes is significantly upregulated to wild-type levels in the testes from dox-fed *Tug1*^rescue^ mice (Fig. 4h). While we observed a *trans*-effect for *Tug1* RNA, we did not observe any changes in the expression for the neighboring genes near the *Tug1* locus (Fig. 4f; Additional file 6: Table S2). Taken together, these data demonstrate that the *Tug1* lncRNA regulates a subset of genes by a *trans*-RNA-based mechanism, evident even at low levels of *Tug1* RNA.

**Table 1:** Genes reciprocally regulated by *Tug1* lncRNA in the testes

Gene name	Location	Mean TPM			Significance		Biological process
		WT	KO	Rescue	WT-KO	KO-Rescue	
*Pard3b*	chr1	2.66	2.13	2.74	***	***	Microtubule cytoskeleton organization
*Nav1*	chr1	1.40	2.33	1.45	**	**	Microtubule bundle formation
*Gm28181*	chr1	10.81	4.63	9.39	***	**	Unknown
*Col5a1*	chr2	7.16	8.97	7.85	*	*	Cell adhesion
*Hoxd10*	chr2	2.17	4.43	2.23	*	*	Regulation of transcription
*Sulf2*	chr2	12.20	16.74	13.21	***	**	Metabolic process
*Amy1*	chr3	16.43	23.06	14.58	*	***	Metabolic process
*Dhcr24*	chr4	30.92	43.38	34.72	***	*	Lipid metabolic process
*Tmem176a*	chr6	17.89	25.23	16.32	**	***	Lipid metabolic process
*Nat8f5*	chr6	2.30	4.78	2.13	**	**	System development
*Nat8*	chr6	9.57	16.53	8.23	**	***	Glutathione metabolic process
*Apoc1*	chr7	35.49	56.71	37.27	***	**	Lipid metabolic process
*Vmn1r181*	chr7	2.65	4.26	1.88	**	***	Unknown
*Klk1b27*	chr7	12.21	31.01	2.65	***	***	Proteolysis
*Klk1b21*	chr7	23.81	56.40	5.45	***	***	Proteolysis
*Klk1b24*	chr7	19.20	34.33	5.95	*	***	Proteolysis
*Cd209f*	chr8	1.57	2.64	1.23	*	***	Cell adhesion
*Gpt2*	chr8	25.46	33.70	24.06	**	***	Biosynthetic process
*Tug1*	chr11	40.08	0.89	1.60	***	***	–
*Spns2*	chr11	6.30	7.92	6.47	*	*	Sphingolipid metabolic process
*Serpina3n*	chr12	4.10	13.85	7.18	***	***	Inflammatory response
*Ankrd9*	chr12	43.02	50.58	44.74	*	*	Post-translational protein modification
*Sv2c*	chr13	5.59	7.53	5.15	***	***	Transmembrane transport
*3110070M22Rik*	chr13	110.87	93.69	120.25	**	*	Unknown
*Tmem267*	chr13	87.89	77.92	92.25	***	***	Unknown
*Il17rb*	chr14	4.20	6.08	3.65	***	***	Regulation of cell growth
*Stab1*	chr14	3.99	5.50	4.04	**	*	Cell adhesion
*Selenop*	chr15	88.40	130.97	85.90	***	***	Selenium compound metabolic process
*C7*	chr15	94.22	128.27	91.48	**	***	Immune response
*Aqp2*	chr15	4.93	13.17	6.48	***	***	Water transport
*AU021092*	chr16	30.38	44.08	33.37	***	*	Unknown
*Nrros*	chr16	3.00	3.93	2.99	*	*	Superoxide metabolic process
*Mrap*	chr16	4.83	8.21	4.75	***	***	Protein localization to plasma membrane
*Cyp21a1*	chr17	1.99	3.12	1.22	*	***	Steroid metabolic process
*Ston1*	chr17	28.51	34.85	29.11	***	***	Regulation of endocytosis
*Stk32a*	chr18	1.22	2.78	1.50	***	***	Protein phosphorylation
*mt-Rnr1*	chrM	211.26	253.11	219.24	*	*	Ribosome biogenesis

List of genes with TPM ≥ 1 and significant changes in the expression between wild-type (WT), *Tug1*^−/−^ (KO), and *Tug1*^rescue^ testes. Chromosomal location of the genes, mean TPM for each condition, and the main biological processes associated with each gene are listed. Significance of the fold change between wild-type versus *Tug1*^−/−^ (WT-KO) and *Tug1*^−/−^ versus *Tug1*^rescue^ (KO-Rescue) is indicated by asterisks (*p < 0.05; **p < 0.01, ***p < 0.001)

Next, we asked if dox-fed *Tug1*^rescue^ male mice had restored fertility and normal sperm production and morphology. Matings between dox-fed *Tug1*^rescue^ male mice (*n* = 3) and C57BL6/J female mice (*n* = 12) did not produce any progeny (Additional file 11: Fig. S6C). Moreover, we found that dox-fed *Tug1*^rescue^ males had a low sperm count (mean = 3.20 × 10^5^ ± 8.0 × 10^3^ cells/mL), similar to the levels observed in *Tug1*^−/−^ males (mean = 4.69 × 10^5^ ± 1.6 × 10^4^ cells/mL) compared to wild type (mean = 9.32 × 10^5^ ± 3.9 × 10^3^ cells/mL) (Additional file 11: Fig. S6D). These results were also confirmed with histological sectioning of the testes (Additional file 11: Fig. S6E). Further, we observed that dox-fed *Tug1*^rescue^ mice had a low proportion of normal shaped sperm, which was similar to the sperm observed in *Tug1*^−/−^ mice (Additional file 11: Fig. S6F). While this finding may suggest that the sterility phenotype is not due to the lncRNA transcript, we note that the lack of a fertility rescue may also be due to the low levels of *Tug1* expression from the transgene in the testes or because a different *Tug1* RNA isoform is required.

### The *Tug1* locus contains an evolutionary conserved ORF in humans and mice

**Fig. 5:**
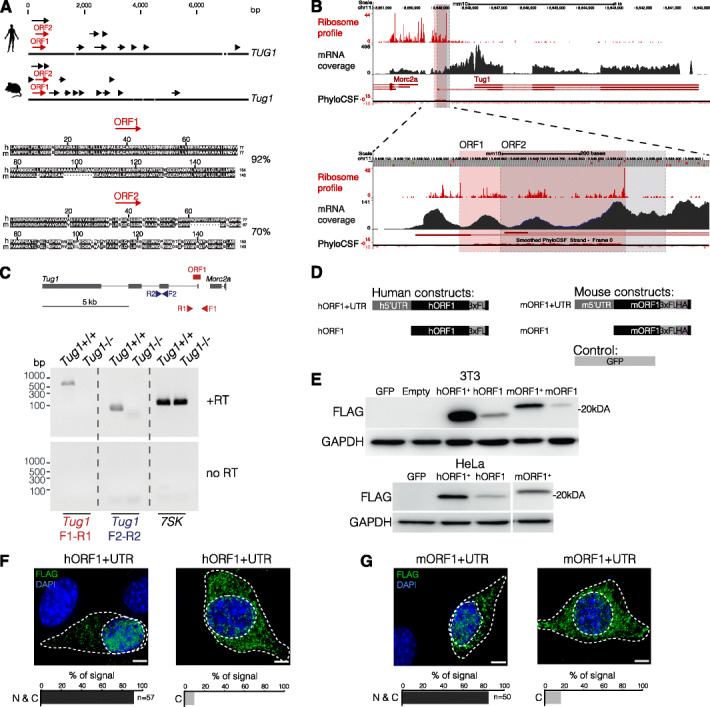
The 5′ region of *Tug1* encodes a conserved protein. **a** Open reading frame (ORF) search in human and mouse *Tug1* reveals multiple ORFs (arrows). ORF1 and ORF2 (red arrows) indicate two ORFs with greater than 70% amino acid conservation between humans and mice (92% and 70%, respectively). **b** *Tug1* mouse genomic locus (mm10) is shown. Ribosome occupancy (ribosome profile), RNA-seq (mRNA coverage), and evolutionary protein-coding potential (PhyloCSF) across the *Tug1* locus in mouse embryonic fibroblasts (MEFs) are depicted. ORF1 and ORF2 are outlined with red and gray boxes, respectively (top). Tracks surrounding both ORFs are zoomed in for clarity (bottom). **c** ORF RNA in wild-type and *Tug1*^−/−^ testes for ORF1. Top: scheme of *Tug1* locus showing the location of primers (triangles) and ORF1 (red rectangle). Bottom: RT-PCR of *Tug1*, *Tug1*-ORF1, and the endogenous control 7SK. **d** Scheme of human and mouse ORF1 construct design. A 3xFLAG epitope tag was inserted prior to the stop codon of ORF1. Mouse constructs were dual-tagged with both 3xFLAG and HA tags. Expression constructs were designed with (hORF1+UTR, mORF1+UTR) and without (hORF1, mORF1) the 5′ UTR. Constructs and GFP as control were inserted into pcDNA3.1(+). Forty-eight hours post-transfection, 3T3 and HeLa cells were harvested for western blot (WB) (shown in **e**) or analyzed by immunofluorescence (IF) (shown in **f**, **g**). **e** Western blot targeting the 3xFlag (FLAG) in 3T3 and HeLa cells expressing human and mouse constructs, respectively. GAPDH was used as a loading control. **f**, **g** Maximum intensity projections of 3T3 cells expressing human and mouse constructs. Immunostaining against the Flag tag (green) and DAPI (blue) is shown. The bar plot shows the localization analysis of human and mouse TUG1-BOAT. N & C indicates nuclear and cytoplasmic localization, and C indicates only cytoplasmic. Scale bar is 5 μm

A number of studies have reported that some lncRNA loci can also harbor ORFs that can produce functional proteins [45]. Because PhyloCSF revealed a region with high coding potential in the human and mouse *TUG1*/*Tug1* locus (Fig. 1a; Additional file 13: Fig. S8A), we wanted to further explore this possibility using computational, biochemical, and cell-based assays. We systematically screened for ORFs that displayed strong conservation across species, allowing for both canonical (AUG) and non-canonical (CUG and UUG) translation start codons, a feature that has been previously identified at lncRNA loci [13, 46]. We identified multiple short ORFs in the human and mouse *TUG1*/*Tug1* locus (11 and 15, respectively) (Fig. 5a), which is consistent with previous studies [22, 47, 48]. Two ORFs (designated as ORF1 and ORF2) at the 5′ region of *TUG1*/*Tug1* drew our attention due to their conserved translational start and stop sites, as well as their high level of nucleotide conservation between humans and mice (Fig. 5a). ORF1 (154 amino acids in humans) and ORF2 (153 amino acids in humans) both start with a non-canonical start codon (CUG) and share 92% and 70% cross-species identity at the amino acid level, respectively. Notably, ORF1 has a high PhyloCSF score (350) and shows conservation spanning its entire sequence, whereas ORF2 does not show patterns of preserving synonymous mutations past the ORF1 stop codon (Fig. 5b; Additional file 13: Fig. S8A).

To further characterize the potential translated regions in *Tug1*, we analyzed publicly available ribosome profiling data [49], a technique used to identify regions where RNAs are bound to ribosomes by high-throughput sequencing [13, 50–52]. We found pronounced ribosomal occupancy across the entire *Tug1* ORF1 sequence with a sharp decrease at its stop codon (Fig. 5b). A similar pattern of *Tug1* ORF1 is also observed from ribosome profiling in human, mouse, and rat heart tissue (Additional file 13: Fig. S8) [47], whereas ORF2 does not show ribosome occupancy above the background level after the ORF1 stop codon (Fig. 5b, Additional file 13: Fig. S8A). To determine if a transcript containing ORF1 (located in the 5′ region) is expressed in the testes, we performed reverse transcriptase PCR (RT-PCR) on wild-type and *Tug1*^−/−^ testes. Using a primer set that spans ORF1, we detected a band at approximately 700 bp in the wild-type sample in which the RT enzyme was added and did not detect a product in either the wild-type sample with no RT enzyme added or in the *Tug1*^−/−^ control samples (Fig. 5c). As a loading control, *7SK*, an abundant snRNA [53, 54], is detected in both wild-type and *Tug*1^−/−^ samples (Fig. 5c). Taken together, these results provide evidence that the 5′ region containing ORF1 is expressed in the testes and presents the possibility that this ORF could generate the predicted protein. Thus, we designated the putative protein originating from ORF1 as TUG1-BOAT (*Tug1*-Bifunctional ORF and Transcript).

Next, to determine if TUG1-BOAT can produce a stable protein, we generated C-terminal epitope-tagged human and mouse TUG1-BOAT expression constructs with and without the 5′ leader sequences (Fig. 5d). As a negative control, we generated a construct containing GFP in place of the TUG1-BOAT cDNA sequence. We then transfected 3T3 (mouse) and HeLa (human) cells and tested for TUG1-BOAT translation by western blot analysis. We detected bands at approximately 19 kDA and 21 kDa in both cell lines (Fig. 5e), which closely corresponds to the predicted molecular weights of hTUG1-BOAT (18.7 kDA) and mTUG1-BOAT (19 kDa) fusion constructs, respectively. Furthermore, the presence of the 5′ UTR notably enhances the translation of human and mouse ORF1. Having detected a protein of the expected size from human and mouse TUG1-BOAT constructs, we next investigated the protein’s subcellular localization by immunofluorescence. We observed that human and mouse TUG1-BOAT is distributed throughout the nucleus and cytoplasm in the majority of the cells (> 80% cells, *n* = 50) (Fig. 5f, g). However, in a subset of cells, TUG1-BOAT was predominantly cytoplasmic (< 20% of cells, *n* = 50) (Fig. 5f, g). We further found that TUG1-BOAT co-localizes with the mitochondria in an overexpression context (Additional file 13: Fig. S8C). Collectively, these results show that ORF1, with its 5′ UTR and native non-canonical translational start site, can be translated into a stable protein in both human and mouse cells.

### TUG1-BOAT overexpression compromises mitochondrial membrane potential

**Fig. 6:**
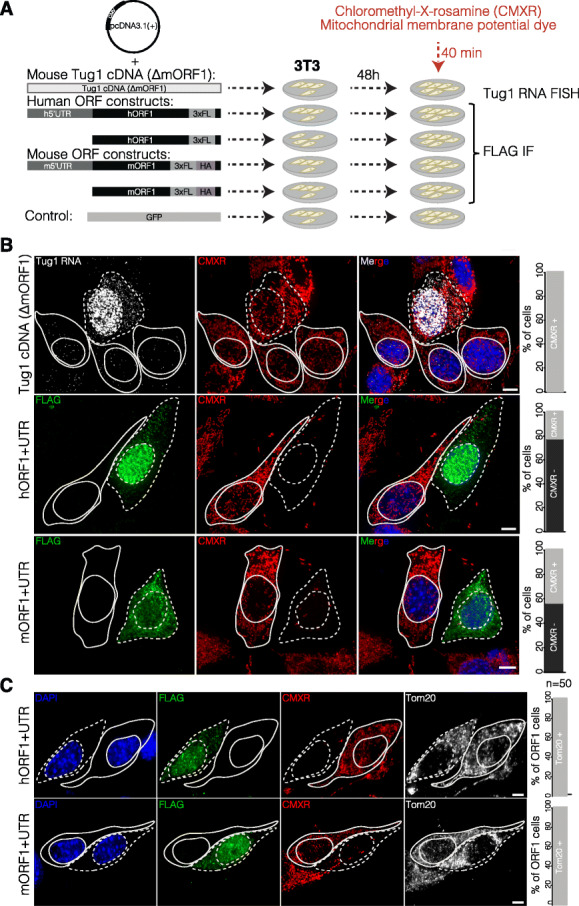
Overexpression of TUG1-BOAT compromises mitochondrial membrane potential. **a** Construct and transfection scheme. Human and mouse ORF1, and mouse *Tug1* cDNA lacking the ORF1 region (*Tug1* cDNA ∆mORF1) were inserted into pcDNA3.1(+). Chloromethyl-X-rosamine (CMXR) was added to visualize the mitochondria 48 h post-transfection. After staining, cells were fixed and processed for anti-FLAG immunofluorescence (IF) or *Tug1* RNA FISH. **b** Maximum intensity projections of z-stacks acquired 48 h post-transfection of 3T3 cells with indicated plasmids and staining with CMXR. *Tug1* RNA overexpression was monitored by *Tug1* single-molecule RNA FISH (gray) and TUG1-BOAT by immunostaining against the FLAG tag (green). CMXR is shown in red and DAPI in blue. On the right, quantification of cells positive for *Tug1* RNA or TUG1-BOAT and mitochondria by CMXR (*n* = 50). Scale bar is 5 μm. **c** Maximum intensity projections of z-stacks acquired 48 h post-transfection of 3T3 cells with the indicated plasmids, stained with CMXR (red) and immunostained against mitochondrial membrane translocase TOM20 (gray). On the right, quantification of cells overexpressing TUG1-BOAT and lacking CMXR staining showing an intact mitochondrial membrane assessed by TOM20. The nuclei were stained with DAPI (blue). Scale bar is 5 μm

Given the localization of overexpressed TUG1-BOAT to the mitochondria and that oxidative phosphorylation was one of the most affected pathways across multiple *Tug1*^−/−^ tissues (Fig. 4c), we hypothesized that TUG1-BOAT may have a role in the mitochondria. To this end, we first examined the mitochondrial membrane potential by using chloromethyl-X-rosamine (CMXR), a lipophilic fluorescent cation that accumulates in the negatively charged interior of mitochondria [55]. We overexpressed human and mouse TUG1-BOAT with and without the 5′ UTR in 3T3 cells, along with the controls, a GFP-containing plasmid, and a *Tug1* construct lacking ORF1 (*Tug1* cDNA ∆mORF1) (Fig. 6a). Notably, cells with either human or mouse TUG1-BOAT showed a reduction in mitochondrial staining by CMXR (22% and 44% CMXR stained cells, respectively), compared to cells in the same culture not expressing TUG1-BOAT (Fig. 6b). In contrast, cells expressing GFP or *Tug1* cDNA ∆mORF1 were positive for CMXR staining in all cells examined, thus indicating that CMXR staining deficiency is induced by overexpression of the TUG1-BOAT protein alone, rather than the *Tug*1 RNA (Fig. 6b; Additional file 14: Fig. S9).

Since CMXR is commonly used to measure mitochondrial membrane potential, we reasoned that either impaired mitochondrial integrity or impaired redox potential at the mitochondrial membrane could account for the accumulation defect of CMXR in mitochondria upon TUG1-BOAT overexpression. To address these possibilities, we immunostained for TOM20, a redox independent translocase located on the outer mitochondrial membrane [56]. We observed staining for TOM20 in cells without CMXR staining, indicating that mitochondria were intact in cells overexpressing human or mouse TUG1-BOAT (Fig. 6c). Taken together, these results suggest that ectopic levels of TUG1-BOAT protein alter mitochondrial membrane potential.

## Discussion

**Fig. 7:**
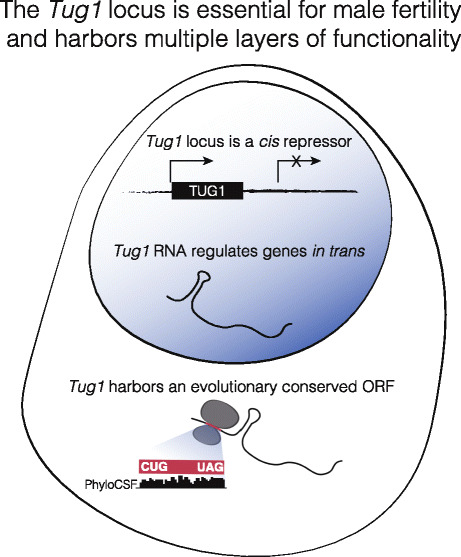
Model of molecular modalities at *Tug1* locus. Scheme showing two noncoding activities and one potential coding activity at the *Tug1* locus. *Tug1* locus harbors a *cis*-repressive element that suppresses the expression of downstream genes across multiple different tissue types. *Tug1* lncRNA regulates a subset of genes in *trans* via an RNA-based mechanism. Lastly, the 5′ region of *Tug1* harbors and evolutionary conserved ORF with a non-canonical start codon and high ribosome profiling coverage

Noncoding RNAs play roles in diverse biological functions, and their loci can possibly harbor multiple molecular modalities (DNA, RNA, the act of transcription, and misannotated proteins), each with the potential to exert function. In this study, we report an essential role in male fertility for the *Tug1* lncRNA locus in vivo and also report an underappreciated molecular complexity at the *Tug1* locus (Fig. 7). We find evidence that the *Tug1* locus harbors a (i) *cis*-acting DNA repressive element, (ii) a lncRNA that acts on gene expression in *trans*, and (iii) a conserved putative ORF which when overexpressed affects mitochondrial membrane potential (Fig. 7). Together, our results have several important biological implications.

### LncRNAs in male fertility

First, our study indicates that the *Tug1* locus has an essential role in male fertility in mice. Based on our results, we speculate that the sterility of *Tug1*^−/−^ males arises from a combination of oligozoospermia (low sperm count) and teratozoospermia (abnormal morphology). Notably, there is some evidence that *Tug1*/*TUG1* may have a conserved role in male fertility in humans. Microarray profiling of sperm from sterile men (a condition called teratozoospermia) has significantly less *TUG1* expression compared with sperm from fertile males (Additional file 15: Fig. S10) [57]. Thus, we speculate that the *Tug1*^−/−^ mouse model used in this study could potentially be of value to further investigate the functions of *Tug1*/*TUG1* with relevance to human male fertility.

Indeed, studies have performed systematic gene expression profiling at defined stages during spermatogenesis and have identified many developmentally regulated lncRNAs, suggesting that lncRNAs may have important roles during spermatogenesis [58]. Four other lncRNAs (*Tslrn1*, *Tsx*, *Pldi*, and *Mrlh*) have been found to be important in spermatogenesis, but in contrast to the *Tug1*-knockout phenotype reported in this study, none lead to male sterility when disrupted in mouse models [58–61]. On this note, identifying overt functions of lncRNA loci with respect to embryogenesis, viability, and fertility has been a challenge. A recent study that generated 32 deletion alleles for 25 lncRNA loci in zebrafish reported one mutant with abnormal development [62]. Thus, overt functions for individual lncRNA loci, such as the one observed for *Tug1*, may be less common.

### Multiple molecular modalities of the Tug1 locus

Second, we found that the *Tug1* locus harbors at least two distinct noncoding activities, and potentially a third coding one. Several lines of evidence indicate that the *Tug1* locus has a *cis*-acting repressive DNA function. We observed that upon deletion of the *Tug1* locus, several genes located downstream of *Tug1* were consistently upregulated across multiple tissues, and in an allele-specific manner in the testes. This dysregulation is consistent with a previous study from our group in which genes located near the *Tug1* locus were dysregulated in the brain of *Tug1*^−/−^ mice [41]. Collectively these data suggest that the local repressive *cis*-effect is mediated by DNA regulatory elements within the *Tug1* locus. Consistent with a repressive modality of the *Tug1* locus, a recent study which systematically tested for enhancer activity across lncRNA loci, reported a lack of enhancer activity across the *Tug1* gene body but found enhancer activity at all other lncRNA loci examined [11]. Contrary to enhancers, only a handful of repressor elements and silencers have been identified and characterized in mammalian genomes [63–66]. Further defining the precise DNA repressive elements as well as their mechanism will be of interest to understand the regulatory principles of DNA elements within the *Tug1* locus.

In addition to a DNA regulatory modality, this study finds evidence for an RNA-based role for *Tug1* in the testes by a *trans* mechanism. In support of this conclusion, we found that a subset of genes dysregulated in *Tug1*^−/−^ testes could be rescued by ectopic expression of *Tug1* RNA, even at low levels. While the *Tug1* transgene was expressed at lower levels than wild-type *Tug1* RNA, other lncRNAs such as *Hottip* and *Xist* have been shown to exert a biological activity at relatively low copy numbers [67, 68]; thus, *Tug1* RNA appears to have functional activity even at low levels. Consistent with our finding of a *trans*-acting role for *Tug1* RNA on gene expression, a previous study found that *Tug1* RNA can regulate the levels of *Ppargc1a* mRNA in cultured podocytes [26]. In our RNA-seq dataset of 8 different tissues, *Ppargc1a* was not significantly dysregulated, but it is important to note that our dataset does not include the kidney. We also note that low levels of *Tug1* transgene RNA were not sufficient to rescue the *Tug1* male fertility defect. More studies will be needed to determine the direct or indirect mechanisms of *Tug1* RNA on gene expression in a male germ cell context in vivo.

A number of recent studies have identified ORFs at lncRNA loci and have also demonstrated that some ORFs can produce proteins [13, 15–18, 46, 50, 51]. Thus, we also explored the possibility of a protein modality embedded at the *Tug1* locus. We demonstrate that the 5′ region of the *Tug1* locus contains an evolutionarily conserved ORF that when overexpressed, impacts mitochondrial membrane potential. The protein product which we named TUG1-BOAT is predicted to have a high positive charge (net charge ~ + 16.5). Thus, we speculate that the accumulation of such a positively charged protein at the mitochondria in an overexpression context could lead to depolarization of the mitochondrial membrane. Consistent with this finding, a recent study also identified a conserved ORF in the 5′ region of the human *TUG1* locus and showed that the ORF produces a stable protein that localizes to the mitochondria [47]. In addition, the evidence for a mitochondrial role for *Tug1* does not appear to be limited to the putative protein. One study found that overexpression of a *Tug1* RNA isoform lacking ORF1 affects mitochondrial bioenergetics in cultured podocytes derived from a diabetic nephropathy mouse model [26]. In our study, we did not observe any effect of *Tug1* RNA (lacking the ORF1 sequence) overexpression on mitochondrial membrane potential by CMXR staining in cell-based assays. Identifying endogenous and disease contexts in which *Tug1* RNA and TUG1-BOAT are detected and function endogenously will be important to further explore the potential link between *Tug1* and the mitochondria and determine whether the locus does contain a third coding function.

### The Tug1 locus has multiple regulatory modalities with potential function in spermatogenesis

Finally, this study opens the possibility that *Tug1* DNA, RNA, or putative protein could individually or in combination mediate the observed male fertility defect in *Tug1*-knockout mice. There are few biological contexts in the literature describing noncoding loci implicated in human disease with potential DNA, RNA, and protein functionalities. However, one intriguing example is the human *D4Z4* locus, which is a repeat region that is reduced in copy number in humans with facioscapulohumeral dystrophy (FSHD) [69, 70]. Interestingly, in the disease context where the repeat number is reduced, local repressive chromatin is lost leading to transcriptional upregulation of proximally located genes [71, 72]. Mechanistic studies spanning decades have identified a number of candidate molecular modalities at the *D4Z4* locus, including a lncRNA, a protein that is detected in the disease context, and a DNA repressor element [71–76]. While our study does not resolve the molecular modes of action mediating the male fertility defect in *Tug1*-knockout mice, there is evidence to support a potential role in male fertility for each modality, thus warranting further investigation.

There is some evidence that the *cis* genes dysregulated upon deletion of the *Tug1* locus have a role in male fertility. Loss-of-function mutations in 2 of the 6 *cis* genes upregulated in *Tug1*^−/−^ testes, *Smtn* and *Pla2g3*, are characterized to have male fertility and sperm maturation defects [77, 78]. However, the impact of these genes on male fertility in an overexpression context has not been reported. A role in male fertility for the other four upregulated *cis* genes upon deletion of *Tug1* (*Gm11946*, *Rnf185*, *Selm*, and *8430429K09Rik*) have not yet been reported. In addition, there is some evidence for a potential role of *Tug1* RNA in male fertility. Mice with a loss-of-function mutation for *Selenop*, a gene that was found significantly upregulated in *Tug1*^−/−^ testes and significantly and reciprocally regulated in *Tug1*^*rescue*^ testes, are reported to have reduced male fertility [79]. As for a role for the putative protein, TUG1-BOAT, there is currently limited evidence for its endogenous existence. Thus, future work clarifying an endogenous presence and function for TUG1-BOAT will be necessary to understand whether it contributes to the male fertility defect.

## Conclusions

In summary, this study provides genetic evidence for an essential role of the *Tug1* locus in male fertility. This study also highlights the molecular complexity embedded at lncRNA loci as our findings provide evidence that the *Tug1* locus harbors two distinct noncoding activities: (i) a *cis* DNA repressor and (ii) a *trans*-acting lncRNA, and we find a potential third protein-coding activity. Therefore, going forward, it will be important to investigate the individual and/or combined roles of *Tug1* DNA, RNA, and/or putative protein in male fertility as well as in disease contexts in which *Tug1* is altered.

## Methods

### Mice and ethics statement

*Tug1*^*tm1.1Vlcg*^-knockout mice have been described previously [20, 41]. To remove the *loxP*-flanked neomycin resistance gene included in the targeting construct, we crossed *Tug* ^*tm1.1Vlcg*^ mice to *C57BL6/J* mice and then to a cre-recombinase strain (*B6.C-Tg*^*(CMV-cre)1Cgn/J*^, The Jackson Laboratory, 006054). Mice free of both the neomycin-resistance and cre-recombinase genes were selected for colony expansion and subsequently backcrossed to *C57BL/6J* mice. The *Tug1*-knockout allele was maintained by heterozygous breeding, and mutant mice were identified by genotyping for loss of the *Tug1* allele and gain of the *lacZ* cassette (Transnetyx, Inc.).

For allele-specific gene expression analyses, we generated *Tug1*^BL6-KO/Cast-WT^ mice by crossing inbred *Mus castaneus* (Cast/EiJ) males (The Jackson Laboratory, 000928) with inbred heterozygote *Tug1* females. The F1 hybrid male progeny (three wild-type *Tug1*^BL6-WT/Cast-WT^ and four with a maternal *Tug1*-knockout allele *Tug1*^BL6-KO/Cast-WT^) were used for allele-specific expression studies.

To generate an inducible *Tug1*-overexpression mouse, tg(*Tug1*), we cloned *Tug1* cDNA (see the “Sequences and primers” section) into a Tet-On vector (pTRE2). Full-length *Tug1* (Ensembl ID: ENSMUST00000153313.2) was amplified from Riken cDNA clone E330021M17 (Source Bioscience) using specific primers containing MluI and EcoRV restriction sites (see the “Sequences and primers” section). After gel purification, we sub-cloned the amplicon using the MluI and EcoRV restriction sites into a modified Tet-On pTRE2pur vector (Clontech 631013) in which the bGlobin-intron was removed. We verified the absence of mutations from the cloned *Tug1* cDNA by sequencing (see the “Sequences and primers” section). We injected this cassette into the pronucleus of C57BL/6J zygotes (Beth Israel Deaconess Medical Center Transgenic Core). Two male founder mice containing random integration of the tg(*Tug1*) cassette were identified by genotyping for the pTRE allele and individually mated to female C57BL/6J mice (The Jackson Laboratory, 000664) to expand the colonies. Next, we generated quadruple allele transgenic mice to test the functionality of the *Tug1* RNA by the following strategy. We mated tg(*Tug1*) males to *Tug1*^*tm1.1Vlcg*^ females and identified male progeny that were *Tug1*^+/−^; tg(*Tug1*). These mice were then mated to female *rtTA* mice (B6N.FVB(Cg)-Tg(CAG-rtTA3)4288Slowe/J mice (The Jackson Laboratory, 016532)), and we identified male progeny that were *Tug1*^+/−^; tg(*Tug1*), *rtTA*. Finally, we mated male *Tug1*^+/−^; tg(*Tug1*), *rtTA* mice to *Tug1*^+/−^ females, and at the plug date, females were put on 625 mg/kg doxycycline-containing food (Envigo, TD.01306). We genotyped progeny from the above matings (Transnetyx, Inc.) and identified male progeny that were *Tug1*^−/−^; tg(*Tug1*), *rtTA*, and maintained these mice on the doxycycline diet until the experimental end point.

### Cell lines and cell culture

We derived primary wild-type and *Tug1*^*−/−*^ mouse embryonic fibroblasts (MEFs) from E14.5 littermates from timed *Tug1*^*+/−*^ intercrosses as described [80]. We maintained MEFs as primary cultures in DMEM, 15% FBS, pen/strep, l-glutamine, and non-essential amino acids. We genotyped MEFs derived from each embryo and used only male *Tug1*^*−/−*^ and wild-type littermate MEFs at passage 2 for all experiments. 3T3 (ATCC, CRL-1658), HeLa (ATCC, CRM-CCL-2), and BJ (ATCC, CRL-2522) cell lines were purchased from ATCC and cultured as recommended.

### Whole-mount in situ hybridization

We generated an antisense riboprobe against *Tug1* (see the “Sequences and primers” section) from plasmids containing full-length *Tug1* cDNA (Ensembl id: ENSMUST00000153313.2) and performed in situ hybridization on a minimum of three *C57BL6/J* embryos per embryonic stage. For whole-mount staining, embryos were fixed in 4% paraformaldehyde for 18 h at 4 °C, followed by three washes in PBS for 10 min. We then dehydrated embryos through a graded series of 25%, 50%, and 75% methanol/0.85% NaCl incubations and then finally stored embryos in 100% methanol at − 20 °C. Embryos were then rehydrated through a graded series of 75%, 50%, 25%, and methanol/0.85% NaCl incubations and washed twice with PBS with 0.1% Tween-20 (PBST). Embryos were treated with 10 mg/mL proteinase K in PBST for 10 min (E8.0, E9.5) or 30 min (E10.5, E11.5, and E12.5). Samples were fixed in 4% paraformaldehyde/0.2% glutaraldehyde in PBST for 20 min at room temperature and washed twice with PBST. We then incubated samples in pre-hybridization solution for 1 h at 68 °C and then incubated samples in 500 ng/mL of *Tug1* antisense or sense riboprobe at 68 °C for 16 h. Post-hybridization, samples were washed in stringency washes and incubated in 100 μg/mL RNaseA at 37 °C for 1 h. Samples were washed in 1× maleic acid buffer with 0.1% Tween-20 (MBST) and then incubated in Roche Blocking Reagent (Roche, #1096176) with 10% heat-inactivated sheep serum (Sigma, S2263) for 4 h at room temperature. An anti-digoxigenin antibody (Roche, 11093274910) was used at 1:5000 and incubated for 18 h at 4 °C. Samples were washed 8 times with MBST for 15 min, 5 times in MBST for 1 h, and then once in MBST for 16 h at 4 °C. Prior to developing, the samples were washed three times with NTMT (100 mM NaCl, 100 mM Tris-HCl (pH 9.5), 50 mM MgCl2, 0.1% Tween-20, 2 mM levamisole). The in situ hybridization signal was developed by adding BM Purple (Roche, 11442074001) for 4, 6, 8, and 12 h. After the colorimetric development, samples were fixed in 4% paraformaldehyde and cleared through a graded series of glycerol/1× PBS and stored in 80% glycerol. Imaging was performed on a Leica M216FA stereomicroscope (Leica Microsystems) equipped with a DFC300 FX digital imaging camera.

### *Tug1* single-molecule RNA FISH

We performed *Tug1* single-molecule RNA FISH as described previously [81]. Briefly, 48 oligonucleotides labeled with Quasar 570 and Quasar 670 tiled across human/mouse *Tug1* transcripts were designed with LGC Biosearch Technologies’ Stellaris probe designer (Stellaris Probe Designer version 4.2) and manufactured by LGC Biosearch Technologies.

Human foreskin fibroblasts (ATCC® CRL-2522™) and mouse 3T3 fibroblasts (ATCC, CRL-1658™) were seeded on glass coverslips previously coated with poly-l-lysine (10 μg/mL) diluted in PBS. Prior to hybridization, coverslips were washed twice with PBS, fixed with 3.7% formaldehyde in PBS for 10 min at room temperature, and washed twice more with PBS. Coverslips were immersed in ice-cold 70% EtOH and incubated at 4 °C for a minimum of 1 h. We then washed the coverslips with 2 mL of Wash Buffer A (LGC Biosearch Technologies) at room temperature for 5 min. Next, we hybridized cells with 80 μL hybridization buffer (LGC Biosearch Technologies) containing *Tug1* probes (1:100) overnight at 37 °C in a humid chamber. The following day, we washed the cells with 1 mL of Wash Buffer A for 30 min at 37 °C, followed by another wash with Wash Buffer A containing Hoechst DNA stain (1:1000, Thermo Fisher Scientific) for 30 min at 37 °C. Coverslips were washed with 1 mL of Wash Buffer B (LGC Biosearch Technologies) for 5 min at room temperature, mounted with ProlongGold (Life Technologies) on glass slides, and left to curate overnight at 4 °C before proceeding to image acquisition (see below).

### Sperm counts and morphology

*Tug1*^*−/−*^ (*n* = 8) and wild-type (*n* = 9) males between 8 and 41 weeks of age were sacrificed and weighed. We then dissected the entire male reproductive tract in phosphate-buffered saline (PBS). One testis was removed, weighed, and fixed in 4% paraformaldehyde (PFA) for histology (see below). Sperm were collected from one cauda epididymis by bisecting and suspending the tissue in a solution of Biggers-Whitten-Whittingham (BWW) sperm media at 37 °C. After a 15-min incubation, we used the collected sperm solutions to analyze sperm morphology and counts.

We characterized sperm morphology by fixing sperm in 2% PFA in PBS, mounting 20 μL of suspended sperm in Fluoromount-G media (Southern Biotech) on Superfrost glass slides (Thermo Fisher Scientific) and scanning each slide in a linear transect, recording the morphology as normal or abnormal for each sperm cell encountered (between 30 and 120 sperm). When abnormal, we also recorded the type of morphological defects: headless, head angle aberrant, head bent back to midpiece, debris on the head, debris on the hook, head misshapen, midpiece curled, midpiece kinked, midpiece stripped, debris on the midpiece, tailless, tail curled, tail kinked, broken tail, or multiple cells annealed together.

Sperm counts for each *Tug1*^*−/−*^ (*n* = 7) and wild-type (*n* = 9) mice were determined using a Countess Automated Cell Counter according to the manufacturer’s protocol (Life Technologies, Carlsbad, CA). For the *Tug1*^rescue^ experiment, sperm counts for control (WT and *Tug1*^+/−^) (*n* = 2), *Tug1*^−/−^ (*n* = 2), and *Tug1*^−/−^; tg(*Tug*1); *rtTA* mice (*n* = 3) were determined by manual counts using a hemocytometer. For all analyses, statistical comparisons between *Tug1*^*−/−*^ and wild type were performed using the two-tailed Wilcoxon rank-sum tests with an *a* = 0.05. The results for testis, sperm count, and morphological parameters are presented in Additional file 5: Table S1. All statistical comparisons of *Tug1*^*−/−*^ versus wild type for relative testis size, sperm morphology, and sperm counts were performed using R (Wilcoxon rank-sum test and principal component analysis (PCA)).

### *lacZ* and histological staining of male reproductive tissues

Expression of the knock-in *lacZ* reporter and histological staining for morphological analysis of male reproductive tissues was conducted on the testes and epididymis from *Tug1*^*−/−*^ (*n* = 2) and wild-type (*n* = 2) mice. We fixed the testis and epididymis in 4% paraformaldehyde in PBS overnight at 4 °C and washed the tissues three times in PBS. For *lacZ* staining, we rinsed *Tug1*^*+/−*^ and wild-type tissues three times at room temperature in PBS with 2 mM MgCl2, 0.01% deoxycholic acid, and 0.02% NP-40. We performed X-gal staining by incubating the tissues for up to 16 h at 37 °C in the same buffer supplemented with 5 mM potassium ferrocyanide and 1 mg/mL X-gal. The staining reaction was stopped by washing three times in PBS at room temperature, followed by 2 h post-fixation in 4% paraformaldehyde at 4 °C.

We then embedded the organs in paraffin, sectioned the organs at 6 μm thickness, and then mounted the sectioned samples onto glass microscope slides. The testis sections were additionally stained with Mayer’s hematoxylin, periodic acid, and Schiff’s reagent (VWR, 470302-348), and the epididymis sections were stained with eosin (VWR, 95057-848). Images were collected using a Zeiss AxioImager.A1 upright microscope or on an Axio Scan Z.1 (Zeiss).

### RNA isolation and RNA-seq library preparation

We isolated total RNA from mouse tissues, mouse embryonic fibroblasts (MEFs), and blood cells using TRIzol (Invitrogen, 15596026) by chloroform extraction followed by spin-column purification (RNeasy mini or micro kit, Qiagen) according to the manufacturer’s instructions. RNA concentration and purity were determined using a Nanodrop. We assessed RNA integrity on a Bioanalyzer (Agilent) using the RNA 6000 chip. High-quality RNA samples (RNA integrity number ≥ 8) were used for library preparation. We then constructed mRNA-seq libraries using the TruSeq RNA Sample Preparation Kit (Illumina) as previously described [82]. The libraries were prepared using 500 ng of total RNA as input and a 10-cycle PCR enrichment to minimize PCR artifacts. Prior to sequencing, we ran libraries on a Bioanalyzer DNA7500 chip to assess purity, fragment size, and concentration. Libraries free of adapter dimers and with a peak region area (220–500 bp) ≥ 80% of the total area were sequenced. We then sequenced individually barcoded samples in pools of 6, each pool including *Tug1* mutant and wild-type samples, on the Illumina HiSeq platform using the rapid-full flow cell with the 101-bp paired-end reads sequencing protocol (Bauer Core, Harvard University FAS Center for System Biology).

### RNA-seq and gene set enrichment analyses

We mapped sequencing reads to the reference mouse genome (GRCm38) by STAR [83] with the gene annotation obtained from GENCODE (vM16). We counted uniquely mapped reads for genes by featureCounts [84] and calculated transcripts per million (TPM) for genes to quantify the gene expression level after normalization of sequencing depth and gene length. Clustering of gene expression between tissues was done with Ward’s method using Jensen-Shannon divergence between tissues as the distance metric. The R package, Philentropy, was used for calculation of the Jensen-Shannon divergence [85].

We identified differentially expressed genes by comparing the mean read counts of biological replicates between the groups (wild-type vs. *Tug1*^-/-^ and  *Tug1*^-/-^ vs. *Tug1*^rescue^) using the generalized linear model. Statistical significance was calculated with the assumption of the negative binomial distribution of the read counts and with the empirical estimation of variance by using the R packages DESeq2 [86] and fdrtool [87]. The genes were filtered if their read counts were less than three in every biological replicate. The genes were called significant if their adjusted *p* values by the false discovery rate (FDR) method were smaller than 0.05.

We performed gene set enrichment analysis (GSEA) to evaluate the enrichment of the gene sets available from MSigDB [88] after mapping genes to gene sets by gene symbols. The statistical significance of a gene set was calculated with the test statistics of individual genes computed by DESeq2. If the FDR-adjusted *p* value is less than 0.1, the term was called significant. We performed the GSEA analysis using the R package, CAMERA [89].

### Allele-specific gene expression analysis

We performed allele-specific expression analysis as previously described [90]. For mouse testis samples, we created a *C57BL/6J*, *Cast/EiJ* diploid genome by incorporating single nucleotide polymorphisms and indels (obtained from the Mouse Genome Project: ftp://ftp-mouse.sanger.ac.uk/REL-1303-SNPs_Indels-GRCm38) from both strains into the *M. musculus* GRCm38 reference genome sequence. We created a transcriptome annotation set as follows. The gencode.vM2.annotation GTF file was downloaded, and Mt_rRNA, Mt_tRNA, miRNA, rRNA, snRNA, snoRNA, Mt_tRNA_pseudogene, tRNA_pseudogene, snoRNA_pseudogene, snRNA_pseudogene, scRNA_pseudogene, rRNA_pseudogene, and miRNA_pseudogene were removed (not enriched in our RNA-seq libraries). To create an extensive set of transcripts, we added to the gencode.vM2.annotation all transcripts from the UCSC knownGene mm10 annotation file, which are not represented in the gencode.vM2.annotation set. We also added all functional RNAs from the Functional RNA database (fRNAdb) [91], which did not intersect with any of the previously incorporated transcripts. From this, we then used the UCSC liftOver utility to generate a *C57BL/6J*, Cast/EiJ diploid transcriptome set.

Each RNA-seq library was first subjected to quality and adapter trimming using the Trim Galore utility (http://www.bioinformatics.babraham.ac.uk/projects/trim_galore) with stringency level 3. We then mapped each of the *C57BL/6J::Cast/EiJ* hybrid RNA-seq libraries to the *C57BL*/*6J* and *Cast*/*EiJ* diploid genome and transcriptome splice junctions using STAR RNA-seq aligner [83], allowing a maximum of 3 mismatches. The data were mapped twice, where after the first mapping step, we incorporated valid splice junctions that were reported by STAR to exist in the RNA-seq data. We then transformed the genomic alignments to transcriptomic alignments. Following that, we estimated the expression levels with their respective uncertainties for each transcript in our *C57BL/6J* and *Cast/EiJ* diploid transcriptome using MMSEQ [92]. The posterior FPKM samples were transformed into TPM units with a minimum expression TPM cutoff set to 0.01. In any RNA-seq sample, any transcript for which its MMSEQ posterior median TPM was lower than 0.01 was set to 0.01 (used as the minimal measurable expression level).

We adopted the approach of Turro et al. for combining lowly identifiable transcripts based on the posterior correlation of their expression-level estimates, tailored for a diploid transcriptome case [93]. In this approach, for any given RNA-seq sample, we compute the Pearson correlation coefficient of the posterior TPM samples of any pair of transcripts from the same locus and the same allele. Subsequently, if the mean Pearson correlation coefficient across all RNA-seq samples for a pair of transcripts in both alleles is lower than a defined cutoff (which we empirically set to − 0.25), each of these pairs is combined into a single transcript. This process continues iteratively until no pair of transcripts (or pairs of already combined transcripts) can be further combined. This consistency between the alleles in the combining process ensures that the resulting combined transcripts are identical for the two alleles and can therefore be tested for allelically biased expression.

### Amplification of full-length *Tug1*

We amplified the full-length *Tug1* isoform lacking the 5′ region (Ensembl ID: ENSMUST00000153313.2) from Riken cDNA clone E330021M17 (Source Bioscience) using specific primers containing MluI and EcoRV restriction sites (see the “Sequences and primers” section). After gel purification, the amplicon was sub-cloned, using the MluI and EcoRV restriction sites, into a modified Tet-On pTRE2pur vector (Clontech, 631013) in which the bGlobin-intron was removed. We verified the absence of mutations from the cloned *Tug1* cDNA by sequencing using primers listed below. The plasmid was used also for sub-cloning *Tug1* into pcDNA3.1(+) (see below).

### RT-PCR

The testes were collected from wild-type and *Tug1*^−/−^ mice (112 days old) and were homogenized in TRIzol (Invitrogen, 15596026) with a gentle MACS Dissociator (Miltenyi Biotec, 130-093-235). RNA was isolated by column purification (Qiagen, 74104) using a QiaCube (Qiagen). RNA was assessed and quantified on a Bioanalyzer (Agilent). cDNA was synthesized using 500 ng of total RNA as input with SuperScript IV VILO Master Mix with and without RT (Invitrogen, 11756050). PCR was performed on the synthesized cDNA (RT and no RT samples) using MyTaq Red Mix (Bioline, BIO-25043) with the cycling parameters: 95 °C for 1 min, followed by 35 cycles of 95 °C for 15 s, 60 °C for 15 s, and 72 °C for 10 s. PCR products were run a 1% agarose gel and verified by Sanger sequencing (GeneWiz). The following are the primers used for RT PCR: 7SK F1: GACATCTGTCACCCCATTGA and R1: TCCTCTATTCGGGGAAGGTC; Tug1 F1: CGGAGGAGCCATCTTGTCTTGTC and R1: GCTTCCAATTCCATACACACACTG; Tug1 F2: CTCTGGAGGTGGACGTTTTGT and R2: GTGAGTCGTGTCTCTCTTTTCTC.

### ORF search

We analyzed human and mouse *Tug1* cDNA sequences with CLC Genomics Workbench (Qiagen) for open reading frames (ORFs), allowing both canonical and non-canonical start codons (AUG, CUG, and UUG). After, sequences with annotated ORFs were aligned using MUSCLE alignment. All further sequence and amino acid alignments were performed with CLC Genomics Workbench.

### Generation of human and mouse TUG1-BOAT overexpression constructs

We generated a synthesized construct for human *Tug1* ORF1 that contained an in-frame 3xFLAG epitope tag prior to the stop codon, with and without the 5′ leader sequence (GeneWiz). We also synthesized a construct containing mouse ORF1 with an HA tag after the 3xFLAG before the stop codon, with and without the 5′ leader sequence (GeneWiz).

We amplified the *Tug1* cDNA sequence with primers (see the “Sequences and primers” section) having KpnI and NotI restriction enzyme overhangs from the pTRE2-*Tug1* vector plasmid using Q5 polymerase (Roche) and under the following conditions: 96 °C for 2 min, 35 cycles of (96 °C for 30 s, 65 °C for 30 s, 72 °C for 4 min), 72 °C for 4 min, and gel purified the amplicon. We digested the inserts and pcDNA3.1(+) plasmid with proper restriction enzymes according to the manufacturer’s instructions. After digestion, the plasmid was dephosphorylated using alkaline phosphatase. We then ligated the plasmid and inserts using T4 ligase (NEB) in a 1:3 ratio, respectively, followed by bacterial transformation, culture growth, and plasmid isolation (Qiagen Mini-Prep Kit).

### Transfection of TUG1-BOAT constructs

We seeded 3T3 and HeLa cells in 10-cm plates containing poly-l-lysine-coated 18-mm glass coverslips. Next, we transfected the cells with 14 μg of plasmid (pcDNA3.1(+) containing each of the inserts) using Lipofectamine 3000 Transfection Reagent (Thermo Fisher Scientific) per manufacturer’s recommendations. Forty-eight hours post-transfection, cell pellets were harvested for protein extraction (see below) and coverslips were processed for RNA FISH and/or immunofluorescence (see below).

### Protein extraction and western blot

We resuspended 3T3 and HeLa cell pellets in RIPA Lysis and Extraction Buffer 48 h post-transfection (Thermo Fisher Scientific). Total protein was quantified with Pierce™ BCA Protein Assay Kit (Thermo Fisher Scientific). We then separated a total of 20–25 μg of denatured protein on a 12.5% SDS polyacrylamide gel for 100 min at 120 V. We transferred proteins to an Immobilon-PSQ PVDF membrane (Sigma-Aldrich, ISEQ00010) at 400 mA for 75 min. After blocking in 5% dried milk in TBST, the membrane was incubated with properly diluted primary antibody (M2 Monoclonal ANTI-FLAG 1:1000, F1804, Sigma; Monoclonal GAPDH 1:5000, 2118S, CST) in 5% dried milk/TBST overnight at 4 °C. The next day, we washed the membrane three times for 5 min each in TBST (0.5% Tween-20). We then incubated the membrane with horse radish peroxidase-conjugated secondary antibody (anti-mouse 1:15,000, A9044, Sigma; anti-rabbit 1:10,000, 711035152, Jackson ImmunoResearch), diluted in 5% dried milk/TBST for 1 h at room temperature. Following three 5-min washes in TBST, SuperSignal™ West Pico PLUS chemiluminescent substrate (Thermo Scientific, 34580) was added and chemiluminescence was detected using ImageQuant™ LAS 4000 imager. Original, uncropped images of western blots are provided in Additional file 16: Fig. S11.

### TUG1-BOAT localization by immunofluorescence

We plated HeLa and 3T3 cells on poly-l-lysine-coated coverslips. Forty-eight hours post-transfection, we rinsed the coverslips twice with PBS and fixed the cells with 3.7% formaldehyde in PBS for 10 min at room temperature. After 2 washes with PBS, we permeabilized the cells with PBT (PBS, 0.1% Tween-20) for 15 min at room temperature. Next, we blocked the coverslips with 5% BSA in PBT for 1 h at room temperature and then incubated the coverslips with properly diluted primary antibody (mouse M2 monoclonal ANTI FLAG, 1:800, F1804, Sigma; rabbit polyclonal Tom20, 1:800, FL-145, Santa Cruz) in 5% BSA in PBT for 3 h at 37 °C in a humid chamber. Coverslips were washed three times for 5 min each with PBT and incubated with diluted secondary antibody (anti-mouse labeled with Alexa Fluor 488, 1:800, ab150113, Abcam; anti-rabbit labeled with Alexa Fluor 647, 1:800, 4414S, CST) in 5% BSA in PBT for 1 h at room temperature. Cells were then washed twice for 5 min with PBS, once for 20 min with PBS containing Hoechst DNA stain (1:1000, Thermo Fisher Scientific), rinsed in PBS, and then mounted on glass slides with ProLong Gold (Thermo Fisher Scientific).

### Mitochondrial staining with MitoTracker® red chloromethyl-X-rosamine

We plated cells on poly-l-lysisne-coated coverslips and transfected as described in the previous sections. Forty-eight hours post-transfection, cells were incubated with 200 nM MitoTracker red chloromethyl-X-rosamine (Thermo Fischer Scientific, M7512) in 1 mL FBS-free growth media for 40 min. We then washed the cells twice with PBS, fixed with 3.7% formaldehyde for 10 min at room temperature, and processed for immunofluorescence and/or RNA FISH (as described previously).

### Microscopy and image analysis

We acquired z-stacks (200 nm z-step) capturing the entire cell volume for single-molecule RNA FISH, single-molecule RNA FISH/CMXR staining, 3xFLAG tag immunofluorescence/CMXR staining, and/or Tom20 immunofluorescence with a GE wide-field DeltaVision Elite microscope with an Olympus UPlanSApo 100x/1.40-NA Oil Objective lens and a PCO Edge sCMOS camera using corresponding filters. 3D stacks were deconvolved using the built-in DeltaVision SoftWoRx Imaging software. Maximum intensity projections of each image were subjected for quantification using Fiji.

### Fluorescence-activated cell sorting

Age- and sex-matched adult mice were used in all flow cytometry experiments. We obtained peripheral blood by cardiac puncture and collected blood into a 1.5-mL Eppendorf tube containing 4% citrate solution. Next, we added the blood-citrate mixture to 3 mL of 2% dextran/1× PBS solution and incubated for 30 min at 37 °C. The upper layer was transferred to a new 5-mL polystyrene FACS tube (Falcon, #352058) and centrifuged at 1200 rpm for 5 min at 4 °C. We then lysed red blood cells for 15 min at room temperature using BD Pharm Lyse (BD, 555899). Cells were washed twice with staining media (Hank’s Balanced Salt Solution (HBSS) containing 2% FBS and 2 mM EDTA). The following antibodies were added (1:100) to each sample and incubated for 30 min at room temperature: Alexa Fluor 700 anti-mouse CD8a (Biolegend, 100730), PE/Dazzle-594 anti-mouse CD4 (Biolegend, 100456), APC anti-mouse CD19 (Biolegend, 115512), Alexa Fluor 488 anti-mouse NK-1.1 (Biolegend, 108718), and PE anti-mouse CD3 (Biolegend, 100205), and Zombie Aqua Fixable Viability Kit (Biolegend, 423101) was used as a live-dead stain. We washed samples twice with staining media and sorted directly into TRIzol LS using a BD Aria FACS.

### qRT-PCR

We isolated and quantified RNA from sorted blood populations as described in the RNA Isolation and RNA-Seq Library Preparation. One hundred nanograms of total RNA was used as input to generate cDNA using SuperScript IV VILO Master Mix (Invitrogen, 11756050), according to the manufacturer’s protocol. cDNA was diluted 1:3 with DNase- and RNase-free water, and 1 μL was used per each reaction. We performed qRT-PCR using FastStart Universal SYBR Green Master Mix with ROX (Sigma, 4913914001) on a ViiA 7 Real-Time PCR System (Thermo Fisher). Analysis was performed using the ∆∆Ct method [94]. Primers used in qRT-PCR experiments are listed in the “Sequences and primers” section.

### GEO accession numbers

All primary RNA-seq data are available at the Gene Expression Omnibus (GSE124745 and GSE88819).

## Sequences and primers

### In situ hybridization riboprobe—mouse *Tug1* (492 bp)

GAGACACGACUCACCAAGCACUGCCACCAGCACUGUCACUGGGAACUUGAAGAUCCAAGUUUCUGUCCAGAACCUCAGUGCAAACUGACAACACUCCAUCCAAAGUGAACUACGUCCCGUGCCUCCUGAUUGCUGAAUGUUCACCUGGACCUGCCAAUGACCUUCCUUCUGCUACUCCAUCAGCCUACAGACCUGGUACUUGGAUUUUUGUCCAUGGUGAUUCCUUCCACCUUACUACUGAAGAAGACACCAUUCCAGUGGACCACUGUGACCCAAGAAGCAUUCAGCCAUCAUGAUGUGGCCUUUACCUCCACUCCUGUCCUACUCUGCCCAGAUUCAGCACAGCCCUUUAUAGUGCAGUCAAGAGUCUUCAAGCCAAAUAACUGAAGCUAUUUUAUCACAACAAAGGCCAGGUUUAUUCCAUAAAUGUACAGUUCAUUUCUGCAGUUUAUUCUUCAGAGACACAUAGUAAAUUUGGACCAGGGGAUUUUG

### Genotyping primers

#### *Tug1*^*tm1.1Vlcg*^-knockout mice and MEFs

Tug1_5190-5166TD_Forward:TGACTGGCCCAGAAGTTGTAAG

Tug1_5190-5166TD_Reverse:GCAAGCAGGTCTGTGAGACTATTC

lacZ_5_Forward:TTGAAAATGGTCTGCTGCTG

lacZ_5_Reverse:TATTGGCTTCATCCACCACA

Ychr_Forward:TCTTAAACTCTGAAGAAGAGAC

Ychr_Reverse:GTCTTGCCTGTATGTGATGG

### Mouse *Tug1* (ENSMUST00000153313.2) cDNA clone

TTTTTTTTTTTTTTTTTTTTTTTTTTTTTGGGGGGGTTTTTTGTTTTGTTTTTTAAATTGAAGGCTAAAGTTTTTGAAAAAACTTTGTTGGACTCTGGCTGGGACACAAAATCAGATATTTGGAATCATTTTGAAGCTTAACTTTTTCCTAACCAGCCTTGTATTCTAATTGCTTGCAAATGTGAGACTGAATGGCCAAAATGCCGTTTGTTTGTTTGTTTATTGTCAGCTGCTTTTATCAAATTCCAGGCCATTATCCAGCAAACACTATTAAAATGTTTGAACAGTTGGGTTTCAAACATTTTTGTTTTGTGGAGTGGTGCTTATTAAGTGGTACAGCTCTCTAAGCAAGTGAACACAAACATATTTAAGTGTATTTTGTATGATTAGATGTTACCAATTCTGATATTTTATTCAAATGTCTAAAAAAATAAGTTGACTTATTCCCTTTACCAAAGGGCCAGAGACAAATGGTTTCCTTTCAAGAGAAATGACTGTTTTGAAGAAAAACTCTGTTGGTCTTAGCTCTTTTGTAATTAAATCTGGATGTACCTCAAAAGACTCTTTAAGACTGTGGTGTTAAAAGGCTTTCCTCTGGAGAAGGAGAAAAAATAAAATCAACTGGAACTTAAAAGCTTGAAATTCCATGACAAAACACAGATGTCCAGGATTGGAGGTTCATAAAGTACATGCAGTAGTTGGAGTGGATTCCATTTTCAGTGTAGCTGCCACCATGGACTCCAGGCTCCCAGATTTTCAAGAACTGGACCTGTGACCCAGAAGAGCTTGTCAAGATATGACAGGAACTCTGGAGGTGGACGTTTTGTATTCAATTTTGGAACTGTTGATCTTGCCGTGAGAAAAGAGAGACACGACTCACCAAGCACTGCCACCAGCACTGTCACTGGGAACTTGAAGATCCAAGTTTCTGTCCAGAACCTCAGTGCAAACTGACAACACTCCATCCAAAGTGAACTACGTCCCGTGCCTCCTGATTGCTGAATGTTCACCTGGACCTGCCAATGACCTTCCTTCTGCTACTCCATCAGCCTACAGACCTGGTACTTGGATTTTTGTCCATGGTGATTCCTTCCACCTTACTACTGAAGAAGACACCATTCCAGTGGACCACTGTGACCCAAGAAGCATTCAGCCATCATGATGTGGCCTTTACCTCCACTCCTGTCCTACTCTGCCCAGATTCAGCACAGCCCTTTATAGTGCAGTCAAGAGTCTTCAAGCCAAATAACTGAAGCTATTTTATCACAACAAAGGCCAGGTTTATTCCATAAATGTACAGTTCATTTCTGCAGTTTATTCTTCAGAGACACATAGTAAATTTGGACCAGGGGATTTTGTTTTGTTTATATTGTCAACACTGTCTGAAGAAAGGCATCTCTGAGAACAGCATTGGACCCTACTCCACAATCTCAAATGATTGAAGTTTCATAAACTGCCTAGGATCCTGTCAAGGCCACTGGACTCTTGTTCTTTTCCTACTTCAAAATCTGTAGCTGTCTACTAAATGACAAAGCAGATATTCTGACCCATTGGGATCAAAACCAAGGCATTTTGAATTCCTCATAGTATCATCTTCGGGTTACTCAGGAACCAAAACTTTTCACACCAATTTAAGAAATTCTACTGAGGAATCCCTTTACCTAACCATCTCACAAGGCTTCAACCAGATTCCTGAAAAGGCCTCTTGATATATCAAGATAGAACCTACATGCATTTTGTGAACAACTTATCACTGATTTTCCAAAGGCTTTGTGCTCTTGAAGTTCTTTGAAGGAAAGCTGTGTGGAAGTCCAGAGTAAAGTGAAGCTGCTCTGGATGAAGTAGTGAAGTGGGAGTTGAGGTCTACAACCTGCCACAACCATCTTCCTTTACCACCATGGTGATGCCAAAAGGGACTTCCTTAAAGCTCTTCAGAAAATCCTGCTTGAAACCACTACCCTAGACAACATGTTTGACCTGGATGGCATTCTCTTCAAAACAATTCATATTCAGTTGATGCTCAACATGTTTGGAGATGCTTTATTCAGAGAATGATGATAATTACAGCATTGTCTAATGAAGTTTTATTAATAGCATTCCATCCAAGGTGGACTTCCTGGAGTTGGATATAACCAGAGAGCAATTCATATGTATCCTACACTGAAGAACACCATTAACTTTCAGCAACCTATAGCTAGTGGTACTAGAAGTACGTGTCTTGGAAGTCTATGAGAGCTGGTATTGAAGCTGATGCCTCCTTAAGGCCATCTTAGACCAAGTTGTTTGTTTGACCTCTCCTCATTAACTATGGAGCAGAATTGAAATACAAATTTTTCCTAAAGGGACTTGCAACCTGGTTATCATTCATTATCTCAAGTTTCAAGTCATGTTGATGCAACCAGTAGTTATTAAACTGCTCCATGGTTTTTTGTTATTTAATACTTTTTCCAGGGCTTAAAAAAACAAAATTAAATTTCTCCAACACGTCTATACTTGTCTGTTCAAAAGTAACTACTCACCACTATATGGAACAGATGATTCTGAAGACACTCTGAGCATCCTTTATGATATTTGTGACTTAAAATGTGGCTGGAAATTTTCCTTCTACCCAGTGAAATATTTAATGATTAGTCTTCATGCCTGATACCATCAACTGTATATGCGTGATAGGCAAAGTTTGACATAGGCATTTGACTCTAGGCTATGATAGCTTGCTAGTAACTTCAAGTAGCATATTGTCAACCTGTTTGCTGGAAAAGTAGAGTAACTTGGAAAAAAAACTAAATGGCAGCTAAGGATTTTTTTCAGTATTCCTGAGTTTCTGTCCTTGGGATATTTCAATGAAATTTTCACCTGTCTCTTCACTTAACAGAGTGACTGACTCCTTACTATGAAGTATTCTTAAGACATTAAGATTACTTTTGTAGAAAGGATAAAATTCCTGACCATCCAAATCATCATAGTGAACAAGACTTCAATTTGTGACCTGAGAAAATCTCATTTCTCTACTTCGTAGTCAATGTAAGGGCCAATGCTATCAGCTACTCTGAGTGCACTGGGTAAACGTTGGAACTGCCTTCTTTATATCATTACTTTTTATCCTCTAAATTAATCATGGTTATGTAATTCTCGCCACAAATCAGCAAATCAGACTCAGATCTGGTTATTCTAGACTGCTCACAGTTAACAAATCAAACTCTGGATGACTTCTGCTTGTATATGCAACTACTATTTGTAAAGAAATTGCAAATTCACTTTTCTATTACCTCTACATTGCTAGCTCTTTCTTTTGTGTTTGTATTAAAAACAAAAATAAGCTACACTGCCAGCTATTCCCTCCTGCCATACTCAGTTAAAATGAAGAATCGGGAATCTAACCAGTGAATGGATAAGTAGAAAAAACTAAAACTTAAGGCAAAAGCCTTAATCTAGGGCCTTTTCTACTATCTTCATGTCTTGGATTTCATCTAAAATCAACAGTGCCACCCAACCAGTCTGAGGTCTTGACTTGCTTTTAAGATGATTCTTAGAGATGGGCTGTATTACAGAAGGTGAAGACTTGATTACCAAAGAAAGTAGAGCCAACTTTGACAAACCTGGCTCTACAATCCTATTGCTTCCAGATGTAGCATAGACTCATAACTAGAACCTCAAGTCTGCATTGAGGATATAGCCTTCTAAGCTGACAGTTCTTGCAACAGGTGAGCAAGAAAATGAAAGCTGTTATACCCAACTGGCCCTTTAAGATCCAAAAATAATGTCTGGACTAAACCCTATGGAGTACCCAGGACAAAAACTAATTTACAGAGCTTCATTATTAATCTGCCTGTTCTTCTAGCTTAATTATTGGTATGGCTGGCCCTACTGAAGTAGTTTGTCTGTTTACCTGTCTTCAGCTCTTAACCTGGCTATTTTGACATGCTACTGCAATTAGACTAACTGGCTTTGAGAAGACTACAATCAGTTTCAGCCTCTCCTTTGCCCAATTTCACCAAGGAATTTTGATAAGAGGAACCCATACCTCACCCCACCAGAACAGAAAGGACCATGCTGCATATTCCTTGACCAGCAACTTTAAGTAGAGAACAACCCTGCTTGTTTTCAACATCTGAAACACCATTTGATCTAATAGGAGTATAGAAGGTTGACAGCAGAGTACACTACTTACTTCTTTCATAACTCAGAAATGAATATGACTGGCCCAGAAGTTGTAAGTTCACCTTGACAAGAAACAGCAACACCAGAAGTTTACTGCTGAACTTAACTTGCCACTTACTCGAATAGTCTCACAGACCTGCTTGCCAAGTAGGAGGCTAGTTTTCCTGCTTCATATCACCATTGGAGTGGGGCTCAATGGGGTCAATGTTAATACTGACTTGAATGGGGACCTTATGGTGAATCCTAGACTATGAGGCTAATGGAAATTATTGTCTATTCAAGTGGATTATAGATTTCCTGAGGACAGAACAGACATCACTCCTGGTGATTTTTAGAACTTGATTACCAAGGAAGAAATACCAGCTGCTAACAGTCAACTTCATGGGCAAAGATTAAGCTCTCTATATCTGGTCGTATCCTGGATGCTAGTTTTTTATTGCCCAGTGACCATTTCCATCTCACGCTTAACTTCCTGATGTTTTTTGGAACCATCTCTTCCAATTTTCAGTCCTGGTGATTTAGACAGTCTTTTCATGCTGGACATTTTGTTGCAACCTCATCAATCACAGCAAAGTCCATCTTGACTTTAGTGATTAGTTCAGGAATGGATGCATGATTCAAGTTTGTCCAATGATAATCAACCCTAGGTGTTTTCTCAGTTGTGGAGAAGTTCTCTTAGATGCTTTAGCTTTGTAGGAGAAAACTCAAACCAACAGGGCCTACCTACTATGTTGAATGATTGTAGGAGAAAACTCAAACCAACCAGGCCTACCTACTATGTTGAATGAGCCAGGCAGAAAATGAAGCCAGTACAGAGGGAAATGGAGCCAAAAGAGGAAGAGACTTGAGTTCTGATGATCACATTTATGCCCCTGTATCCAACTGTGCCTGAAGCTAATAGTACATCACCTGGACTTTTCAGTTATGTGAACCAATAAATTCCCCTTTTTGTTTAAGTTACTTTGAGTT

### Full-length *Tug1* primers for cloning in pTRE2pur

Tug1_MluI_cDNA_Fwd gagaacgcgtTTTTTTTTTTTTTTTTTTTTTTTTTTTTGGGGGGGTTTTTTGTTTTGTTTTTTAAATTGAAGGCTAAAGTTTTTGAAAAAACTTTGTTGGACTCTGGCTGG

Tug1_EcoRV_cDNA_RevgagagatatcAACTCAAAGTAACTTAAACAAAAAGGG

### Primers for full-length sequencing of pTRE2-*Tug1* expression vector

LNCXAGCTCGTTTAGTGAACCGTCAGATC

TUG1_76TTTAAATTGAAGGCTAAAGTTTTTGAA

TUG1_266GGCCATTATCCAGCAAACAC

TUG1_755ACTCCAGGCTCCCAGATTTT

TUG1_1254TCTTCAAGCCAAATAACTGAAGC

TUG1_1741AGAACCTACATGCATTTTGTGAA

TUG1_2268ATGCCTCCTTAAGGCCATCT

TUG1_2754TGTCAACCTGTTTGCTGGAA

TUG1_3267TTGCAAATTCACTTTTCTATTACCTC

TUG1_3746CCCAACTGGCCCTTTAAGAT

TUG1_4241TGACAAGAAACAGCAACACCA

TUG1_4740TCACAGCAAAGTCCATCTTGA

### Primers for sub-cloning full-length *Tug1* from pTRE2-*Tug1* into pcDNA3.1(+) expression vector

Tug1_Tg F/KpnIataggtaccGCCCCGAATTCACGCGTT

Tug1_Tg R/NotIatagcggccgcACCTGAGGAGTGAAGA

### Primers for qRT-PCR

Tug1_FwdCTCTGGAGGTGGACGTTTTGT

Tug1_RevGTGAGTCGTGTCTCTCTTTTCTC

Gapdh_Fwd GGTGAAGGTCGGTGTGAACG

Gapdh_Rev CTCGCTCCTGGAAGATGGTG

### Human TUG1-BOAT (ORF1) sequences with 3xFLAG (blue) for expression construct design

Human ORF1:

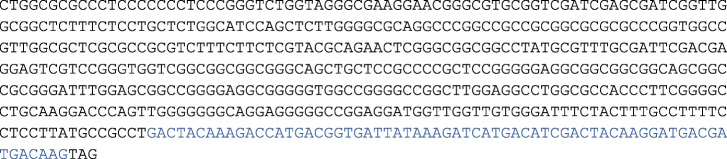



Human ORF1+UTR:

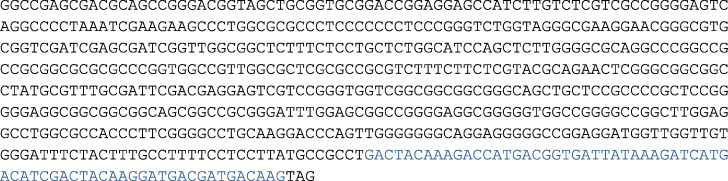



### Mouse TUG1-BOAT (ORF1) sequences with 3xFLAG (blue) and HA (red) tags for expression construct design

Mouse ORF1:

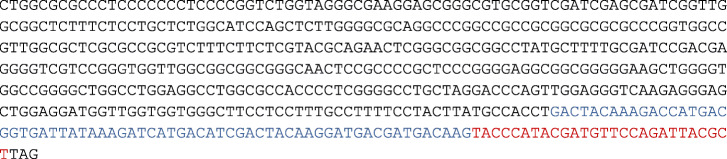



Mouse ORF1+UTR:

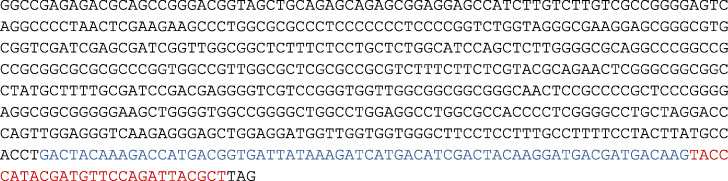



## Supplementary information

**Additional file 1:** **Fig. S1**. Mouse and human *Tug1* locus and chromatin context in different cell types. **(A)** *Tug1* mouse and **(B)** human genomic loci. Evolutionary nucleotide conservation (PhyloP) of the locus are presented along with the chromatin context (DNase I hypersensitive regions, histone modifications) and protein binding ChIP-seq peaks (Pol2, CTCF, SIN3A, COREST, SETDB1, HDAC2) from ENCODE (UCSC Genome Browser, mm9) datasets in the indicated cell types.

**Additional file 2:** **Fig. S2.** In vivo expression pattern of *Tug1* during murine embryogenesis. RNA in situ hybridization of *Tug1* RNA using a digoxigenin-labeled antisense RNA probe in mouse embryos at different developmental stages. Embryonic day (E)8.5, E9.5, E10.5, E11.5, and E12.5 are shown.

**Additional file 3:** **Fig. S3.** Overview of the *Tug1* locus in mouse. UCSC genome browser showing the murine*Tug1* locus. The three predominate *Tug1* isoforms are depicted (black) and the *Tug1* transgene (tg(*Tug1*)) is shown (blue). For *Tug1* knockout, the longest annotated *Tug1* isoform was replaced by a *lacZ* reporter cassette, leaving the promotor and first exon intact. The deleted region is indicated by red dashed lines. The open reading frame (ORF) encoding the TUG1-BOAT protein and PhyloCSF scores for the (-2) frame across the locus are depicted (grey). Chromosomal coordinates (mm10) are shown.

**Additional file 4:** **Fig. S4.** Morphology analysis of *Tug1*^-/-^ mice and sperm **(A)** Body mass (g) measurements over 11 weeks of male and female *Tug1*^-/-^ mice compared to wild type littermates. Males: *Tug1*^-/^- (*n* = 7); WT (*n* = 8). Females: *Tug1*^-/-^ (*n* = 3), WT (n = 7)**.** Significant *p* values at specific time points are indicated (*). **(B)** Representative images from adult male mice (12 weeks old) show normal physiological appearance of external genitalia and reproductive tracks in *Tug1*^-/^- compared to WT. Seminal vesicles (SV), vas deferens (VD), bladder (B), testicle (T), epididymis (E), anterior prostate (AP). **(C)** Box plots of body mass (g) (left panel), relative testis mass (testis mass / body mass; middle panel) and total sperm count for wild type (*n* = 9) and *Tug1*^*-/*^*-* males. **(D)** Box plots of the percentage of different sperm morphological abnormalities for wild type (n = 9) and *Tug1*^-/-^ (n = 8) males. Significant (*) p value (Wilcoxon rank sum test) is indicated**.**

**Additional file 5:** **Table S1.** *Tug1*^-/^- and wild type sperm morphological defects.

**Additional file 6:** **Table S2.** Testes RNA-seq and *Tug1*^rescue^ RNA-seq in testes.

**Additional file 7:** **Table S3.** Prostate, spleen, eyes, brain, heart, liver, and MEF RNA-seq.

**Additional file 8:** **Table S4.** Allele-specific RNA-seq in testes, expression data.

**Additional file 9:** **Table S5**. Allele-specific RNA-seq in testes, differentially expressed genes.

**Additional file 10:** **Fig. S5.** Gene expression of multiple tissues in *Tug1* WT and KO mice. Gene expression of multiple samples (columns) were described with log scale of TPM: Log_2_(TPM+1). Genes (rows) are clustered by hierarchical clustering with Ward’s method based on Euclidean distance. Annotation of samples are provided in the top panel in terms of genotypes and tissues.

**Additional file 11:** **Fig. S6.** *Tug1* transgene expression and fertility assessment. **(A)** qRT-PCR for *Tug1* RNA expression in testes and sorted peripheral blood populations: WT (*n* = 1), *Tug1*^+/-^ (*n* = 1), *Tug1*^-/-^ (*n* = 1), and *Tug1*^rescue^ (*n* = 1) and sorted peripheral blood populations. Error bars indicate the relative quantification minimum and maximum confidence interval at 98%. Not detected (n.d.). **(B)** Representative flow cytometry gating strategy for NK, CD4, and CD8 cells in peripheral blood from WT, *Tug1*^+/-^, *Tug1*^-/-^, and *Tug1*^rescue^ mice (gating from WT peripheral blood shown). **(C)** Scatter dot plot (mean with standard error of the mean shown) of the number of pups at birth per copulatory plug for matings using male wild type, *Tug1*^*+/-*^; tg(*Tug1*); rtTA, *Tug1*^*-/-*^, or *Tug1*^rescue^ (on dox diet) with wild type C57BL/6J females. Each dot represents a litter from a different mouse. **(D)** Sperm count from control (WT and *Tug1*^+/-^, *n* = 2), *Tug1*^-/-^ (n = 2), and *Tug1*^resuce^ (n = 3) mice. Each dot represents a different mouse and the error bars indicate the standard error of the mean. **(E)** Hematoxylin and eosin staining in *Tug1*^+/-^, *Tug1*^-/-^, and *Tug1*^rescue^ testes and epididymis. **(F)** Morphological analysis of sperm from *Tug1*^-/-^ (n = 2), and *Tug1*^rescue^ (n = 3) mice.

**Additional file 12:** **Fig. S7.** Changes of gene expression in two comparisons of *Tug1*^-/-^ (KO) vs. WT and *Tug1*^rescue^ (Rescue) vs. *Tug1*^-/-^ (KO). Each dot represents a gene whose x-axis value is the fold change of gene expression between KO and WT and y-axis value shows the fold change in Rescue vs. KO. Color describes statistical significance of fold change (adjusted *p*-value < 0.05): no statistical significance in either comparison (gray, *N*=33688); significance in KO vs. WT (blue, *N*=998); significance in Rescue vs. KO (orange, *N*=126); significance in both comparisons, KO vs. WT and Rescue vs. KO (red, *N*= 52).

**Additional file 13:** **Fig. S8.** The 5’ region of human *TUG1* contains a conserved ORF. **(A)** GWIPS-viz tracks for human *TUG1* genomic locus (hg38) is shown. Global aggregate of ribosome occupancy (ribosome profile), RNA-seq (mRNA coverage), and evolutionary protein-coding potential (PhyloCSF) across the *TUG1* locus is shown. ORF1 and ORF2 are outlined with red and gray boxes, respectively. Tracks surrounding both ORFs are zoomed in for clarity (bottom). **(B)** Scheme showing human ORF1 construct design. hORF1 (labeled with a 3xFLAG epitope tag prior the stop codon) with the 5’UTR was inserted into pcDNA3.1(+) and transfected into HeLa cells. 48 hours post-transfection, TUG1-BOAT-3xFLAG localization was analyzed by immunofluorescence (IF) (shown in C). **(C)** Maximum intensity projection of HeLa cells expressing human 5’UTR-hORF1-3xFLAG. Localization of 3xFLAG tagged TUG1-BOAT was assessed by immunostaining against the 3xFLAG (green). Nucleus was monitored by DAPI (blue) and mitochondria was monitored by immunostaining against mitochondrial membrane translocase TOM20 (gray). Bar plot shows localization analysis of TUG1-BOAT. Scale bar is 5 μm.

**Additional file 14:** **Fig. S9.** GFP over-expression does not compromise mitochondrial membrane potential. Maximum intensity projections of z-stacks acquired 48 h post-transfection of 3T3 cells with GFP cloned into pcDNA3.1(+) under CMV promoter and staining with Chloromethyl-X-rosamine (CMXR). GFP (green) was used as control. CMXR is shown in red, DAPI in blue. On the right, quantification of cells expressing GFP and mitochondria membrane potential by CMXR (*n* = 50). Scale bar is 5 μm.

**Additional file 15:** **Fig. S10.** Loss of TUG1 expression in infertile human males. Heatmap of microarray data from three different probe sets showing decreased expression of TUG1 in sperm from infertile teratozoospermic men (T) compared to fertile (normospermic) individuals (ND). In all cases, *p* < 4.39 x10^-4^.

**Additional file 16:** **Fig. S11.** Uncropped western blot images from Figure 5e. Original, uncropped images of western blots shown in Figure 5e. **(A)** Western blot of constructs overexpressed in 3T3 cells targeting the 3xFLAG tag (top). GAPDH is used as a loading control (bottom). **(B)** Western blot of constructs overexpressed in HeLa cells targeting the 3xFLAG tag (top). GAPDH is used as a loading control (bottom).

**Additional file 17.** Review history.

## Data Availability

The datasets generated and/or analyzed during the current study are available in the Gene Expression Omnibus repository, GSE124745 and GSE88819.
