# Supplementary figures and images for: The Tug1 lncRNA locus is essential for male fertility
Source: Genome Biol. 2020 Sep 7;21:237. doi: 10.1186/s13059-020-02081-5 (PMC7487648; doi:10.1186/s13059-020-02081-5)

**A**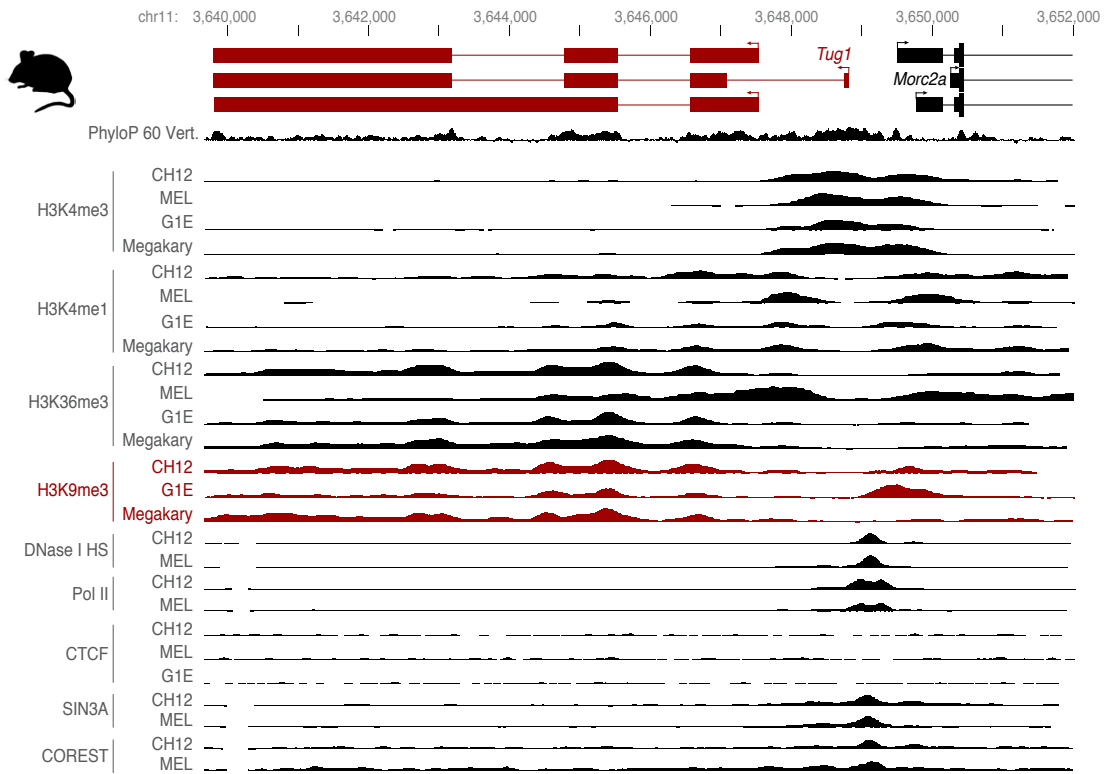**B**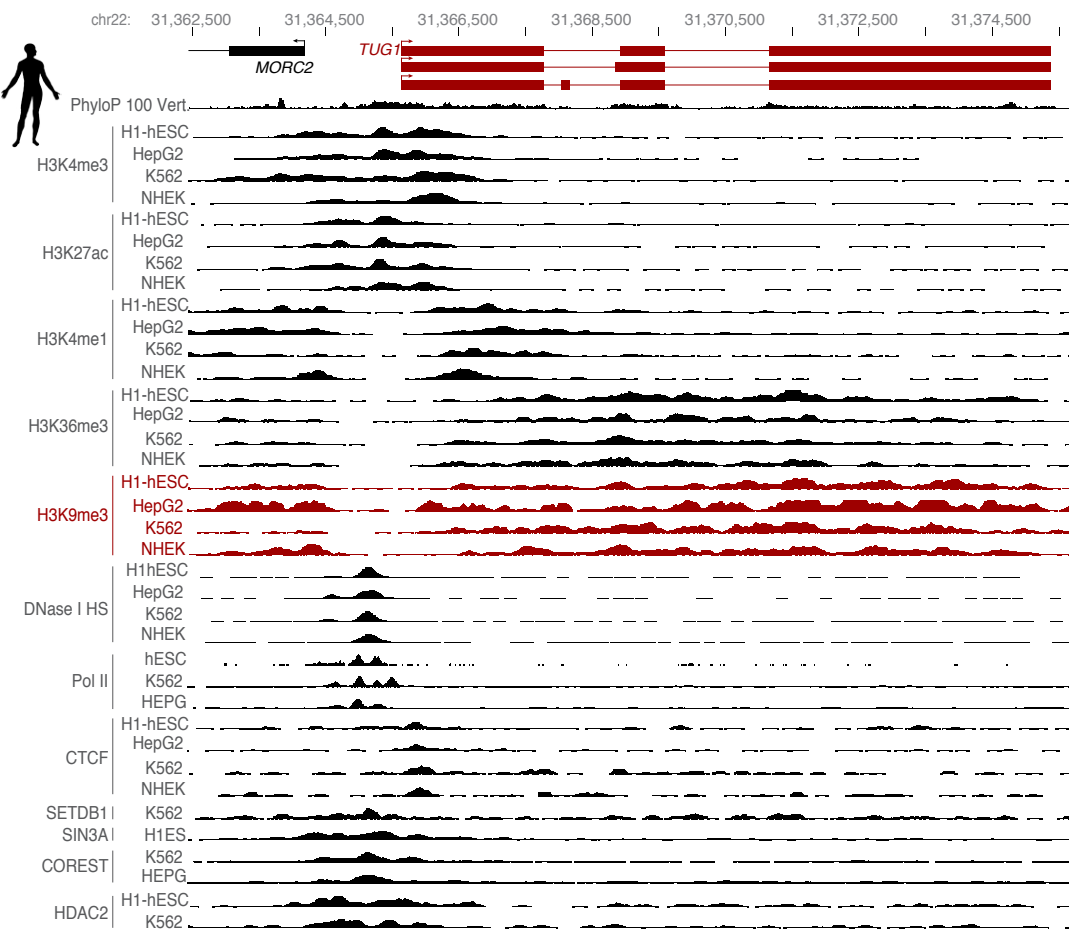

Supplement: Supplementary file 1 — Additional file 1: Fig. S1. Mouse and human Tug1 locus and chromatin context in different cell types. (A) Tug1 mouse and (B) human genomic loci. Evolutionary nucleotide conservation (PhyloP) of the locus are presented along with the chromatin context (DNase I hypersensitive regions, histone modifications) and protein binding ChIP-seq peaks (Pol2, CTCF, SIN3A, COREST, SETDB1, HDAC2) from ENCODE (UCSC Genome Browser, mm9) datasets in the indicated cell types. [file 13059_2020_2081_MOESM1_ESM.pdf]

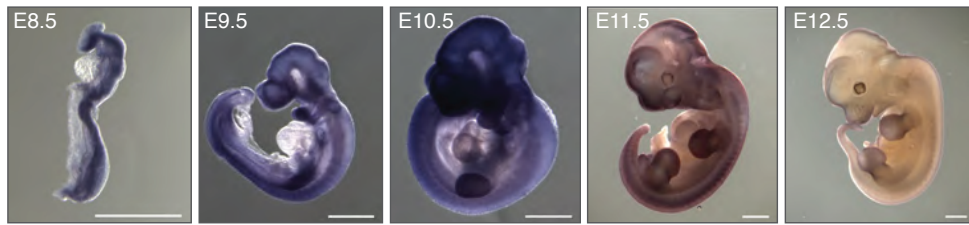

Supplement: Supplementary file 2 — Additional file 2: Fig. S2. In vivo expression pattern of Tug1 during murine embryogenesis. RNA in situ hybridization of Tug1 RNA using a digoxigenin-labeled antisense RNA probe in mouse embryos at different developmental stages. Embryonic day (E)8.5, E9.5, E10.5, E11.5, and E12.5 are shown. [file 13059_2020_2081_MOESM2_ESM.pdf]

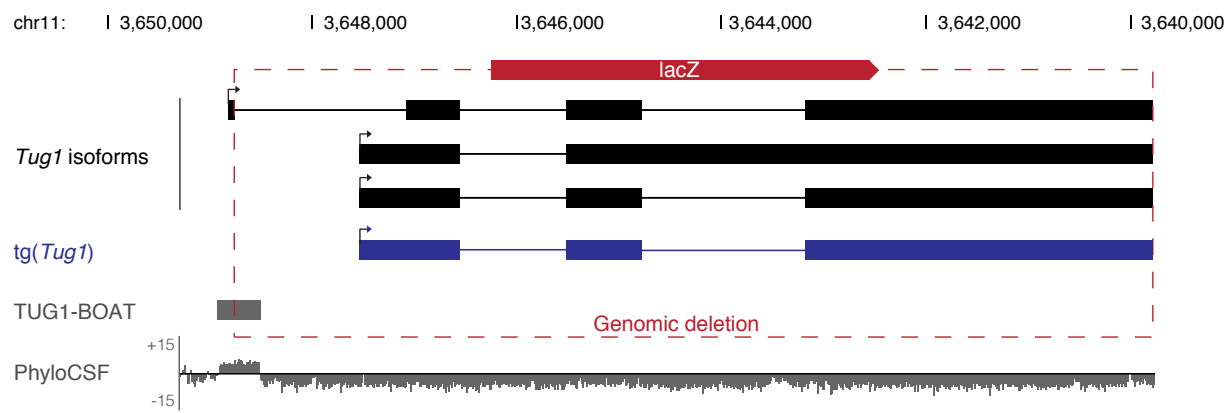

Supplement: Supplementary file 3 — Additional file 3: Fig. S3. Overview of the Tug1 locus in mouse. UCSC genome browser showing the murineTug1 locus. The three predominate Tug1 isoforms are depicted (black) and the Tug1 transgene (tg(Tug1)) is shown (blue). For Tug1 knockout, the longest annotated Tug1 isoform was replaced by a lacZ reporter cassette, leaving the promotor and first exon intact. The deleted region is indicated by red dashed lines. The open reading frame (ORF) encoding the TUG1-BOAT protein and PhyloCSF scores for the (-2) frame across the locus are depicted (grey). Chromosomal coordinates (mm10) are shown. [file 13059_2020_2081_MOESM3_ESM.pdf]

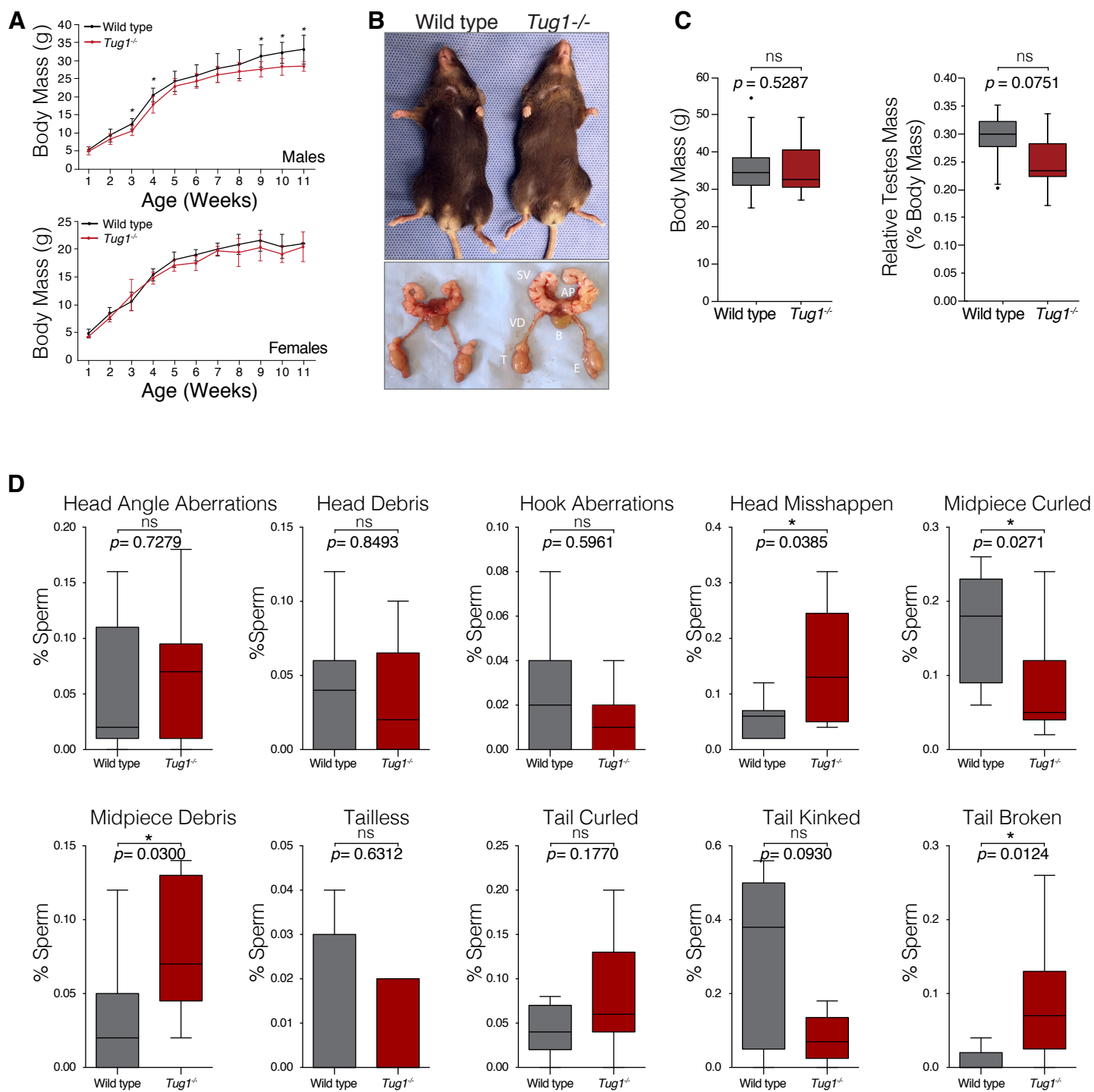

Supplement: Supplementary file 4 — Additional file 4: Fig. S4. Morphology analysis of Tug1-/- mice and sperm (A) Body mass (g) measurements over 11 weeks of male and female Tug1-/- mice compared to wild type littermates. Males: Tug1-/- (n = 7); WT (n = 8). Females: Tug1-/- (n = 3), WT (n = 7). Significant p values at specific time points are indicated (*). (B) Representative images from adult male mice (12 weeks old) show normal physiological appearance of external genitalia and reproductive tracks in Tug1-/- compared to WT. Seminal vesicles (SV), vas deferens (VD), bladder (B), testicle (T), epididymis (E), anterior prostate (AP). (C) Box plots of body mass (g) (left panel), relative testis mass (testis mass / body mass; middle panel) and total sperm count for wild type (n = 9) and Tug1-/- males. (D) Box plots of the percentage of different sperm morphological abnormalities for wild type (n = 9) and Tug1-/- (n = 8) males. Significant (*) p value (Wilcoxon rank sum test) is indicated. [file 13059_2020_2081_MOESM4_ESM.pdf]

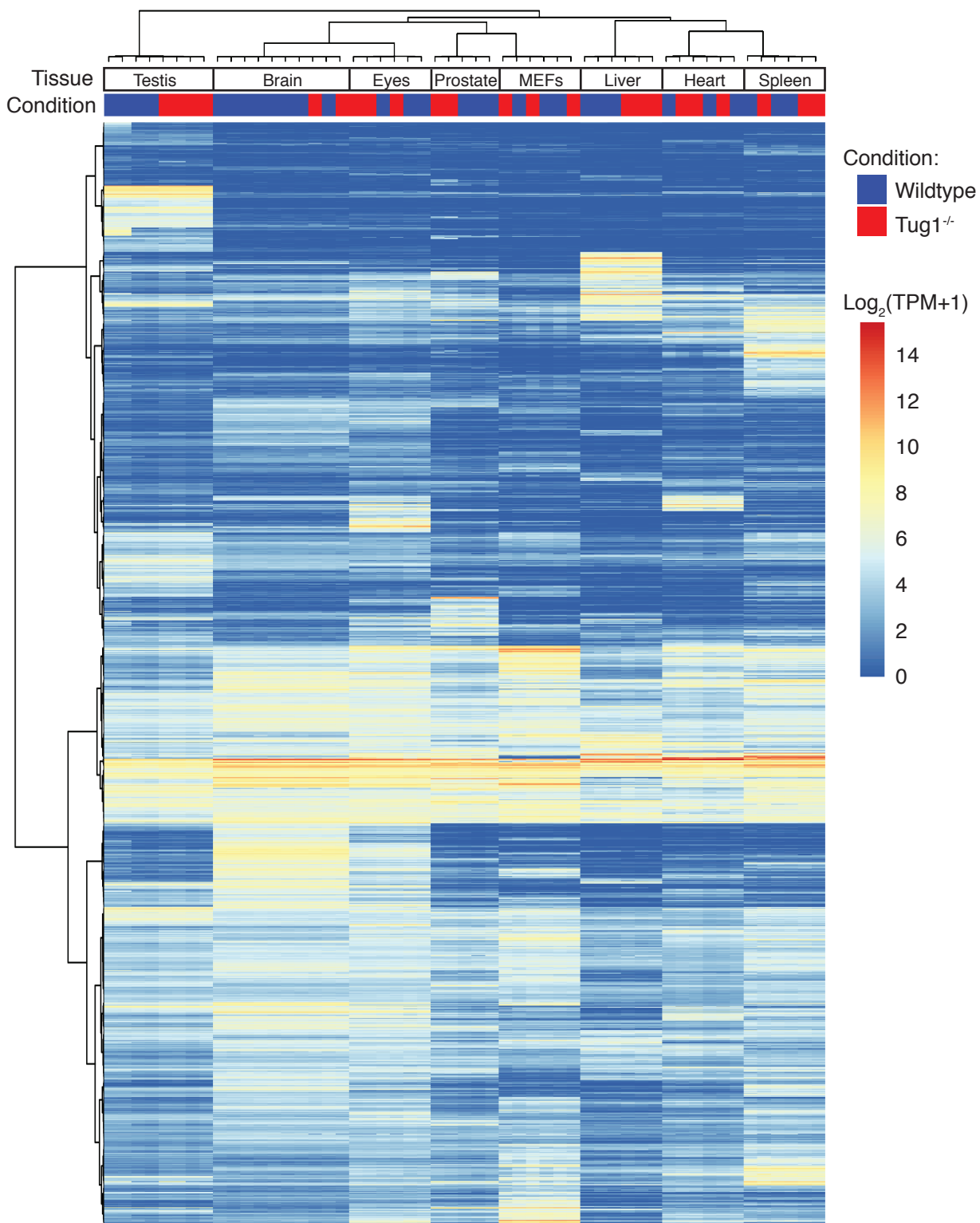

Supplement: Supplementary file 10 — Additional file 10: Fig. S5. Gene expression of multiple tissues in Tug1 WT and KO mice. Gene expression of multiple samples (columns) were described with log scale of TPM: Log2(TPM+1). Genes (rows) are clustered by hierarchical clustering with Ward’s method based on Euclidean distance. Annotation of samples are provided in the top panel in terms of genotypes and tissues. [file 13059_2020_2081_MOESM10_ESM.pdf]

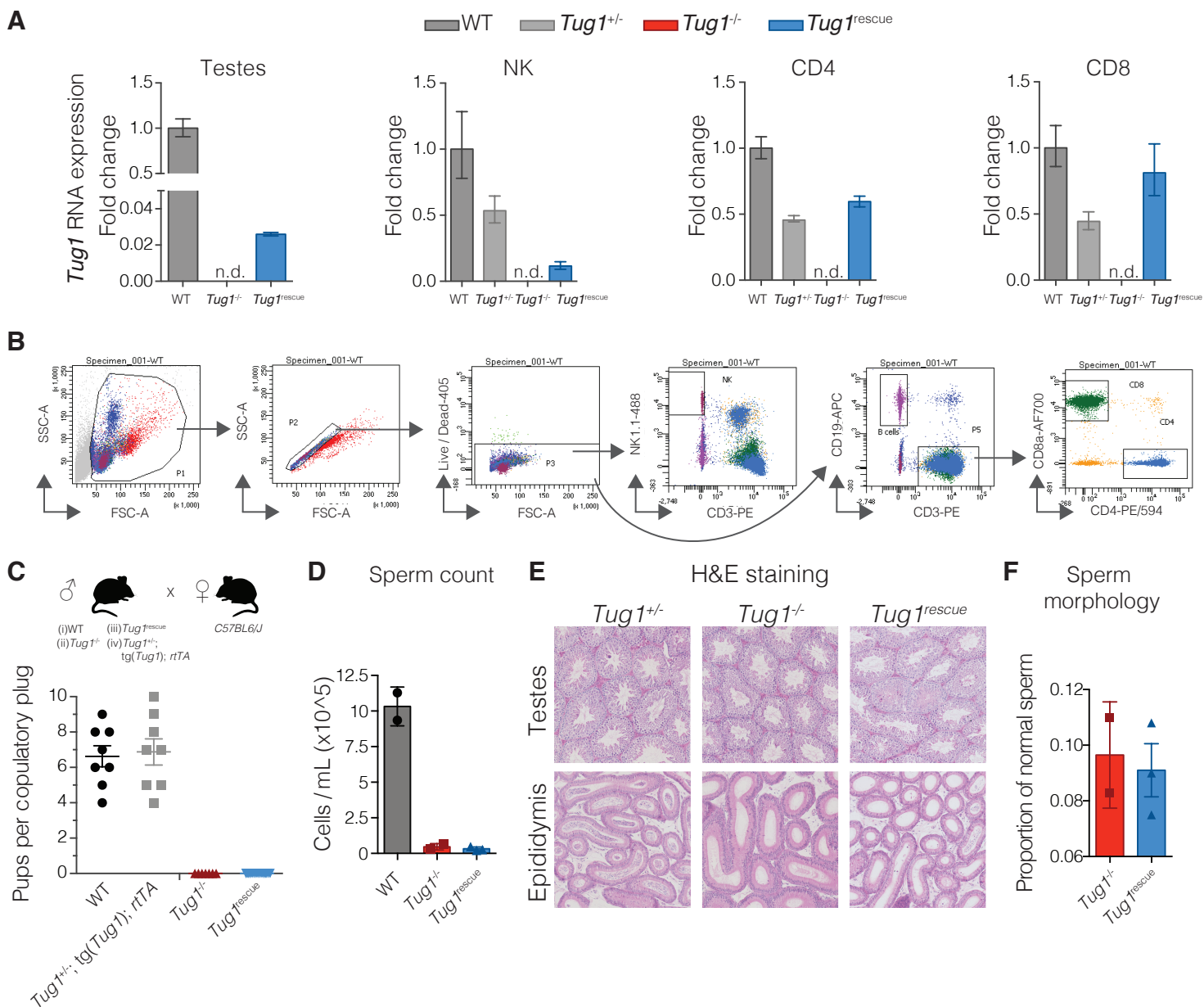

Supplement: Supplementary file 11 — Additional file 11: Fig. S6. Tug1 transgene expression and fertility assessment. (A) qRT-PCR for Tug1 RNA expression in testes and sorted peripheral blood populations: WT (n = 1), Tug1+/- (n = 1), Tug1-/- (n = 1), and Tug1rescue (n = 1) and sorted peripheral blood populations. Error bars indicate the relative quantification minimum and maximum confidence interval at 98%. Not detected (n.d.). (B) Representative flow cytometry gating strategy for NK, CD4, and CD8 cells in peripheral blood from WT, Tug1+/-, Tug1-/-, and Tug1rescue mice (gating from WT peripheral blood shown). (C) Scatter dot plot (mean with standard error of the mean shown) of the number of pups at birth per copulatory plug for matings using male wild type, Tug1+/-; tg(Tug1); rtTA, Tug1-/-, or Tug1rescue (on dox diet) with wild type C57BL/6J females. Each dot represents a litter from a different mouse. (D) Sperm count from control (WT and Tug1+/-, n = 2), Tug1-/- (n = 2), and Tug1resuce (n = 3) mice. Each dot represents a different mouse and the error bars indicate the standard error of the mean. (E) Hematoxylin and eosin staining in Tug1+/-, Tug1-/-, and Tug1rescue testes and epididymis. (F) Morphological analysis of sperm from Tug1-/- (n = 2), and Tug1rescue (n = 3) mice. [file 13059_2020_2081_MOESM11_ESM.pdf]

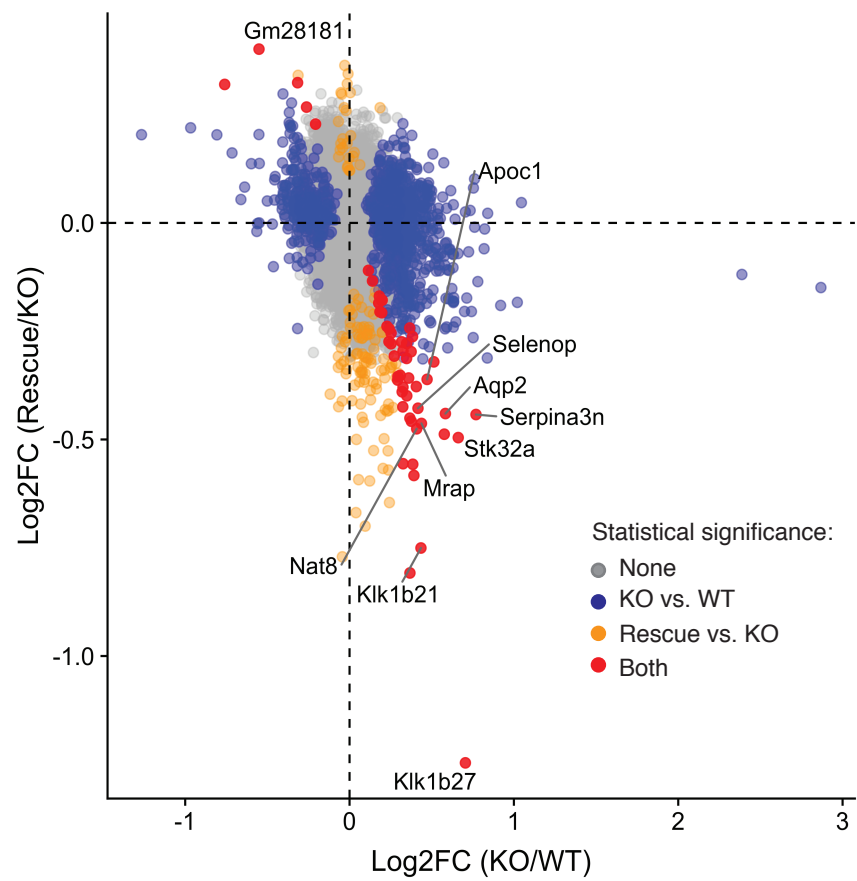

Supplement: Supplementary file 12 — Additional file 12: Fig. S7. Changes of gene expression in two comparisons of Tug1-/- (KO) vs. WT and Tug1rescue (Rescue) vs. Tug1-/- (KO). Each dot represents a gene whose x-axis value is the fold change of gene expression between KO and WT and y-axis value shows the fold change in Rescue vs. KO. Color describes statistical significance of fold change (adjusted p-value < 0.05): no statistical significance in either comparison (gray, N=33688); significance in KO vs. WT (blue, N=998); significance in Rescue vs. KO (orange, N=126); significance in both comparisons, KO vs. WT and Rescue vs. KO (red, N= 52). [file 13059_2020_2081_MOESM12_ESM.pdf]

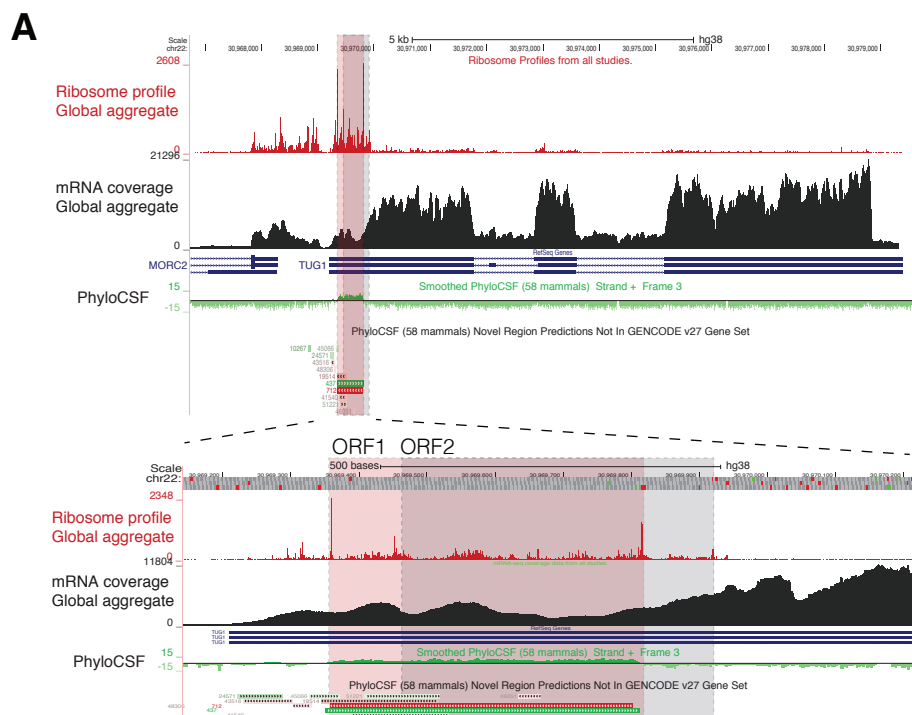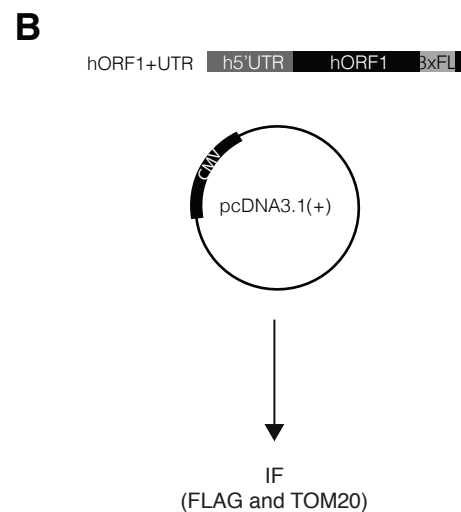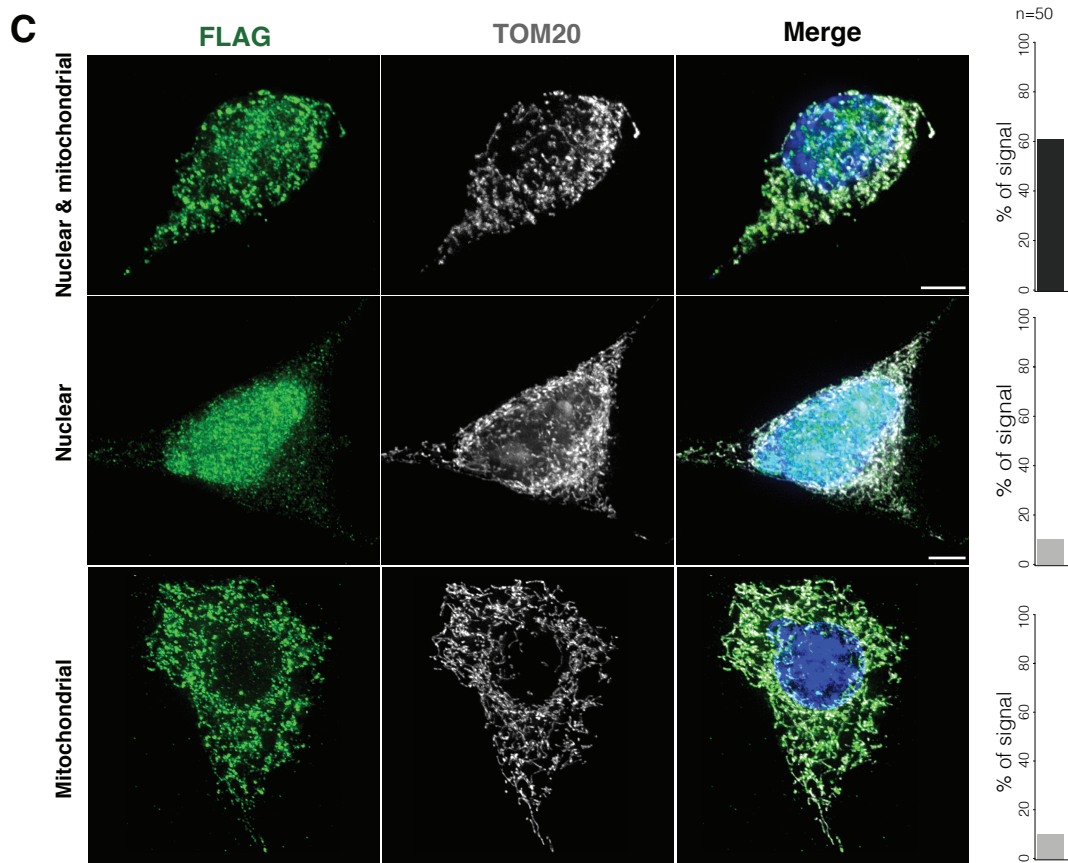

Supplement: Supplementary file 13 — Additional file 13: Fig. S8. The 5’ region of human TUG1 contains a conserved ORF. (A) GWIPS-viz tracks for human TUG1 genomic locus (hg38) is shown. Global aggregate of ribosome occupancy (ribosome profile), RNA-seq (mRNA coverage), and evolutionary protein-coding potential (PhyloCSF) across the TUG1 locus is shown. ORF1 and ORF2 are outlined with red and gray boxes, respectively. Tracks surrounding both ORFs are zoomed in for clarity (bottom). (B) Scheme showing human ORF1 construct design. hORF1 (labeled with a 3xFLAG epitope tag prior the stop codon) with the 5’UTR was inserted into pcDNA3.1(+) and transfected into HeLa cells. 48 hours post-transfection, TUG1-BOAT-3xFLAG localization was analyzed by immunofluorescence (IF) (shown in C). (C) Maximum intensity projection of HeLa cells expressing human 5’UTR-hORF1-3xFLAG. Localization of 3xFLAG tagged TUG1-BOAT was assessed by immunostaining against the 3xFLAG (green). Nucleus was monitored by DAPI (blue) and mitochondria was monitored by immunostaining against mitochondrial membrane translocase TOM20 (gray). Bar plot shows localization analysis of TUG1-BOAT. Scale bar is 5 μm. [file 13059_2020_2081_MOESM13_ESM.pdf]

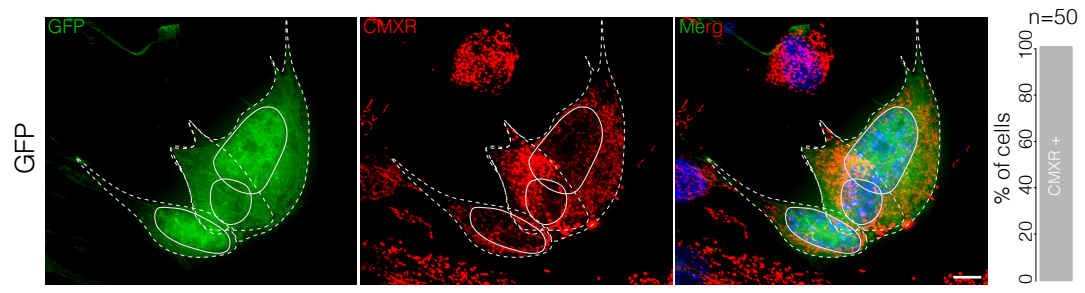

Supplement: Supplementary file 14 — Additional file 14: Fig. S9. GFP over-expression does not compromise mitochondrial membrane potential. Maximum intensity projections of z-stacks acquired 48 h post-transfection of 3T3 cells with GFP cloned into pcDNA3.1(+) under CMV promoter and staining with Chloromethyl-X-rosamine (CMXR). GFP (green) was used as control. CMXR is shown in red, DAPI in blue. On the right, quantification of cells expressing GFP and mitochondria membrane potential by CMXR (n = 50). Scale bar is 5 μm. [file 13059_2020_2081_MOESM14_ESM.pdf]

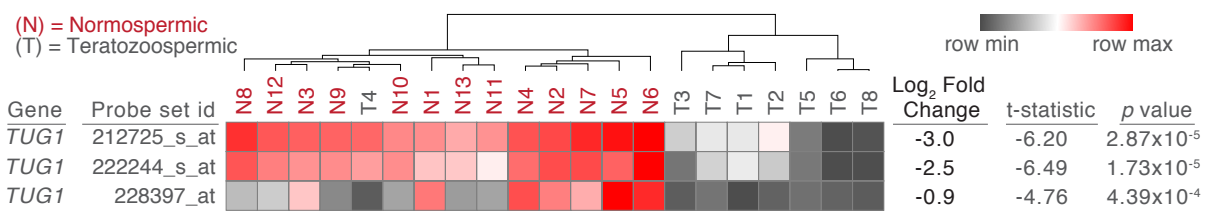

Supplement: Supplementary file 15 — Additional file 15: Fig. S10. Loss of TUG1 expression in infertile human males. Heatmap of microarray data from three different probe sets showing decreased expression of TUG1 in sperm from infertile teratozoospermic men (T) compared to fertile (normospermic) individuals (ND). In all cases, p < 4.39 x10^-4^. [file 13059_2020_2081_MOESM15_ESM.pdf]

**A. 3T3**

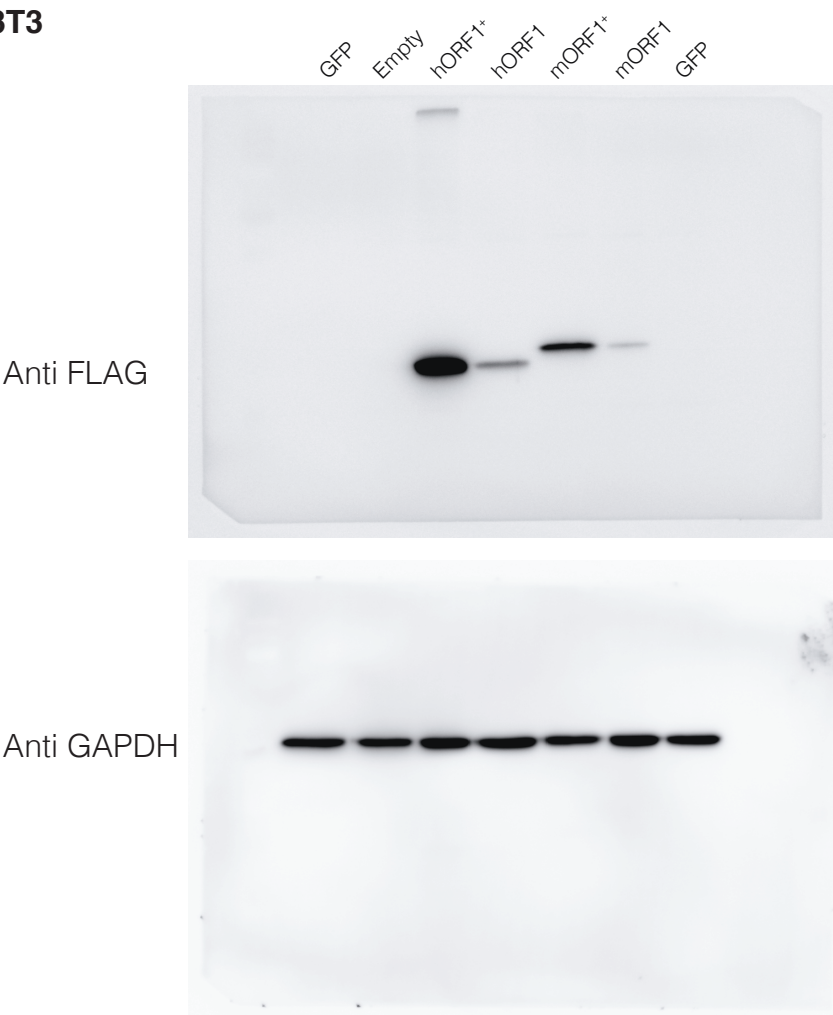

**B. HeLa**

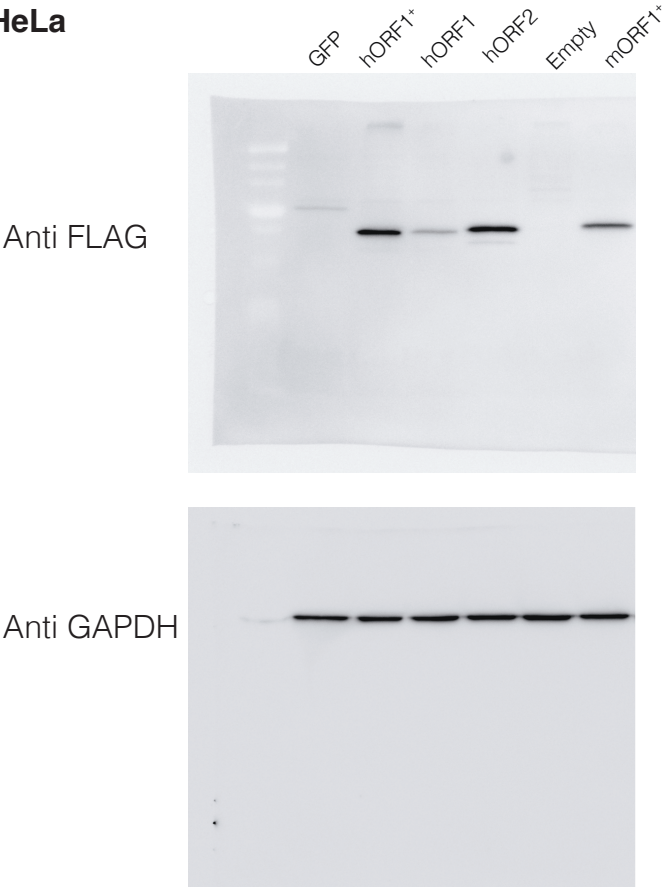

Supplement: Supplementary file 16 — Additional file 16: Fig. S11. Uncropped western blot images from Figure 5e. Original, uncropped images of western blots shown in Figure 5e. (A) Western blot of constructs overexpressed in 3T3 cells targeting the 3xFLAG tag (top). GAPDH is used as a loading control (bottom). (B) Western blot of constructs overexpressed in HeLa cells targeting the 3xFLAG tag (top). GAPDH is used as a loading control (bottom). [file 13059_2020_2081_MOESM16_ESM.pdf]
