# Supplementary material for: The Tug1 lncRNA locus is essential for male fertility
Source: Genome Biol. 2020 Sep 7;21:237. doi: 10.1186/s13059-020-02081-5 (PMC7487648; doi:10.1186/s13059-020-02081-5)
Supplement: Supplementary file 17 — Additional file 17. Review history. [file 13059_2020_2081_MOESM17_ESM.docx]

**Review history**

**First round of review**

**Reviewer #1**

The manuscript by Lewandowski et al. describes a follow-up work on the KO model of the Taurine Upregulated Gene 1 (Tug1) lncRNA, previously established and described studied by the Rinn lab. The authors provide a more detailed analysis of this highly conserved lncRNA locus by studying the consequences of the loss of the DNA locus and expression of a transgene in the mouse. They also describe for the first time a small protein produced from the TUG1 gene. Altogether the manuscript describes various efforts to elucidate which of the elements in the TUG1 locus have some regulatory effect or activity via mouse and cell-based models. The manuscript is well written and addresses important aspects of studying lncRNA function by dissecting the Tug1 locus by DNA, RNA and a short peptide generated by a short ORF. Overall it should be of interest to the lncRNA community, and potentially also to the fertility community. However, in a number of cases the authors conclusions are not backed up by the provided data. Overall, the topic of characterizing lncRNA function remains highly challenging, and after the experiments presented in this paper the story of Tug1 has more open than answered questions. Still, several experiments can help connect better the three different parts of the manuscript.
    
Major comments:
*     The DNA deletion result suggest that the Tug1 locus contains a cis-acting regulatory element. Is there any data on histone marks characteristic of enhancers (such as H3Kme1, H3k27ac) in the deleted region in any tissues? Is there any HiC data that links between the TUG1 locus to these dysregulated genes? Is TUG1 and the affected genes found in the same TAD? This should be addressed computationally, if possible, and discussed. The authors present some data on this Figure S1, but its limited to 3 mouse cell types, whereas ENCODE3 has data on >70 different tissues that can be used to try and address this.
*     The parts of the manuscript that describe the RNA-dependent function of TUG1 in regulating gene expression and those that describe TUG1-BOAT are currently completely disconnected. Can the authors connect the two by looking at changes in gene expression following expression of full-length TUG1, ORF-deleted TUG1 and ORF-only (in some cell system, e.g., Tug1-/- MEFs?) Or in some other way?

Minor comments:

*     Can the authors connect the dysregulated genes in Tug1-/- in-vivo model to male sterility? Do any of these genes have a known phenotype related to spermatogenesis? Do these genes have an effect on oligozoospermia and teratozoospermia?
*     In line 396 the authors state: "Collectively, these results provide evidence that human and mouse TUG1-BOAT have conserved roles in mitochondrial function". However, they don't show anything related to mitochondrial function of the peptide in a physiological expression setting, the statement therefore should be toned down.
*     Since the rescue construct used by the authors does not contain the full 5' region of TUG1, any phenotype that is not rescued could very well be due to the functionality of that RNA piece, that should be discussed in the paragraph that starts at line 314.
*     Base on Figure 1A it appears that the ORF is not actually part of the TUG1 sequence. The authors should show in 1A what they think, based on RNA-seq data, is the predominant transcript isoform that contains TUG1-BOAT.
*     Figure 6 and figure S6 are duplicated (seems like S6 is missing)
*     Figure 4E is very confusing. First, the Y-axis doesn't begin at 0. Second, the figure seems to compare TUG1 levels in the rescue relative to the KO, which should have basal levels, and does not give the context of the expression level in the WT cells. Overall, the claim of Tug1 rescue is very weak due to the low expression level of Tug1 in Tug -/- background.
*     Please improve the quality of Figure S5E.
*     Abstract: "Having identified a lncRNA loci" should be "Having identified a lncRNA locus"
*     Lines 100-100, "(cis)" and "(trans)" should be "(in cis)" and "(in trans)"
*     TUG1 introduction should also refer to the Rosenfeld lab study of TUG1 (PMD 22078878)
*     Line 154, PhyloCSF is a great tool for identifying protein-coding regions, I don't think it can be referred to as a tool for also identifying "non-coding regions".

**Reviewer #2**

The putative non-coding, almost ubiquitously expressed,  RNA TUG1 has been reported to be necessary for proper formation of photoreceptors in the eye, to have oncogenic properties and to have beneficial effects in ameliorating diabetes induced nerve damage through control of mitochondrial metabolism. In this report Lewandowski et al. associate TUG1 loss of function with male infertility using a whole body knockout.

My major concern is that this work, fails to clearly build on what was previously reported. It is unclear if this study will better clarify the function and mechanism of TUG1. Even from the most basic standpoint, it seems that importantly, the authors do not replicate the work that discovered TUG1: the authors report that "Tug1-/- mice are viable and do not display any obvious physiological abnormalities up to one year of age". Does this imply that there is no retinal phenotype? That that photoreceptors are well formed? More generally, as I will point out throughout this review, the authors cite, do not really to take into account what has been previously reported. This in my view greatly reduces the importance of this work in the context of building our understanding of what this loci may be responsible for, and more importantly, how it may be carrying out its function.

In part I they establish that male Tug1-/- mice are sterile. Sterility seems to result from a general increase in sperm defects. Not being a sperm expert I do not have much to comment about the data shown. Overall the authors present convincing data indicating that infertility of Tug1-/- mice is likely due to sperm developmental defects.
Minor comments in this section:

-The resolution Figure 1E is too low to evaluate the location of the FISH signal.
-The sperm images in the bottom of figure 2D are also of too low resolution
-The authors use RPKMs to quantify gene expression in figure 1 (figure 1C) but the more meaningful is TPM unity is used thereafter. The authors should convert the RPKM units into TPMs in figure 1C.

In part II the authors focus on the effect of the TUG1 deletion on genes in a 2MB neighborhood of TUG1 in both testes and other tissues. They find that 10 genes in Tug1's neighborhood have altered expression and that these genes are generally upregulated in KO. They also verify this in several tissues and in a hybrid cross. As such the data presented strongly supports a cis effect. There are however many questions left open: How does this locus regulates many genes simultaneously? To my knowledge there are no reports of a repressor that acts on several different targets simultaneously, this is a surprising, yet an underdeveloped result. Given the large amount of HiC data available, and the fact that the TUG1 is open in most tissues the authors could test whether TUG1 does come in proximity to the promoters of the affected genes. Similarly, given the large amount of ChIP data available for transcription factors, the authors could try to establish candidate factors that bind this locus. Alternatively, how do the authors hypothesize that this DNA element operate on its targets? Finally, are there human disease associated variants that impact the human ortholog of this putative regulatory element? More specifically, are there sterility specific associated variants within the human locus? Are there any of the affected genes associated with sperm production or maturation?

Minor comments on this section:

- Figure 3A uses log2 which is reasonable to show fold change, why do the authors use the natural logarithm in panel D?
- The Y scale in Fig3A should the same in all plots. Changing the scale for every tissue makes it very hard to gauge the effect of the KO in different tissues.

Part III concerns the TUG1 RNA function. Here the authors follow a beautiful approach to isolate the effect of the Tug1 RNA. They use a dox inducible transgene lacking the putative peptide inserted on the Tug1 KO background.

Unfortunately, the experiment only partially worked, the transgene has lower expression than the WT and even the heterozygous Tug1+/- individuals. This makes the rescue analysis challenging. Indeed figure S5 shows that there was no rescue of the phenotype, which, sure may require the RNA, DNA and the protein. However, establishing which components are critical to the phenotype would shed much needed light over how TUG1 functions. As presented, these data is confusing and not clearly interpretable. The negative result can be equally likely be because the TUG1 RNA does not play a role in the observed phenotype, because the expression of the RNA is too low, or because all three elements in the loci are necessary for proper sperm formation.

Finally, in (Long et al., 2016) the authors go to great lengths (including performing ChIRP) to show that TUG1 RNA functions by recognition of a AG rich DNA motif (figure 5C in that paper). The authors should report whether this motif is enriched on rescue targets. The trans-acting mechanism of TUG1, if such exists, is likely the same in the CNS and testis.

Specific comments on this section

Figure 4A shows a tree that seems the result of hierarchical clustering. The data underlying this tree should be shown. Specifically, a heatmap with all replicates and differentially expressed genes should be shown in figure 1 or in supplementary material, such heatmap would clearly show the cell type specificity of dysregulated genes. I venture that such heatmap would render figure 4B redundant while at the same time being more informative.

It is unclear why the authors performed GSEA on differentially expressed genes. Did they intended to write that they performed GSEA by sorting genes according to their fold change? DE test statistics? The method describing this analysis is not clear and neither is the explanation in the text. Did the DE analysis lack power to detect differences? Did the DE genes had no clear gene ontology enrichments?

It is unclear whether the two founder males had the tg(Tug1) transgene integrated in the same chromosomal loci. Differences in the integration target may result in unwanted variability.
I do not think it suffices to provide the data in supplementary material. Full data for the rescue, KO and WT testes should be shown. For example, a heatmap showing the most variable genes across all samples would help assess the variability of the data and the specificity of the effect of each genotype. Such data would also help put figure 4G in context. Figure 5 should label also the genes that are differentially expressed in each condition. There is no way to assess how many genes are DE in the rescue compared to WT that are not DE in the KO compared to WT. For this reason the statement that 51 genes are rescued is not informative.

The resolution and size of Figure S5B make it impossible to understand.

I find that the choice of using KO/WT in the X axis and rescue/KO in the Y axis of figure 4G to unnecessarily complicates the figure. The goal is to test whether the rescue is similar to a WT, hence correlation not anti-correlation of the differences between the WT and the rescue to the KO is the expected result if all works well. Why not always have the KO in the numerator (or denominator)?

Part IV concerns with the conserved open reading frame (ORF) within the 5' of the TUG1 loci.  I find this the least developed of all four parts. The authors convincingly show that one open reading frame in the 5' end of the TUG1 loci is conserved, and that its coding potential is well conserved across evolution, they name it TUG1-BOAT. This is a valuable point.  The authors, however fail to show that this protein is actually expressed in testes or at least that there exists at Tug1 isoform that has an unspliced 3' UTR that will lead to a stable, translationally capable mRNA. Indeed it is extremely likely that the three large introns upstream of the conserved ORF will doom the TUG1 mRNA to nonsense mediated decay, see for example (Lejeune and Maquat, 2005)). I find that it is critical to show that this protein actually gets translated or at least that there exists and isoform that results in a viable and stable mRNA encoding for this protein.

To understand the possible function of the TUG1-BOAT, the authors show that transfection of different TUG1-BOAT carrying constructs leads to detectable protein with specific localization patterns. They show that overexpression of TUG1-BOAT affects the mitochondria. I find this result hard to interpret because 1) The physiological levels of TUG1-BOAT are unknown and even if it is expressed, overexpressing at much higher levels than physiological levels may result in effects that do not represent the actual function of this protein. 2) It is unclear what this phenotype has anything to do with the sterility phenotype the authors set out to understand 3) It seems to contradict the beneficial mitochondria metabolic effects reported by (Long et al., 2016).


Lejeune, F., and Maquat, L.E. (2005). Mechanistic links between nonsense-mediated mRNA decay and pre-mRNA splicing in mammalian cells. Curr. Opin. Cell Biol. 17, 309-315.

Long, J., Badal, S.S., Ye, Z., Wang, Y., Ayanga, B.A., Galvan, D.L., Green, N.H., Chang, B.H., Overbeek, P.A., and Danesh, F.R. (2016). Long noncoding RNA Tug1 regulates mitochondrial bioenergetics in diabetic nephropathy. J. Clin. Invest. 126, 4205-4218.

**Authors’ response to reviewers**

**Second round of review**

**Reviewer #1**

The authors have put in a substantial effort in order to improve the manuscript, with some successes and some failures, but certainly no lack of trying... This has improved the manuscript as it now provides even more information about the biology of the Tug1 lncRNA in the mouse. The manuscript in present form should be of interest to the community and to the readership of Genome Biology.
One minor comment remaining- in Figure 4E, the significance of the difference between the "rescue" and "Tug1-/-" expression should be provided.

**Reviewer #2**

The authors have attempted different avenues to address initial concerns: 1) targeted mass spec to attempt

to identify the TUG1-BOAT protein, unfortunately the authors can’t convincingly show that this protein

is expressed endogenously. 2) The authors also found evidence for TUG1 low expression associated with

male infertility in humans. 3) The authors have also downstated their claims regarding the function of the

Tug1-BOAT protein.

In spite of these efforts, and as I mentioned in my original review, the work consists of four disjoint sections

that are very loosely tied together. The concern is that, although the authors do not explicitly claim it, the

work can be easily interpreted as showing that Tug1 has a new function and that a combination of a repressive

DNA element, a transacting non-coding RNA and a small protein are responsible for this phenotype. This,

however is not supported by data presented as I pointed out in my original review.

The authors report a very convincing sterility phenotype in Tug1-/- homozygous mice. They then present

three different mechanisms by which Tug1 may operate but that may or may not relate to the sterility

phenotype. The three different mechanisms are supported with varying degrees of strength.

**Tug1 acts in cis to repress some neighboring genes**. This is most convincingly supported by using

allele specific RNA-Seq expression in hybrid crosses. It remains unclear how this DNA element represses its

targets, the authors investigated some of the suggestions made in the previous version, but the mechanism by

which these repressive elements act across different cell types remains unknown and the connection between

this mechanism and the infertility phenotype is unclear.

**Tug1 acts in trans as a non-coding RNA**. I find this section unconvincing. The author’s argument

centers around a rescue system that, unfortunately, did not work. In RNA-Seq from testes that expresses

a compensatory RNA that excludes the 5’ open reading frame, the authors report that the expression of

a handful of genes (50) seems to recover. The result is confusing, is EVERY gene that is differentially

expressed when comparing KO vs rescue (just 50) show the same trend? Or is it that DE genes that are

*both* dysregulated in the KO vs WT *and* in the KO vs rescue show opposite trends? I was puzzled by this

assertion because the expression of the rescue construct is about 25 fold lower than the endogenous Tug1.

The authors seem to implicitly assume that there will be just a milder effect in the rescue because of its

lower expression. Given the much lower expression of the rescue, I find this hypothesis counterintuitive. I

downloaded data from GSE88819 as well as examined the Extended Data Table 2 and my interpretation is

not the same as that of the authors.

Figure 1 below shows a scatter plot for the testes data from GSE88819, with green marking significant (q-val

< 0.01 genes) and red marking the neighbors of Tug1. Results here are consistent with those reported in

Figure 3A of the manuscript.

**library**(scales)

data = **read.table**("GSE88819_Tug1_Tissues_Summary_diffData.tab", header = T,

row.names = 1)

tug1.neighbors = **c**("Rnf185", "8430429K09Rik", "Pla2g3", "Inpp5j", "Selm", "Smtn",

"Tug1", "Morc2a", "Osbp2")

data.test = data[data**$**sample_1 **==** "wt_testes" **&** data**$**sample_2 **==** "ko_testes",

]

data.test.sig = data.test[data.test**$**q_value **<** 0.05, ]

data.test.nosig = data.test[data.test**$**q_value **>=** 0.05, ]

1

**plot**(**log**(data.test.nosig**$**value_1 **+** 1), **log**(data.test.nosig**$**value_2 **+** 1), pch = 20,

col = **alpha**("darkblue", 0.2), xlab = "testes-WT", ylab = "testes-KO", bty = "n",

main = "GSE88819 testes Data")

**points**(**log**(data.test.sig**$**value_1 **+** 1), **log**(data.test.sig**$**value_2 **+** 1), pch = 20,

col = "darkgreen")

data.test.neighbors = data.test[data.test**$**gene_short_name **%in%** tug1.neighbors,

]

**points**(**log**(data.test.neighbors**$**value_1 **+** 1), **log**(data.test.neighbors**$**value_2 **+**

1), pch = 15, col = "darkred")

**text**(**log**(data.test.neighbors**$**value_1 **+** 1), **log**(data.test.neighbors**$**value_2 **+**

1), labels = data.test.neighbors**$**gene_short_name, pos = 4)

**GSE88819 testes Data**

The authors provided the comparison to the rescue experiment in the extended data table 2, in my view,

there some kind of artifact that resulted in a number of genes having lower expression in the KO and rescue

data sets compared to the WT data (Figures below).

rescue.data = **read.table**("Extended_Data_Table_2_Tug1_TissuesTestes_ExtData.csv",

header = TRUE, sep = ",")

\## Gene name is not an ideal matching column, however, gene IDs change

\## between the data sets. May miss some of the significant hits of prior

\## data.

test.orig.sig = data.test.sig**$**gene_name

rescue.data.neighbors = rescue.data[rescue.data**$**gene_name **%in%** tug1.neighbors,

]

**plot**(**log**(rescue.data**$**tpmMeanWT **+** 1), **log**(rescue.data**$**tpmMeanKO **+** 1), pch = 20,

col = **alpha**("darkblue", 0.2), xlab = "WT", ylab = "KO", bty = "n")

**points**(**log**(rescue.data[rescue.data**$**gene_name **%in%** test.orig.sig, "tpmMeanWT"] **+**

1), **log**(rescue.data[rescue.data**$**gene_name **%in%** test.orig.sig, "tpmMeanKO"] **+**

1), col = "red", pch = 15)

**text**(**log**(rescue.data.neighbors**$**tpmMeanWT **+** 1), **log**(rescue.data.neighbors**$**tpmMeanKO **+**

1), labels = rescue.data.neighbors**$**gene_name, pos = 4, cex = 0.75)

**plot**(**log**(rescue.data**$**tpmMeanWT **+** 1), **log**(rescue.data**$**tpmMeanRescue **+** 1), pch = 20,

col = **alpha**("darkblue", 0.2), xlab = "WT", ylab = "Resc", bty = "n")

**points**(**log**(rescue.data[rescue.data**$**gene_name **%in%** test.orig.sig, "tpmMeanWT"] **+**

1), **log**(rescue.data[rescue.data**$**gene_name **%in%** test.orig.sig, "tpmMeanRescue"] **+**

1), col = "red", pch = 15)

**text**(**log**(rescue.data.neighbors**$**tpmMeanWT **+** 1), **log**(rescue.data.neighbors**$**tpmMeanRescue **+**

1), labels = rescue.data.neighbors**$**gene_name, pos = 4, cex = 0.75)

**plot**(**log**(rescue.data**$**tpmMeanKO **+** 1), **log**(rescue.data**$**tpmMeanRescue **+** 1), pch = 20,

col = **alpha**("darkblue", 0.2), xlab = "KO", ylab = "Resc", bty = "n")

**points**(**log**(rescue.data[rescue.data**$**gene_name **%in%** test.orig.sig, "tpmMeanKO"] **+**

1), **log**(rescue.data[rescue.data**$**gene_name **%in%** test.orig.sig, "tpmMeanRescue"] **+**

1), col = "red", pch = 15)

**text**(**log**(rescue.data.neighbors**$**tpmMeanKO **+** 1), **log**(rescue.data.neighbors**$**tpmMeanRescue **+**

1), labels = rescue.data.neighbors**$**gene_name, pos = 4, cex = 0.75)

I am providing the small analysis I used to re-examine the data provided for the authors to reproduce and, if

they do not agree, rebut the analysis. However, from this analysis it seems to me that the authors conclusion

may stem from differences that have to do less with the biology of testes and more with library construction

or sample acquisition variability.

**Tug1 encodes a conserved protein that affects mitochondrial processes** Here the authors did not

address the original concerns. It is still unclear whether the protein is actually expressed and it is also unclear

how to interpret the result of overexpression of the protein at non-physiological levels. The authors have

qualified their assertions and perhaps this suffices to prevent readers from misinterpreting the results presented.

However, I do not see how their experiments help establish the potential function of the TUG1-BOAT protein

**Authors’ response to reviewers**

We thank the reviewers for their assessment of our manuscript “***The Tug1 lncRNA locus is essential for male***

***fertility***”. Their comments and insightful suggestions were helpful in adding additional lines of evidence and

helped bolster and better contextualize our results and conclusions. We believe that our manuscript has

improved significantly. In our revision work we: 1) present data to show that a transcript containing TUG1-BOAT

is stably detected in the testes; 2) performed several experiments in which we try to address the endogenous

detection of TUG1-BOAT (via deriving *Tug1* mouse embryonic stem cells, generation of antibodies, as well as

targeted mass spectrometry) and 3) clarify the text throughout the manuscript to tone down the language with

respect to the endogenous detection and role of TUG1-BOAT. Please find below a point-by-point response (text

in blue) to each reviewers' comments.

Reviewer reports:

**Reviewer #1:**

The manuscript by Lewandowski et al. describes a follow-up work on the KO model of the Taurine Upregulated

Gene 1 (Tug1) lncRNA, previously established and described studied by the Rinn lab. The authors provide a

more detailed analysis of this highly conserved lncRNA locus by studying the consequences of the loss of the

DNA locus and expression of a transgene in the mouse. They also describe for the first time a small protein

produced from the TUG1 gene. Altogether the manuscript describes various efforts to elucidate which of the

elements in the TUG1 locus have some regulatory effect or activity via mouse and cell-based models. The

manuscript is well written and addresses important aspects of studying lncRNA function by dissecting the Tug1

locus by DNA, RNA and a short peptide generated by a short ORF. Overall it should be of interest to the lncRNA

community, and potentially also to the fertility community. However, in a number of cases the authors conclusions

are not backed up by the provided data. Overall, the topic of characterizing lncRNA function remains highly

challenging, and after the experiments presented in this paper the story of Tug1 has more open than answered

questions. Still, several experiments can help connect better the three different parts of the manuscript.

Major comments:

**R1.1.** The DNA deletion result suggest that the Tug1 locus contains a cis-acting regulatory element. Is there any

data on histone marks characteristic of enhancers (such as H3Kme1, H3k27ac) in the deleted region in any

tissues?

In two previous studies we have investigated this question using different approaches. First, we scanned for any *cis* regulatory enhancer activity across the entire *Tug1* locus and promoter (as well as many others lncRNA loci)

using the Massively Parallel Reporter Assay (MPRA) (PMID: 30537984). We found strong signal in the *Tug1*

promoter region but not in the gene-body (whereas other lncRNA loci do have strong “enhancer” peaks in their

gene-bodies (PMID: 30537984)).

In a second study, (PMID: 27927715) we comprehensively and systematically characterized the histone marks,

chromatin binding proteins, and transcription factors associated with lncRNAs relative to mRNAs. *Tug1* was

strikingly highly enriched in H3K9me3 across the gene-body while also being expressed at relatively moderate

to high levels. Thus, *Tug1* is highly associated with heterochromatic features in the gene-body and not with signs of any enhancer activity (e.g. H3K4me1) (**Reviewer Figure 1**). Finally, we note that *Tug1* KO results in the upregulation of neighboring genes, thus the regulatory activity in the DNA represents an interesting and much less common “suppressor element.”

We thank the reviewer for this comment and have discussed these studies in the context of histone marks and

DNA regulatory elements:

**(lines 431-437)** *“Consistent with a repressive modality of the Tug1 locus, a recent study which*

*systematically tested for enhancer activity across lncRNA loci, reported a lack of enhancer activity across*

*the Tug1 gene body, but found enhancer activity at all other lncRNA loci examined (Groff et al., 2018).*

*Contrary to enhancers, only a handful of repressor elements and silencers have been identified and*

*characterized in mammalian genomes (Li and Arnosti, 2011; Li et al., 2014; Qi et al., 2015; Tan et al.,*

*2010). Further defining the precise DNA repressive elements as well as their mechanism will be of interest*

*to understand the regulatory principles of DNA elements within the Tug1 locus.”*

**Reviewer Figure 1.** ENCODE ChIP-seq tracks from UCSC Genome Browser for H3K4me1 (blue)

and H3K27ac (purple) at the *Tug1* locus in various tissues (testis, mESC, cortex, heart, brown

adipose tissue, macrophages, and bone marrow). ChIP peaks for H3K27ac are present at the Tug1

promoter, but not within the gene body.

**R1.2.** Is there any HiC data that links between the TUG1 locus to these dysregulated genes? Is TUG1 and the

affected genes found in the same TAD? This should be addressed computationally, if possible, and discussed.

The authors present some data on this Figure S1, but its limited to 3 mouse cell types, whereas ENCODE3 has

data on >70 different tissues that can be used to try and address this.

We thank the reviewer for this suggestion and have now analyzed Hi-C datasets in embryonic stem cells from

human and mouse using the Yue Lab’s 3D Genome Browser. We found that the genes neighboring the *Tug1*

locus that are dysregulated upon deletion of the *Tug1* locus are within the same TAD in both human and mouse

stem cells (**Reviewer Figure 2**). Further, we have also examined Capture Hi-C data in an immune cell type and

found that the *Tug1* locus interacts with the local genes that are found dysregulated (**Reviewer Figure 3**). We

have added new text to comment on the TAD in the text:

**(lines 229-230)** *“Notably, these genes are downstream of the Tug1 TSS and located within the same*

*topological associated domain (TAD) in embryonic stem cells (Dixon et al., 2015, 2012).”*

**Reviewer Figure 2.** *Tug1* and *cis* upregulated genes reside within the same Topologically Associated Domain (TAD) in

human and mouse ES cells. Hi-C interaction heat maps in mouse ES cells (A) and H1 ES cells (B) showing the degree

of physical interaction defined by Hi-C analysis for genomic region pairs from a 3Mb region of mouse chromosome 11

(mm9) and human chromosome 22 (hg19). Tug1 TAD domain in mES spans through coordinates: chr11:3000000-

4960000, and in human ES cells chr22:31200000-31960000 (Dixon et al., 2015).

**Reviewer Figure 3.** Capture HiC data (ChICP) in human megakaryocytes showing

that human TUG1 locus interacts with the downstream gene SMTN. Data obtained

from chicp.org.

**R1.3**. The parts of the manuscript that describe the RNA-dependent function of TUG1 in regulating gene

expression and those that describe TUG1-BOAT are currently completely disconnected. Can the authors

connect the two by looking at changes in gene expression following expression of full-length TUG1, ORF-deleted

TUG1 and ORF-only (in some cell system, e.g., Tug1-/- MEFs?) Or in some other way?

We agree with the reviewer that further exploring if any of the modalities at the *Tug1* locus have functional

overlap, synergy, or are completely independent could help understand to what extent the *Tug1* lncRNA and

TUG1-BOAT protein have overlapping and/or independent roles on gene expression.

To address this, we followed the reviewer suggestion and used a cell culture-based model in combination with

overexpression of the *Tug1* lncRNA and TUG1-BOAT peptide. We derived and established *Tug1* WT and *Tug1*

KO mouse embryonic stem cell lines from *Tug1* mice. We then transfected either a control vector, the *Tug1*

lncRNA lacking ORF1, or TUG1-BOAT in the *Tug1* KO background. We performed RNA-sequencing and

differential gene expression analyses for *Tug1* WT, *Tug1* KO, *Tug1* KO + *Tug1* RNA (no ORF1) and *Tug1* KO +

mUTR_ORF1. We found that there is not much difference in gene expression changes between RNA

overexpression and TUG1-BOAT overexpression, as the changes are highly correlated in the two experiments

**(Reviewer Figure 4, below**). However, TUG1-BOAT was not efficiently translated in the mES system, as we

could not achieve sufficiently detectable levels of the protein by Western blot. We then tested different promoters,

but translation level of TUG1-BOAT was not improved. Although this result might indicate that *Tug1* RNA and

TUG1-BOAT affect gene expression in a similar way, due to low translation of TUG1-BOAT in the mES system,

we would caution to make that conclusion at this point. We believe that generating additional genetic models --

where this question can be addressed in a relevant tissue such as the testes – would be the best path forward.

This will require many more experiments and careful considerations, which will be part of follow up studies.

**Reviewer Figure 3.** Capture HiC data (ChICP) in human megakaryocytes showing

that human TUG1 locus interacts with the downstream gene SMTN. Data obtained

from chicp.org.

However, we feel that addressing this particular question, although important, is outside the scope of the current

study.

**Reviewer Figure 4. Comparison of RNA and peptide in terms of gene expression in *Tug1* KO background.** Each dot represents a gene. X-axis: log fold change of gene expression in RNA-plasmid

relative to empty plasmid. Y-axis: log fold change of gene expression between ORF-plasmid and empty control. Log fold change was calculated by DESeq2.

However, we feel that addressing this particular question, although important, is outside the scope of the current study.

Minor comments:

**R1.4.** Can the authors connect the dysregulated genes in Tug1-/- in-vivo model to male sterility? Do any of these genes have a known phenotype related to spermatogenesis? Do these genes have an effect on oligozoospermia and teratozoospermia?

We agree with the reviewer that providing more information on what is known about *Tug1* and the dysregulated

genes in the *Tug1* KO model could help better contextualize our findings. Using previously published gene

expression data, we found that *Tug1* RNA is significantly downregulated in the sperm of sterile men

(teratozoopsermia) compared to fertile men (Platts et al. 2007). We have included these results in a new

**Extended Data Figure 7 (shown below)** and have discussed this new data in the **Discussion Section:**

**(lines 406-411)** *“Notably, there is some evidence that Tug1/TUG1 may have a conserved role in male*

*fertility in humans. Microarray profiling of sperm from sterile men (a condition called teratozoospermia)*

*have significantly less TUG1 expression compared with sperm from fertile males (Extended Data Fig. 7)*

*(Platts et al., 2007). Thus, we speculate that the Tug1-/- mouse model used in this study could potentially*

*be of value to further investigate the functions of Tug1/TUG1 with relevance to human male fertility.”*

**Extended Data Figure 7. Loss of TUG1 expression in infertile human males.** Heatmap of

microarray data from three different probe sets showing decreased expression of TUG1 in sperm

from infertile teratozoospermic men compared to fertile individuals (normospermic) (in all cases

p < 4.39 x10-4 ).

In addition, we had previously searched the literature and noted that some of the genes dysregulated in *Tug1*

KO testes are associated with male sterility phenotypes:

**(lines 483-491)** *“There is some evidence that the cis genes dysregulated upon deletion of the Tug1 locus*

*have a role in male fertility. Loss-of-function mutations in 2 of the 6 cis genes upregulated in Tug1-/- testes,*

*Smtn and Pla2g3, are characterized to have male fertility and sperm maturation defects (Niessen et al.,*

*2005; Sato et al., 2010). However, the impact of these genes on male fertility in an overexpression context*

*have not been reported. A role in male fertility for the other four upregulated cis genes upon deletion of*

*Tug1 (Gm11946, Rnf185, Selm, and 8430429K09Rik) have not yet been reported. In addition, there is*

*some evidence for a potential role of Tug1 RNA in male fertility. Mice with a loss-of-function mutation for*

*Selenop, a gene that was found significantly upregulated in Tug1-/- testes and significantly and*

*reciprocally regulated in Tug1rescue testes, is reported to have reduced male fertility (Hill et al., 2003).“*

**R1.5**. In line 396 the authors state: "Collectively, these results provide evidence that human and mouse TUG1-

BOAT have conserved roles in mitochondrial function". However, they don't show anything related to

mitochondrial function of the peptide in a physiological expression setting, the statement therefore should be

toned down.

We thank the reviewer for pointing this out, as we did not intend to state that we have identified that TUG1-BOAT

has a normal physiological role in mitochondrial function. The conclusion we intended to make in this manuscript

is that there is a conserved ORF in the *Tug1* locus and that when this ORF is overexpressed, a stable protein is

produced and impacts mitochondrial membrane potential. We do agree with the reviewer that instances in the

text surrounding the endogenous detection/function of TUG1-BOAT were unclear and/or overstated. We have

corrected the language throughout the manuscript:

**(lines 465-467)** *“Identifying endogenous and disease contexts in which Tug1 RNA and TUG1-BOAT are*

*detected and function endogenously will be important to further explore the potential link between Tug1*

*and the mitochondria, and determine whether the locus does contain a third coding function.”*

**(lines 491-494)** *“As for a role for the putative protein, TUG1-BOAT, there is currently limited evidence for*

*its endogenous existence. Thus, future work clarifying an endogenous presence and function for TUG1-*

*BOAT will be necessary to understand whether it contributes to the male fertility defect.”*

**Extended Data Figure 7. Loss of TUG1 expression in infertile human males.** Heatmap of

microarray data from three different probe sets showing decreased expression of TUG1 in sperm

from infertile teratozoospermic men compared to fertile individuals (normospermic) (in all cases

p < 4.39 x10-4 ).

**R1.6**. Since the rescue construct used by the authors does not contain the full 5' region of TUG1, any phenotype

that is not rescued could very well be due to the functionality of that RNA piece, that should be discussed in the

paragraph that starts at line 314.

We agree with the reviewer that in addition to the low levels of transgenic *Tug1* RNA induction, different isoforms

may also play a role. We have commented on this in the **Results Section**:

**(lines 317-319)** *“While this finding may suggest that the sterility phenotype is not due to the lncRNA*

*transcript, we note that the lack of a fertility rescue may also be due to the low levels of Tug1 expression*

*from the transgene in the testes or because a different Tug1 RNA isoform is required.”*

**R1.7**. Base on Figure 1A it appears that the ORF is not actually part of the TUG1 sequence. The authors should

show in 1A what they think, based on RNA-seq data, is the predominant transcript isoform that contains TUG1-

BOAT.

We examined our PolyA+ RNA-seq data and find a low number of reads in the 5’ region of *Tug1*. In addition, a

recent study that identified a conserved ORF in the 5’ region of human TUG1 also found a low number of reads

by poly(A)+ RNA-seq (PMID: 31155234). However, there is evidence to support that the ORF is contained within

a transcript. One piece of evidence comes from Ensembl, where the *Tug1* locus includes a transcript annotation

(Tug1-205, ENSMUST00000193809.1) that contains the entire ORF region. The second line of evidence comes

from a new experiment we have added to the manuscript. We isolated RNA from WT and Tug1-/- testes and

performed RT-PCR using random hexamers and oligo(dT). In this experiment we were able to amplify the 5’

region containing the ORF in mouse testis. We did not detect this band in samples where the RT was omitted

(No RT) or in the *Tug1* KO sample, indicating that the amplicons comes from the RNA. We have added this new

data to **Figure 5C (shown below)** and have added the following text to the **Results Section:**

**(lines 345-352)** *“To determine if a transcript containing ORF1 (located in the 5’ region) is expressed in*

*testes, we performed reverse transcriptase PCR (RT-PCR) on wild type and Tug1-/- testes. Using a primer*

*set that spans ORF1, we detected a band at approximately 700 bp in the wild type sample with RT*

*enzyme and did not detect a product in the no RT enzyme or in the Tug1-/- control samples (Fig. 5C). As*

*a loading control, 7SK, an abundant snRNA (Gurney and Eliceiri, 1980; Wassarman and Steitz, 1991),*

*is detected in both wild type and Tug1-/- samples (Fig. 5C). Taken together, these results provide evidence*

*that the 5’ region containing ORF1 is expressed in testes and presents the possibility that this ORF could*

*generate the predicted protein.”*

**Figure 5: (C)** RT-PCR in wild type and *Tug1*-/- testes for

ORF1. Schematization of Tug1 locus showing location of

primers (triangles) and ORF1 (red rectangle).

In addition, we sought to further investigate if there is evidence that *Tug1* undergoes nonsense-mediated mRNA

decay (NMD). Two separate studies that either genetically remove (mouse testis) or knockdown NMD pathway

components (human cell line) did not find evidence that *Tug1* undergoes NMD (PMID: 27149259, PMID:

22369889). This suggests that *Tug1* produces a transcript that contains TUG1-BOAT and from at least two

separate studies, there is no evidence that *Tug1* undergoes NMD.

**R1.8**. Figure 6 and figure S6 are duplicated (seems like S6 is missing)

We thank the reviewer for catching this error. We overlooked this in the submission process. The revised

submission includes the correct Figure S6.

**R1.9**. Figure 4E is very confusing. First, the Y-axis doesn't begin at 0. Second, the figure seems to compare

TUG1 levels in the rescue relative to the KO, which should have basal levels, and does not give the context of

the expression level in the WT cells. Overall, the claim of Tug1 rescue is very weak due to the low expression

level of Tug1 in Tug -/- background.

We have replaced panel E in figure 4 with a new panel showing *Tug1* expression (TPM) in

the testes from wildtype (n=4), *Tug1* knockout (n=4) , and *Tug1*rescue mice (n=3) (**this new**

**Fig. 4E panel is shown to the right**). Indeed, the induction of transgenic *Tug1* RNA is low

in the testes by RNA-seq (**Fig. 4E**) and by qRT-PCR (**Extended Data Fig. 5A**). Despite the

low transgenic expression of *Tug1* in the testes, we would like to note that a number of

lncRNAs have been shown to function at low levels and we mention this in the **Discussion**

**Section**:

**(lines 438-449)** *“In addition to a DNA regulatory modality, this study finds evidence*

*for an RNA-based role for Tug1 in the testes by a trans mechanism. In support of*

*this conclusion, we found that a subset of genes dysregulated in Tug1-/- testes could*

*be rescued by ectopic expression of Tug1 RNA, even at low levels. While the Tug1*

*transgene was expressed at lower levels than wild type Tug1 RNA, other lncRNAs*

*such as Hottip and Xist have been shown to exert a biological activity at relatively*

*low copy numbers (Sunwoo et al., 2015; Wang et al., 2011), thus Tug1 RNA appears*

*to have functional activity even at low levels. Consistent with our finding of a transacting*

*role for Tug1 RNA on gene expression, a previous study found that Tug1 RNA*

*can regulate the levels of Ppargc1a mRNA in cultured podocytes (Long et al., 2016). In our RNA-seq*

*dataset of 8 different tissues, Ppargc1a was not significantly dysregulated, but it is important to note that*

*our dataset does not include the kidney. We also note that low levels of Tug1 transgene RNA was not*

*sufficient to rescue the Tug1 male fertility defect. More studies will be needed to determine the indirect mechanism(s) of Tug1 RNA on gene expression in a male germ cell context in vivo.”*

**Figure 4E:** New panel E showing WT expression of *Tug1* in mouse testes.

**R1.10**. Please improve the quality of Figure S5E.

We have produced all figures at high resolution and have submitted these as such. It appears that during the

conversion of the image at submission, the image quality was reduced. We will raise this issue with Genome

Biology.

**R1.11.** Abstract: "Having identified a lncRNA loci" should be "Having identified a lncRNA locus"

Thank you for pointing out this error. We have made the suggested correction.

**R1.12**. Lines 100-100, "(cis)" and "(trans)" should be "(in cis)" and "(in trans)"

Thank you for pointing out this error. We have made the suggested correction:

**R1.13.** TUG1 introduction should also refer to the Rosenfeld lab study of TUG1 (PMD 22078878)

This was an oversight on our part. We have now included the reference:

**(lines 111-115)** *“A number of studies have found evidence suggesting diverse RNA-based roles for Tug1,*

*including but not limited to the development of photoreceptors (Young et al., 2005) and in regulating gene*

*expression in the nucleus by associating with the polycomb repressive complex 2 (PRC2) (He et al.,*

*2018; Khalil et al., 2009; Long et al., 2016; Yang et al., 2011).”*

**R1.14**. Line 154, PhyloCSF is a great tool for identifying protein-coding regions, I don't think it can be referred to as a tool for also identifying "non-coding regions".

We agree with the reviewer and have changed the text to more accurately reflect that PhyloCSF is a tool that

can distinguish protein-coding and noncoding regions, as stated in the original publication (PMID: 21685081).

Our intention here was to indicate that PhyloCSF identifies protein-coding regions, but the lack of a positive score suggests that a transcript has low or no coding potential. It can thus be useful in assessing whether a transcript is more likely to be coding or noncoding.

**(lines 148-151)** *“Further conservation analyses lead us to identify a highly conserved putative open*

*reading frame (ORF) in the Tug1 locus, as indicated by phylogenic codon substitution frequencies*

*(PhyloCSF) (Fig. 1A), a computational tool for identifying protein-coding and non-coding regions (Lin et*

*al., 2011).”*

**Reviewer #2:**

The putative non-coding, almost ubiquitously expressed, RNA TUG1 has been reported to be necessary for

proper formation of photoreceptors in the eye, to have oncogenic properties and to have beneficial effects in

ameliorating diabetes induced nerve damage through control of mitochondrial metabolism. In this report

Lewandowski et al. associate TUG1 loss of function with male infertility using a whole body knockout.

**R2.1**. My major concern is that this work, fails to clearly build on what was previously reported. It is unclear if

this study will better clarify the function and mechanism of TUG1. Even from the most basic standpoint, it seems that importantly, the authors do not replicate the work that discovered TUG1: the authors report that "Tug1-/- mice are viable and do not display any obvious physiological abnormalities up to one year of age". Does this imply that there is no retinal phenotype? That that photoreceptors are well formed? More generally, as I will point out throughout this review, the authors cite, do not really to take into account what has been previously reported. This in my view greatly reduces the importance of this work in the context of building our understanding of what this loci may be responsible for, and more importantly, how it may be carrying out its function.

We agree that many studies have identified a number of promising roles for *Tug1*, which of themselves are

interesting and certainly warrant further investigation. However, inquiries into these areas are outside the scope of this study. In our original report of 20 lncRNA knockout strains, in which we found that "*Tug1*-/- mice are viable and do not display any obvious physiological abnormalities up to one year of age", we did not specifically analyse retinal phenotypes (PMID: 24381249). We selected to perform a more detailed analysis of *Tug1 in vivo* with respect to male fertility because relatively quickly we identified a penetrant male sterility phenotype in *Tug1* knockout mice. Moreover, we also learned that *TUG1* RNA is downregulated in sterile males, thus linking the locus to a relevant human disease for this mouse model. Owing to the many other possible aspects of biology with respect to Tug1 we may have missed, we would not want to confound this study by distracting from the very clear phenotype of male sterility in which the context of the study is framed. Much more work will be required to fully characterize other potential defects these mice may display.

In order to understand how the *Tug1* locus could be mediating function, we wanted to take a step back and

evaluate what molecular properties were present at the *Tug1* locus in order to identify how this locus could be

functioning. This was important because a number of studies investigating lncRNA loci have found that they can function by a variety of mechanisms including DNA regulatory elements, the RNA, the act of transcription, and even mis-annotated proteins. Indeed, this study identifies an unexpected complexity with respect to the number of molecular modalities present at the *Tug1* locus. We believe that this locus is complex and will require many more studies to untangle the molecular modes of action. Indeed, the complexity of the *Tug1* locus appears similar to the human D4Z4 locus, a locus found deleted in human disease and that has been studied for many decades which has uncovered a DNA, RNA, and ectopic protein modalities (discussed below in R2.6). Here, we have uncovered these different layers and laid the foundation for future work to fully untangle Tug1's molecular modes of action in the context of a male sterility phenotype that has not been reported.

**R2.2**. In part I they establish that male Tug1-/- mice are sterile. Sterility seems to result from a general increase

in sperm defects. Not being a sperm expert I do not have much to comment about the data shown. Overall the

authors present convincing data indicating that infertility of Tug1-/- mice is likely due to sperm developmental

defects.

We thank the reviewer for this comment.

Minor comments in this section:

**R2.3**. The resolution Figure 1E is too low to evaluate the location of the FISH signal.

We have produced all figures at high resolution and have submitted these as such. It appears that during the

conversion of the image at submission, the image quality was reduced. We will raise this issue with Genome

Biology. In addition, to make the nuclear-cytoplasm border more visible, in the revised manuscript we have

outlined the nucleus with a dashed line.

**R2.4**. The sperm images in the bottom of figure 2D are also of too low resolution

We have produced all figures at high resolution and have submitted these as such. It appears that during the

conversion of the image at submission, the image quality was reduced. We will raise this issue with Genome

Biology.

**R2.5**. The authors use RPKMs to quantify gene expression in figure 1 (figure 1C) but the more meaningful is

TPM unity is used thereafter. The authors should convert the RPKM units into TPMs in figure 1C.

In this figure we plotted publicly available gene expression data generated by GTEX, which uses RPKM in their

analysis pipeline.

**R2.6**. In part II the authors focus on the effect of the TUG1 deletion on genes in a 2MB neighborhood of TUG1

in both testes and other tissues. They find that 10 genes in Tug1's neighborhood have altered expression and

that these genes are generally upregulated in KO. They also verify this in several tissues and in a hybrid cross.

As such the data presented strongly supports a cis effect. There are however many questions left open: How

does this locus regulates many genes simultaneously? To my knowledge there are no reports of a repressor that

acts on several different targets simultaneously, this is a surprising, yet an underdeveloped result. Given the

large amount of HiC data available, and the fact that the TUG1 is open in most tissues the authors could test

whether TUG1 does come in proximity to the promoters of the affected genes.

We agree with the reviewer that the *cis*-repressor function of the *Tug1* locus at several different targets is a

surprising result and that this could be better contextualized. We were also curious if there were other repressor

elements that have been reported to act on multiple genes locally. Interestingly, in our search we identified a

repeat array consisting from D4Z4 macrosatellites, that has a reduction in copy number in patients with

facioscapulohumeral dystrophy (FSHD) (PMID: 21149563). Mechanistic studies of the D4Z4 locus that span

from the 1990’s until the present identified that each repeat unit contains a *cis*-repressor element (among other

molecular properties), thus when repeat number is reduced, local repressive chromatin is lost leading to

transcriptional upregulation of proximally located genes (PMID: 21149563, PMID: 15520407, PMID: 19593370,

PMID: 19359275). We comment on some of the similarities between D4Z4 and Tug1 in the Discussion Section:

**(lines 471-480)** *“There are few biological contexts in the literature describing noncoding loci implicated*

*in human disease with potential DNA, RNA, and protein functionalities. However, one intriguing example*

*is the human D4Z4 locus, which is a repeat region that is reduced in copy number in humans with*

*facioscapulohumeral dystrophy (FSHD) (Lemmers et al., 2007; Wijmenga et al., 1992). Interestingly, in*

*the disease context where the repeat number is reduced, local repressive chromatin is lost leading to*

*transcriptional upregulation of proximally located genes (Gabellini et al., 2002; Rijkers et al., 2004).*

*Mechanistic studies spanning decades have identified a number of candidate molecular modalities at the*

*D4Z4 locus, including a lncRNA, a peptide that is only detected in the disease context, and a DNA*

*repressor element (Cabianca and Gabellini, 2010; Dumbovic et al., 2017; Gabellini et al., 2002; Rijkers*

*et al., 2004; Snider et al., 2009; Zeng et al., 2009).”*

To address the reviewers second comment, we searched publicly accessible HiC data portal and found that

genes dysregulated in *Tug1* KO tissues are within the same TAD (**Reviewer Figure 2**). Further, we examined

capture HiC data from https://www.chicp.org (which largely contains datasets from immune cell populations) and found that the Tug1 locus makes contacts with neighboring genes that are found dysregulated in our *Tug1* KO tissues (**Reviewer Figure 3**). While we are encouraged by this data, we think this is better suited to include in future studies that are directed toward further examining the mechanistic functions of the *Tug1* locus. But we included the following sentence in our manuscript.

**(lines 229-230)** *“Notably, these genes are downstream of the Tug1 TSS and located within the same*

*topological associated domain (TAD) in embryonic stem cells (Dixon et al., 2015, 2012).”*

**Reviewer Figure 2.** TUG1 and *cis* upregulated genes reside within the same Topologically Associated Domain (TAD) in human and mouse ES cells. Hi-C interaction heat maps in mouse ES cells (A) and H1 ES cells (B) showing the degree of physical interaction defined by Hi-C analysis for genomic region pairs from a 3Mb region of chromosome 11 (mm9) and human chromosome 22 (hg19). Tug1 TAD domain in mES spans through coordinates: chr11:3000000-4960000, and in human ES cells chr22:31200000-31960000 (Dixon et al., 2015).

**Reviewer Figure 3.** Capture HiC data (ChICP) in human megakaryocytes showing that human TUG1 locus interacts with the downstream gene SMTN. Data obtained from chicp.org.

**R2.7**. Similarly, given the large amount of ChIP data available for transcription factors, the authors could try to

establish candidate factors that bind this locus.

We thank the reviewer for this suggestion. Other groups have published on transcription factors that bind the

*Tug1* promoter including but not limited to, GATA1 (PMID: 31264779), SP1 (PMID: 26336870), and P53 (PMID:

24853421). We do highlight in the current manuscript that repressor proteins SIN3A and COREST are detected

at the human and mouse *Tug1* promoter and agree that understanding the transcriptional control of the *Tug1*

locus is of interest. However, this is out of the scope of our current study.

**R2.8**. Alternatively, how do the authors hypothesize that this DNA element operate on its targets?

Since the capture HiC data shows interactions with genes downstream of the *Tug1* TSS and analysis of ChIP

datasets (ENCODE) reveal that the *Tug1* locus is covered in H3K9 tri-methylation, we speculate that the loss of

this mark in the deletion model could be a mechanism by which the genes downstream of *Tug1* are

transcriptionally upregulated.

**R2.9**. Finally, are there human disease associated variants that impact the human ortholog of this putative

regulatory element? More specifically, are there sterility specific associated variants within the human locus?

Are there any of the affected genes associated with sperm production or maturation?

We thank the reviewer for this comment, as reviewer 1 also mentioned that providing more information on what

is known about *Tug1* and the dysregulated genes in the *Tug1* KO model could help better contextualize our

findings. Using previously published gene expression data, we found that *TUG1* RNA is significantly

downregulated in the sperm of sterile men (teratozoospermia) compared to fertile men (Platts et al. 2007). We

have included these results in a new **Extended Data Figure 7 (shown below)** and have added new text to the

**Discussion Section**:

**(lines 406-411)** *“Notably, there is some evidence that Tug1/TUG1 may have a conserved role in male*

*fertility in humans. Microarray profiling of sperm from sterile men (teratozoospermic) have significantly*

*less TUG1 expression compared with sperm from fertile males (Platts et al., 2007) (Extended Data Fig.*

*7). Thus, we speculate that the Tug1-/- model used in this study could potentially be of value for further*

*investigation into the functions of Tug1/TUG1 with relevance to human male fertility.”*

**Extended Data Figure 7. Loss of TUG1 expression in infertile human males.** Heatmap of

microarray data from three different probe sets showing decreased expression of TUG1 in sperm

from infertile teratozoospermic men compared to fertile individuals (normospermic) (in all cases p <

4.39 x10-4 ).

In addition, we previously searched the literature and noted male sterility phenotypes in a handful of genes

dysregulated in *Tug1* KO testes and this is included in the **Discussion Section:**

**(lines 483-491)** “*There is some evidence that the cis genes dysregulated upon deletion of the Tug1 locus*

*have a role in male fertility. Loss-of-function mutations in 2 of the 6 cis genes upregulated in Tug1-/- testes,*

*Smtn and Pla2g3, are characterized to have male fertility and sperm maturation defects (Niessen et al.,*

*2005; Sato et al., 2010). However, the impact of these genes on male fertility in an overexpression context*

*have not been reported. A role in male fertility for the other four upregulated cis genes upon deletion of*

*Tug1 (Gm11946, Rnf185, Selm, and 8430429K09Rik) have not yet been reported. In addition, there is*

*some evidence for a potential role of Tug1 RNA in male fertility. Mice with a loss-of-function mutation for*

*Selenop, a gene that was found significantly upregulated in Tug1-/- testes and significantly and*

*reciprocally regulated in Tug1rescue testes, is reported to have reduced male fertility (Hill et al., 2003).”*

Minor comments on this section:

**R2.10**. Figure 3A uses log2 which is reasonable to show fold change, why do the authors use the natural

logarithm in panel D?

The natural log for allele-specific RNA-seq expression is used because we performed the allele-specific

expression using a pipeline previously developed and published by our collaborator (PMID: 26140685).

**R2.11**. The Y scale in Fig3A should the same in all plots. Changing the scale for every tissue makes it very hard

to gauge the effect of the KO in different tissues.

We thank the reviewer for pointing this out. Our intent is to show the dysregulation on a per-tissue basis. We

have provided the specific fold change values in **Table S2** and **Table S3**.

**R2.12.a** Part III concerns the TUG1 RNA function. Here the authors follow a beautiful approach to isolate the

effect of the Tug1 RNA. They use a dox inducible transgene lacking the putative peptide inserted on the Tug1

KO background.

We thank the reviewer for acknowledging the thought behind the approach.

**R2.12.b.** Unfortunately, the experiment only partially worked, the transgene has lower expression than the WT

and even the heterozygous Tug1+/- individuals. This makes the rescue analysis challenging. Indeed figure S5

shows that there was no rescue of the phenotype, which, sure may require the RNA, DNA and the protein.

However, establishing which components are critical to the phenotype would shed much needed light over how

TUG1 functions. As presented, these data is confusing and not clearly interpretable. The negative result can be

equally likely be because the TUG1 RNA does not play a role in the observed phenotype, because the expression

of the RNA is too low, or because all three elements in the loci are necessary for proper sperm formation.

We not only agree with the reviewer but share their frustration. We certainly did not want to give the impression

we can make phenotypic conclusions based on the rescue experiment. We really could only make one positive

conclusion – that the small amount of transgenic *Tug1* did reciprocally regulate genes that were dysregulated in

KO and restored in rescue mouse. This result was encouraging in that it shows that the RNA is functional for

gene-regulation and is consistent with a previously proposed *trans*-based role for *Tug1* on gene expression

(PMID: 27760046).

We have added text to the **Results Section** to clarify the limitations of the rescue approach on fertility:

**(lines 317-319)** *“While this finding may suggest that the sterility phenotype is not due to the lncRNA*

*transcript, we note that the lack of a fertility rescue may also be due to the low levels of Tug1 expression*

*from the transgene in the testes or because a different Tug1 RNA isoform is required.”*

**R2.12.c.** Finally, in (Long et al., 2016) the authors go to great lengths (including performing ChIRP) to show that

TUG1 RNA functions by recognition of a AG rich DNA motif (figure 5C in that paper). The authors should report

whether this motif is enriched on rescue targets. The trans-acting mechanism of TUG1, if such exists, is likely

the same in the CNS and testis.

We thank the reviewer for suggesting this. We performed a motif enrichment analysis on the 52 reciprocallyregulated

genes (from TSS-0.5M to TSS+5K) that we identified in *Tug1* loss- and ectopic expression contexts in

the testes*.* Using MEME (http://meme-suite.org/tools/meme) that is commonly used for motif discovery, we found

four variations of AG motifs (**Reviewer Figure 4, below**). Long et al., identified AG- and AA- motifs throughout

the genome in a number of genomic features including LINE and SINE elements, LTRs, introns, intergenic

regions, and promoters and ultimately focused on characterizing a *Tug1* RNA binding region that was 400kb

upstream of Ppargc1a using a cell culture model. However, we caution to use their data to draw conclusions on

potential regulation between the *Tug1* binding regions identified in a culture podocyte model and genes we find

responsive to *Tug1* RNA levels in the testes. These genes may not be directly regulated by *Tug1* RNA as

assigning gene targets to DNA regulatory elements requires extensive *in vivo* follow up in the context of the role

of *Tug1* in the testes.

**Reviewer Figure 4: Motifs enriched in Tug1-responsive genes.** Motifs were searched in the promoter regions of the genes that are significant in *Tug1* KO and Rescue

Specific comments on this section

**R2.13**. Figure 4A shows a tree that seems the result of hierarchical clustering. The data underlying this tree

should be shown. Specifically, a heatmap with all replicates and differentially expressed genes should be shown in figure 1 or in supplementary material, such heatmap would clearly show the cell type specificity of dysregulated genes. I venture that such heatmap would render figure 4B redundant while at the same time being more informative.

As suggested by the reviewer, we included the heatmap corresponding to the hierarchical clustering data of

Figure 4A and have kept figure 4B. We present this new data in **Extended Data Figure 8 (shown below)** and

have now referenced this in the text:

**(lines 265-267)** *“We observed global changes in gene expression clustered by tissue-type, indicating*

*tissue-specific gene dysregulation (Fig. 4A, Extended Data Figure 8, Extended Data Tables 2-3).”*

**Extended Data Figure 8. Gene expression of multiple tissues in *Tug1* WT and**

**KO mice.** Gene expression of multiple samples (columns) were described with log

scale of TPM: Log2 (TPM+1). Rows (genes) are clustered by hierarchical clustering

with Ward’s method based on Euclidean distance. Annotation of samples are

provided in the top panel in terms of genotypes and tissues.

**R2.14**. It is unclear why the authors performed GSEA on differentially expressed genes. Did they intended to

write that they performed GSEA by sorting genes according to their fold change? DE test statistics? The method

describing this analysis is not clear and neither is the explanation in the text. Did the DE analysis lack power to

detect differences? Did the DE genes had no clear gene ontology enrichments?

We apologize for not clearly describing the performed GSEA analysis. We added the following new text in the

**Methods Section**:

**(lines 659-663)** *“We performed Gene Set Enrichment Analysis (GSEA) to evaluate the enrichment of the*

*gene sets available from MSigDB (Subramanian et al., 2005) after mapping genes to gene sets by gene*

*symbols. The statistical significance of a gene set was calculated with the test statistics of individual*

*genes computed by DESeq2. If the FDR-adjusted p-value is less than 0.1, the term was called as*

*significant. We did this calculation using the R package, CAMERA (Wu and Smyth, 2012).”*

Indeed, one analysis we performed did include GO-term analysis on the DE; however, most GO terms were

generic. We assumed that consideration of weak change of gene expression could reveal detailed gene

expression program changes and sought to identify the perturbed gene expression programs by performing

GSEA, a commonly used method, that considers the differential expression trend in transcriptome.

**R2.15**. It is unclear whether the two founder males had the tg(Tug1) transgene integrated in the same

chromosomal loci. Differences in the integration target may result in unwanted variability.

We agree with the reviewer and we do not know the integration site of the transgene and have stated this in the

Methods Section.

**(lines 528-530)** *“Two male founder mice containing random integration of the tg(Tug1) cassette were*

*identified by genotyping for the pTRE allele and individually mated to female C57BL/6J mice (Jackson*

*Laboratory, 000664) to expand the colonies.”*

**R2.16**. I do not think it suffices to provide the data in supplementary material. Full data for the rescue, KO and

WT testes should be shown.

We thank the reviewer for this comment. We provide histological sections of testes from *Tug1* mice along with

gene expression information on *Tug1* in WT, KO, and RESCUE. We include additional experiments and analyses

of *Tug1* transgene expression along with histology and sperm analysis as a supplementary figure. Because

different technical considerations could be responsible for the lack of a fertility rescue, such as the low levels of

transgene *Tug1* expression or the need for a different isoform, we feel it makes more sense to provide these

data grouped together in the supplementary section as opposed to the main figures.

**R2.17**. For example, a heatmap showing the most variable genes across all samples would help assess the

variability of the data and the specificity of the effect of each genotype. Such data would also help put figure 4G

in context. Figure 5 should label also the genes that are differentially expressed in each condition. There is no

way to assess how many genes are DE in the rescue compared to WT that are not DE in the KO compared to

WT. For this reason, the statement that 51 genes are rescued is not informative.

We thank the reviewer for this comment. For the reviewer’s information, we provide here a heatmap of 1000

variable genes in testes **(Reviewer Figure 5, below).** As suggested, we have now labeled the genes that are

19

differentially expressed in **Extended Data Figure 9 (shown below).** However, we did not want to focus on the

specific number of differentially-expressed genes since the level of rescue is mild, as the reviewer pointed out.

Instead, we showed a cohort of genes that are significant in both comparisons of WT vs. KO and KO vs. Rescue.

The overlap of significant genes in two comparisons are statistically significant (p-value <2.23-16, Fisher exact

test). Moreover, all the genes showing the statistical significance in both comparisons have opposite directions, revealing their reciprocal regulation by *Tug1* RNA.

**Reviewer Figure 5. Gene expression of highly variable genes in testes.** Gene expression of 1000 highly variable genes (rows) were described for testes samples (columns). Gene expression was quantified

with log scale of TPM: Log2 (TPM+1). Rows (genes) are clustered by hierarchical clustering with Ward’s method based on Euclidean distance. Annotation of samples are provided in the top panel in terms of *Tug1*

condition.

**Extended Data Figure 9. Changes of gene expression in two comparisons of KO vs. WT and Rescue vs. KO.**

Each dot represents a gene. Color describes statistical significance (adjusted p-value < 0.05): no significance

(gray, N=33688), significance in KO vs. WT (blue, N=998), significance in KO vs. Rescue (orange, N=126),

significance in both comparisons (red, N= 52).

**R2.18**. The resolution and size of Figure S5B make it impossible to understand.

Figure S5B contains flow cytometry plots that show the gating strategy for the isolation of B, T, and NK cells. We have updated this figure panel to include the antigen as well as the fluorophore on each axis in order to make the gating strategy clearer (**updated Extended Data Fig. 5 B**). On our end, we do not see an issue with the resolution or size of Figure S5, but we will communicate with Genome Biology about the quality issue.

**Extended Data Fig. 5 (B)** Representative flow cytometry gating strategy for NK, CD4, CD8 cells in peripheral blood

from WT, Tug1+/-, Tug1-/-, and Tug1-rescue mice (gating from WT peripheral blood shown).

**R2.19**. I find that the choice of using KO/WT in the X axis and rescue/KO in the Y axis of figure 4G to

unnecessarily complicates the figure. The goal is to test whether the rescue is similar to a WT, hence correlation

not anti-correlation of the differences between the WT and the rescue to the KO is the expected result if all works

well. Why not always have the KO in the numerator (or denominator)?

We understand the reviewer's comment. However, our point is not that the rescue is similar to WT, but that the

changes in expression seen in the rescue are reciprocal to the ones observed in KO. To better show this, we

have replaced Figure 4G with a heatmap, showing the significant reciprocally regulated genes **(new Figure 4G,**

**shown below):**

**Figure 4G.** Heatmap showing expression of reciprocally regulated genes in Tug1 KO/WT and Rescue/KO.

**R2.20.a.** Part IV concerns with the conserved open reading frame (ORF) within the 5' of the TUG1 loci. I find this

the least developed of all four parts. The authors convincingly show that one open reading frame in the 5' end of

the TUG1 loci is conserved, and that its coding potential is well conserved across evolution, they name it TUG1-

BOAT. This is a valuable point.

We thank the reviewer for acknowledging that identifying conserved ORFs in a lncRNA locus is a valuable point.

**Figure 4G.** Heatmap showing expression of reciprocally

regulated genes in Tug1 KO/WT and Rescue/KO.

**R2.20.b.** The authors, however fail to show that this protein is actually expressed in testes or at least that there

exists at Tug1 isoform that has an unspliced 3' UTR that will lead to a stable, translationally capable mRNA.

Indeed it is extremely likely that the three large introns upstream of the conserved ORF will doom the TUG1

mRNA to nonsense mediated decay, see for example (Lejeune and Maquat, 2005)). I find that it is critical to

show that this protein actually gets translated or at least that there exists and isoform that results in a viable and

stable mRNA encoding for this protein.

We thank the reviewer for these comments. Below, we address the strategies we used to detect endogenous

TUG1-BOAT and outline the text changes. To determine whether *Tug1* produces a stable RNA transcript

containing the TUG1-BOAT sequence, we performed RT-PCR on wildtype and Tug1 knockout testes. We were

able to amplify the 5’ region containing the ORF in mouse testis. We have added this new data to **Figure 5C**

**(shown below)** and have added the following text to the **Results Section**:

**(lines 345-352)** *“To determine if a transcript containing ORF1 (located in the 5’ region) is expressed in*

*testes, we performed reverse transcriptase PCR (RT-PCR) on wild type and Tug1-/- testes. Using a primer*

*set that spans ORF1, we detected a band at approximately 700 bp in the wild type sample with RT*

*enzyme and did not detect a product in the no RT enzyme or in the Tug1-/- control samples (Fig. 5C). As*

*a loading control, 7SK, an abundant snRNA (Gurney and Eliceiri, 1980; Wassarman and Steitz, 1991),*

*is detected in both wild type and Tug1-/- samples (Fig. 5C). Taken together, these results provide evidence*

*that the 5’ region containing ORF1 is expressed in testes and presents the possibility that this ORF could*

*generate the predicted protein.”*

**Figure 5: (C)** RT-PCR in wild type and *Tug1*-/- testes for ORF1. Schematization of Tug1 locus showing location of

primers (triangles) and ORF1 (red rectangle).

We thank the reviewer for providing a reference to help understand the possibility of *Tug1* RNA undergoing

nonsense mediated decay (NMD). We sought to further investigate if there is evidence that Tug1 undergoes

NMD. Two separate studies, one in mouse testes and the other in a human cell line, that either genetically

remove or knockdown NMD pathway components, did not find evidence that *Tug1* undergoes NMD (PMID:

27149259, PMID: 22369889). Together, we do not find evidence that *Tug1* transcripts undergo NMD.

**Figure 5: (C)** RT-PCR in wild type and *Tug1*-/- testes for ORF1. Schematization of Tug1 locus showing location of

primers (triangles) and ORF1 (red rectangle).

**R2.21**. 1) To understand the possible function of the TUG1-BOAT, the authors show that transfection of different

TUG1-BOAT carrying constructs leads to detectable protein with specific localization patterns. They show that

overexpression of TUG1-BOAT affects the mitochondria. I find this result hard to interpret because 1) The

physiological levels of TUG1-BOAT are unknown and even if it is expressed, overexpressing at much higher

levels than physiological levels may result in effects that do not represent the actual function of this protein.

We agree with the reviewer an endogenous role in the mitochondria is unknown. We have clarified this in several

areas in the manuscript to state that this is a predicted ORF and it is unknown whether this protein exists

endogenously. Further, we also state throughout the text that the mitochondrial membrane depolarization is in

an overexpression context.

**(lines 453-455)** *“We demonstrate that the 5’ region of the Tug1 locus contains an evolutionarily*

*conserved ORF that when overexpressed, impacts mitochondrial membrane potential.”*

**(lines 465-467)** *“Identifying endogenous and disease contexts in which Tug1 RNA and TUG1-BOAT are*

*detected and function will be important to further explore the potential link between Tug1 and the*

*mitochondria.”*

**(lines 491-494)** *“As for a role for the putative protein, TUG1-BOAT, there is currently limited evidence for*

*its endogenous existence. Thus, future work clarifying an endogenous presence and function for TUG1-*

*BOAT will be necessary to understand whether it contributes to the male fertility defect.”*

Importantly, we did take two separate approaches to determine whether endogenous TUG1-BOAT protein can

be detected (antibodies and targeted mass spec). Below, we outline these efforts.

**Generating antibodies:** We first sought to develop antibodies against TUG1-BOAT. We identified two antigenic

regions within the predicted protein and had two separate polyclonal antibodies generated (**Reviewer Figure**

**6A, shown below**). Antibodies against the two antigenic regions were affinity purified (using the two antigens).

We next performed dilution assays using the antibodies on protein lysate from cells transfected with a TUG1-

BOAT-3xFLAG-HA expression vector. In the lysate using an anti-FLAG antibody we detected TUG1-BOAT

(**Reviewer Figure 6B, shown below**); however, using multiple dilutions (2 μg/mL, 1 μg/mL, and 0.5 μg/mL) for

both anti-TUG1 antibodies we did not detect a band at the expected molecular weight (**Reviewer Figure 6B,**

**shown below**). We verified that the membrane contained TUG1-BOAT-3xFLAG-HA by stripping two blots that

corresponded to the 2 μg/mL dilutions and re-probed with anti-FLAG. Bands at the expected molecular weight

for TUG1-BOAT were identified (**Reviewer Figure 6C, shown below**). Furthermore, we collected 3 tissues

(heart, testes, and spleen) from wildtype (WT) and *Tug1* (KO) male mice and isolated protein. We reasoned that

any band detected in WT samples would not be present in the KO samples. We loaded protein from these

tissues, performed western blots, and used anti-TUG1-1 at 2 μg/mL. In our positive control lane that contained

overexpressed TUG1-BOAT-3xFLAG-HA lysate we did not detect a band that corresponded to the molecular

weight (MW) of TUG1-BOAT (**Reviewer Figure 6D, shown below**). Moreover, we did not detect a band at the

expected MW in any of the tissues (**Reviewer Figure 6D, shown below**). Thus, our efforts to generate

antibodies against endogenous TUG1-BOAT have not yielded an antibody that can recognize even

overexpressed TUG1-BOAT and aid in understanding the endogenous existence of this protein.

**Reviewer Figure 6:** Antibodies generated against the putative peptide TUG1-BOAT are unable to detect

TUG1-BOAT in an overexpressed or potential endogenous context. (A) The amino acid sequence of the

predicted mouse open reading frame (ORF) TUG1-BOAT is shown. Locations of antigens used for anti-

Tug1-1 (α-Tug1-1, grey) and anti-Tug1-2 (α-Tug1-2, red) primary antibodies are highlighted. (B) Initial test

of α-Tug1-1 and α-Tug1-2 primary antibodies to determine specificity and optimal dilution. 25 μg of total

protein lysate from 3T3 cells overexpressing 3xFLAG-HA tagged TUG1-BOAT was loaded in each lane of

an SDS-PAGE gel. The gel was transferred to a PVDF membrane, which was cut so one lane of protein

lysate was contained within a vertical strip of membrane. Six strips were used to test α-Tug1-1 and α-Tug1-

2 primary antibodies at 3 different concentrations (0.5, 1, and 2 μg/mL) while another strip was probed with

α-FLAG primary antibody as a positive control. α-rabbit and α-mouse HRP secondary antibodies were

used to detect α-Tug1 and α-FLAG antibodies, respectively. (C) The strips used for α-Tug1-1 (2 μg/mL)

and α-Tug1-2 (2 μg/mL) antibodies in panel B were rinsed with TBST and reprobed with α-HA primary and

α-rabbit secondary antibodies to confirm the presence of TUG1-BOAT on the membrane and as a control

for proper secondary antibody concentration. (D) Total protein was extracted from heart, testes, and spleen

tissues collected from wildtype (WT) and TUG1 knockout (KO) mice. 30 μg of total protein from each

mouse tissue was loaded in an SDS-PAGE gel with total protein lysate from 3T3 cells overexpressing

3xFLAG-HA tagged TUG1-BOAT as a positive control. The gel was transferred to a PVDF membrane and

probed with α-Tug1-1 at a concentration of 2 μg/mL (left). The membrane was stripped and reprobed with

α-FLAG to confirm the presence of overexpressed TUG1-BOAT on the membrane (right).

**Targeted LC-MS/MS:** We next sought to identify endogenous TUG1-BOAT by targeted mass spectrometry. To

this end, we overexpressed epitope tagged mouse TUG1-BOAT (TUG1-BOAT-3xFL-HA) in 3T3 cells. Protein

was extracted, immunopurified using Anti-FLAG® M2 Magnetic Beads (Millipore Sigma) and sent for LC-MS/MS

analysis (see additional methods below). Signature tryptic peptides that could be used for targeted identification

of endogenous TUG1-BOAT are shown (**Reviewer Figure 7A, shown below**). Using these signature tryptic

peptides, we sought to identify endogenous TUG1-BOAT in a variety of mouse tissues and wildtype 3T3 cells

(**Reviewer Figure 7B**). Unfortunately, signature peptides were only identified in overexpressed TUG1-BOAT-

3xFL-HA, while no tryptic peptides were identified in wildtype 3T3 cells or mouse tissues, including testes.

However, this lack of identification could be due to a variety of reasons, the most important being inadequate

enrichment of low expressed endogenous TUG1-BOAT. Even though we were unable to identify TUG1-BOAT

endogenously, we do not feel that these results are conclusive as a variety of factors could account for our lack

of detection.

**Additional methods for LC-MS/MS:**

**Sample preparation:** Excised gel pieces were destained in ammonium bicarbonate/50% acetonitrile

(ACN) and dehydrated in 100% acetonitrile. Disulfide bonds were reduced by dithiothreitol, and cysteine

residues were alkylated with iodoacetamide. Sequencing grade trypsin (Promega) was added 1:50 w/w

and the samples were digested overnight at 37 °C. The digestion was stopped by addition of 5% formic

acid. Organic solvent was removed in a SpeedVac concentrator. The peptide mixture was desalted and

concentrated on Thermo Scientific Pierce C18 Tip.

**Mass spectrometry analysis:** Samples were analyzed on an Orbitrap Fusion mass spectrometer

(Thermo Fisher Scientific) coupled to an Easy-nLC 1200 system (Thermo Fisher Scientific) through a

nanoelectrospray ion source. Peptides were separated on a self-made C18 analytical column (100 μm

internal diameter, x 20 cm length) packed with 2.7 μm Cortecs particles. After equilibration with 3 μL 5%

acetonitrile 0.1% formic acid, the peptides were separated by a 180 min a linear gradient from 6% to 42%

acetonitrile with 0.1% formic acid at 400 nL/min. LC mobile phase solvents and sample dilutions used

0.1% formic acid in water (Buffer A) and 0.1% formic acid in 80% acetonitrile (Buffer B) (Optima™ LC/MS,

Fisher Scientific, Pittsburgh, PA). Data acquisition was performed using the instrument supplied

Xcalibur™ (version 4.1) software. Survey scans of peptides precursors were performed in the Orbitrap

by scanning from *m/z* 375-1600 with a resolution of 120,000 (at *m/z* 200), an S-Lens RF Level of 30%, a

maximum injection time of 50 milliseconds, and an automatic gain control (AGC) target value of 4e5.The

instrument was set to run in top speed mode with 3 s cycles for the survey and the MS/MS scans. After

a survey scan, tandem MS was performed on the most abundant precursors exhibiting a charge state

from 2 to 7 of greater than 5e3 intensity by isolating them in the quadrupole at 1.2 Th. CID fragmentation

was applied with 35% collision energy and resulting fragments were detected using the rapid scan rate

in the ion trap. The AGC target for MS/MS was set to 1e4 and the maximum injection time limited to 35

ms. The dynamic exclusion was set to 12 s with a 10 ppm mass tolerance around the precursor and its

isotopes. Monoisotopic precursor selection was enabled. MS/MS spectra were extracted from raw data

files and converted into mgf files using a Proteome Discoverer Software (ver. 2.1.0.62). These .mgf files

were then independently searched against mouse database using an in-house Mascot server (Version

2.6, Matrix Science). Mass tolerances were +/- 10 ppm for MS peaks, and +/- 0.6 Da for MS/MS fragment

ions. Trypsin specificity was used allowing for 1 missed cleavage. Met oxidation, protein N-terminal

acetylation, and peptide N-terminal pyroglutamic acid formation were allowed as variable modifications

while carbamidomethyl of Cys was set as a fixed modification. Scaffold (version 4.8, Proteome Software,

Portland, OR, USA) was used to validate MS/MS based peptide and protein identifications. Peptide

identifications were accepted if they could be established at greater than 95.0% probability as specified

by the Peptide Prophet algorithm. Protein identifications were accepted if they could be established at

greater than 99.0% probability and contained at least two identified unique peptides.

**Reviewer Figure 7.** Targeted LC-MS/MS fails to identify TUG1-BOAT endogenously. (A)

Protein lysate from overexpressed epitope tagged TUG1-BOAT (TUG1BOAT-3xFLAGHA)

in 3T3 cells was used to identify signature peptides for targeted mass spectrometry

(see additional methods). Molecular mass, signature peptides identified, and percent

coverage of the protein sequence are shown. (B) 30 μg of total protein lysate for each

sample shown was run on an SDS-PAGE gel. After Coomassie staining, the kDa range

shown was cut from the gel and sent for LC-MS/MS. The expected size of the protein and

total hits (sum of the hits for each signature peptide) are shown.

We also agree with the reviewer that the localization and the mitochondrial effects could be due to

overexpression of the peptide, rather than its endogenous function. To clarify this, we have carefully edited the

text to remove any confusing statements.

**R2.22**. 2) It is unclear what this phenotype has anything to do with the sterility phenotype the authors set out to

understand

To clarify, we do not draw a conclusion that the mitochondrial phenotype observed when the predicted ORF

(TUG1-BOAT) is overexpressed in a cell-based assay is directly related to the male sterility phenotype in *Tug1*

knockout mice. Because a number of recent reports have demonstrated that lncRNA loci can function through a

variety of mechanisms or combinations thereof (DNA regulatory elements, RNA, act of transcription, and protein),

one goal of this study was to identify potential molecular modes present at the *Tug1* locus. In this study we

believe we uncovered an unexpected molecular complexity embeded in the Tug1 locus: two noncoding activities

(DNA *cis* repressor and RNA in *trans*) and potentially a third coding activity. We agree that we do not yet know

the molecular nature causing male sterility phenotype in *Tug1* knockout mice and believe this will take a number

of long-term and carefully planned *in vivo* studies to clarify. We have gone through the text to clarify that we are

not drawing a conclusion about the ORF overexpression phenotype and the male sterility phenotype observed

in *Tug1* knockout mice:

**(lines 57-62)** “*Using engineered mouse models and cell-based assays, we provide evidence that the*

*Tug1 locus harbors two distinct noncoding regulatory activities: (i) a cis DNA repressor that regulates*

*neighboring genes and (ii) a lncRNA that can regulate genes by a trans-based function. We also show*

*that Tug1 contains an evolutionary conserved open reading frame that when overexpressed produces a*

*stable protein which impacts mitochondrial membrane potential, suggesting a potential third coding*

*function*.”

**(lines 465-467)** *“Identifying endogenous and disease contexts in which Tug1 RNA and TUG1-BOAT are*

*detected and function endogenously will be important to further explore the potential link between Tug1*

*and the mitochondria, and determine whether the locus does contain a third coding function.”*

**(lines 491-494)** *“As for a role for the putative protein, TUG1-BOAT, there is currently limited evidence for*

*its endogenous expression. Thus, future work clarifying an endogenous presence and function for TUG1-*

*BOAT will be necessary to understand whether it contributes to the male fertility defect.”*

**(lines 500-502)** *“Therefore, going forward it will be important to investigate the individual and/or combined*

*roles of Tug1 DNA, RNA, and/or putative protein in male fertility as well as in disease contexts in which*

*Tug1 is altered.”*

**R2.23**) 3) It seems to contradict the beneficial mitochondria metabolic effects reported by (Long et al., 2016).

We thank the reviewer for pointing this out. The cDNA clone used to overexpress *Tug1* in their study (I.M.A.G.E

clone ID 4223183 ThermoFisher) does not contain TUG1-BOAT and thus could explain why Long et al., did not

observe an effect on mitochondria membrane potential as we have in this study.
